# Comparative Immunogenicity of HIV-1 gp140 Vaccine Delivered by Parenteral, and Mucosal Routes in Female Volunteers; MUCOVAC2, A Randomized Two Centre Study

**DOI:** 10.1371/journal.pone.0152038

**Published:** 2016-05-09

**Authors:** Catherine A. Cosgrove, Charles J. Lacey, Alethea V. Cope, Angela Bartolf, Georgina Morris, Celine Yan, Susan Baden, Tom Cole, Darrick Carter, Elizabeth Brodnicki, Xiaoying Shen, Sarah Joseph, Stephen C. DeRosa, Lili Peng, Xuesong Yu, Guido Ferrari, Mike Seaman, David C. Montefiori, Nicole Frahm, Georgia D. Tomaras, Wolfgang Stöhr, Sheena McCormack, Robin J. Shattock

**Affiliations:** 1 Centre for Infection, St George’s, University of London, London, United Kingdom; 2 Hull York Medical School & Centre for Immunology and Infection, University of York, York, United Kingdom; 3 Mucosal Infection & Immunity Group, Division of Infectious Diseases, Department of Medicine, Imperial College London, London, United Kingdom; 4 Infectious Disease Research Institute (IDRI), Seattle, WA, United States of America; 5 Medical Research Council, Clinical Trials Unit at UCL, University College London, London, United Kingdom; 6 Duke Human Vaccine Institute, Duke University Medical Center, Durham, NC, United States of America; 7 Department of Surgery, Duke University Medical Center, Durham, NC, United States of America; 8 Vaccine and Infectious Disease Division, Fred Hutchinson Cancer Research Center, Seattle, Washington, United States of America; 9 Statistical Center for HIV/AIDS Research and Prevention, Fred Hutchinson Cancer Research Center, Seattle, Washington, United States of America; 10 CAVD Neutralizing Antibody Laboratory, Beth Israel Deaconess Medical Center, Harvard Medical School, Boston, MA, United States of America; Public Health England, UNITED KINGDOM

## Abstract

**Background:**

Defining optimal routes for induction of mucosal immunity represents an important research priority for the HIV-1 vaccine field. In particular, it remains unclear whether mucosal routes of immunization can improve mucosal immune responses.

**Methods:**

In this randomized two center phase I clinical trial we evaluated the systemic and mucosal immune response to a candidate HIV-1 Clade C CN54gp140 envelope glycoprotein vaccine administered by intramuscular (IM), intranasal (IN) and intravaginal (IVAG) routes of administration in HIV negative female volunteers. IM immunizations were co-administered with Glucopyranosyl Lipid Adjuvant (GLA), IN immunizations with 0.5% chitosan and IVAG immunizations were administered in an aqueous gel.

**Results:**

Three IM immunizations of CN54 gp140 at either 20 or 100 μg elicited significantly greater systemic and mucosal antibodies than either IN or IVAG immunizations. Following additional intramuscular boosting we observed an anamnestic antibody response in nasally primed subjects. Modest neutralizing responses were detected against closely matched tier 1 clade C virus in the IM groups. Interestingly, the strongest CD4 T-cell responses were detected after IN and not IM immunization.

**Conclusions:**

These data show that parenteral immunization elicits systemic and mucosal antibodies in women. Interestingly IN immunization was an effective prime for IM boost, while IVAG administration had no detectable impact on systemic or mucosal responses despite IM priming.

**Clinical Trials Registration:**

EudraCT 2010-019103-27 and the UK Clinical Research Network (UKCRN) Number 11679

## Introduction

The need for a vaccine capable of reducing heterosexual transmission of HIV-1 via the female genital tract remains an urgent priority for curbing the epidemic in women. A key attribute of such a vaccine will be its ability to induce protective antibodies in the vagina and cervix that could prevent transmission of HIV to women of child-bearing age, without compromising fertility. This approach is supported by non-human primate (NHP) studies demonstrating that neutralizing antibodies can prevent vaginal acquisition when administered by intravenous infusion or applied topically to the vagina [1–4]. However, the relative importance of antibody levels in secretions versus mucosal tissue and the role of non-neutralizing antibodies in vaginal acquisition has yet to be fully defined [1,5]. The modest reduction in risk of HIV acquisition in the human RV144 “Thai” efficacy trial is thought to correlate with polyclonal non-neutralizing antibodies against the V1V2 region of gp120, in particular the IgG1 and IgG3 subclass, associated with antibody dependent cytotoxicity (ADCC). Interestingly, high systemic levels of envelope (Env) specific IgA targeting the same epitopes were directly correlated with risk, although mucosal levels of specific IgG and IgA were not determined [6, 7].

Different strategies for optimal induction of vaginal antibody responses have been explored in a number of animal models. These studies led to the concept of immunological linkage between the upper respiratory tract and lower genital tract [8]. For example, preclinical studies of intranasal (IN) immunization of mice with HIV gp140 were shown to elicit specific antibodies in vaginal secretions [9, 10]. Nasal immunization with CTB has been associated with vaginal antibodies in humans and induced stronger responses than those seen with direct IVAG immunization [11], although no comparison was made to parenteral vaccination. Currently the only examples of vaccine-induced protection against cervico-vaginal viral infection are the two licensed parenteral vaccines against human papillomaviruses [12]. These responses are assumed to be due to transudation of neutralizing IgG from the plasma into cervico-vaginal tissue and/or secretions [13, 14]. There has only been one previous clinical study of parenteral vaccination with recombinant gp140 alone (in the absence of DNA or viral vector priming), this included limited immunological analysis and did not assess vaginal antibody levels [15]. Data on the effects of direct vaginal vaccination in humans are extremely limited. In preclinical studies direct vaginal administration of gp140 in mice fails to induce local and systemic antibody responses, whilst in NHP this approach is partially effective and in rabbits it appears to be highly effective [9, 16, 17]. However, clinical studies of direct vaginal vaccination with gp140 in the absence of a parenteral prime have thus far failed to induce mucosal antibody responses [18, 19].

To the best of our knowledge this is the first comparative Phase I clinical trial in women to investigate the safety and immunogenicity of three HIV-1 clade C gp140 immunizations delivered by intramuscular (IM), intranasal (IN) and intravaginal (IVAG) routes with a specific focus on antibody responses to gp140 in cervico-vaginal secretions and in serum. The choice of a clade C immunogen was based on the high global prevalence of this HIV-1 subtype and in particular for its relevance to sub-Saharan Africa.

## Methods

### Vaccines

The recombinant clade C HIV-1 envelope gp140 protein (CN54gp140) is a naturally cleavage resistant, envelope clone of 97CN54 [20]. Recombinant CN54gp140 was manufactured to GMP specification [21] (Polymun Scientific, Austria) providing a product that was >80% trimeric, with a projected mass of 420 kD and a defined glycan profile [20]. For IM immunizations either 20 (IM20) or 100 μg (IM100) CN54gp140 was mixed with 5 μg Glucopyranosyl Lipid Adjuvant- aqueous formulation (GLA-AF) (IDRI, Seattle USA) [22] and administered in 0.4mls into the deltoid muscle. For IN immunization, 100 μg CN54gp140 was mixed with 0.5% (w/v) chitosan in 0.2ml (NovaMatrix, Norway) and dropped into each anterior nares [23]. For each IVAG immunization 500 μg CN54gp140 was given in a 1m aqueous gel vehicle (Particle Sciences Inc., USA) administered using an applicator directly into the vagina [19].

### Study design and conduct

**Fig 1:**
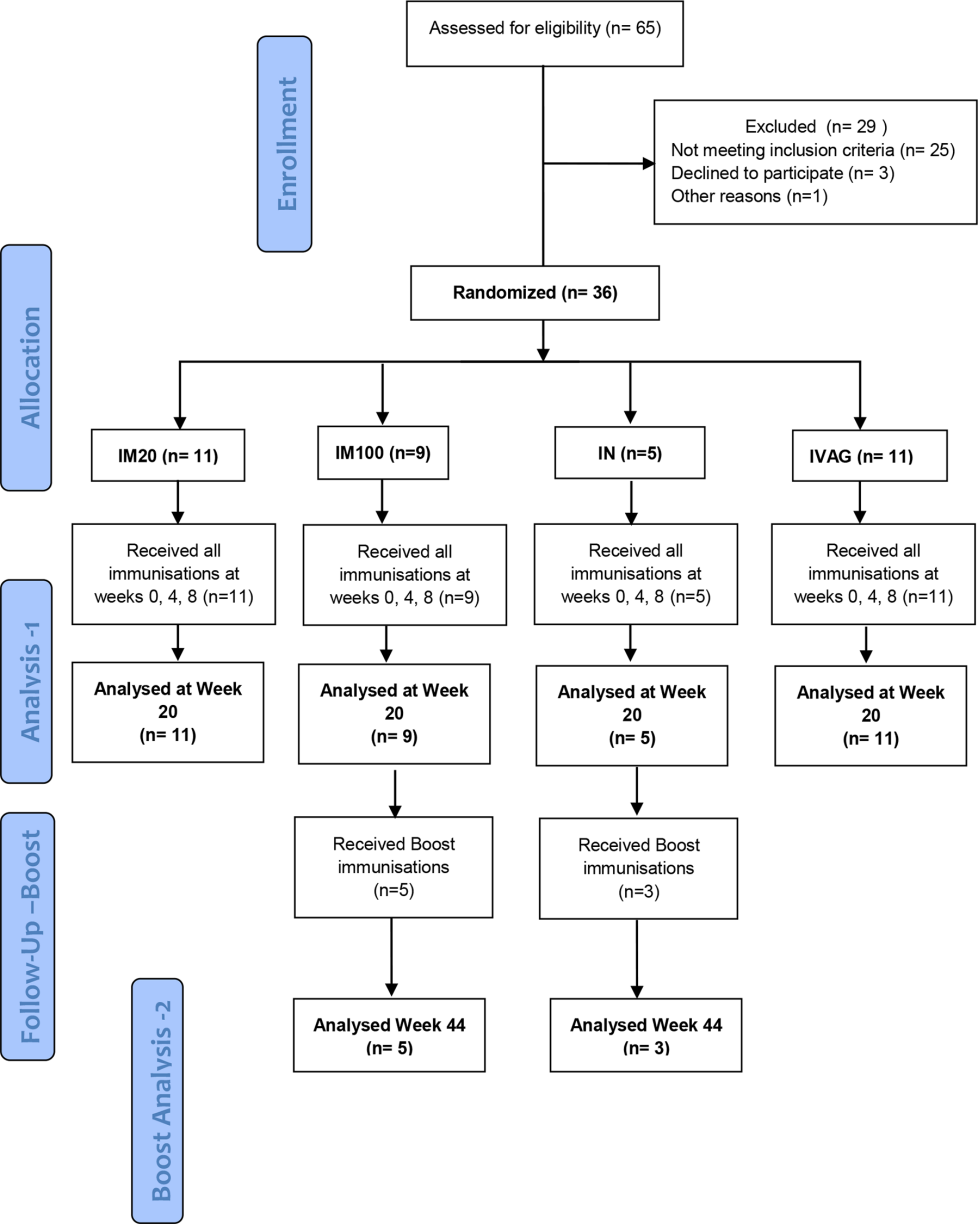
CONSORT Flow diagram. Numbers of participants recruited into the trial “Analysis 1”is the original protocol schedule to week 20 incorporating the planned main analysis. Five from IM100 and 3 from the IN group also received additional boost vaccinations following an amendment to the protocol, and these individuals were included in the exploratory “Boost Analysis -2”.

Mucovac2 was a Phase I randomized trial conducted at two centers; (St Georges University of London (SGUL) and Hull York Medical School, Experimental Medicines Unit (HYMS EMU). Volunteers were enrolled 17^th^ November 2011 to 7^th^ August 2012, follow-up for the study was completed on 11^th^ January 2013. All participants gave fully informed written consent and all relevant approvals were in place, the trial being conducted according to the UK Clinical Trials Regulations and GCP guidelines; reviewed and approved by NRES London Bridge Research Ethics Committee (S1 CONSORT Checklist). The trial was registered with the UK Clinical Research Network (UKCRN) assigned No. 11679 and the European Union Drug Regulating Authorities for Clinical Trials and assigned the EudraTC No. 2010-019103-27. The primary objective was to determine the safety and immunogenicity of the vaccination regimes in healthy female volunteers aged 18–45 years who were at low risk for HIV infection. The trial was open-label with laboratory assessment staff blind to regimen throughout and the participants blind to the dose administered in the intramuscular regimen. Eligible female participants were randomised centrally into one of the four groups using a computer-generated algorithm based on random permuted blocks stratified over one factor (clinical centres) aiming to enroll 10 individuals into each of the IM20, IM100 and IVAG groups and 6 into the IN group (as there were no responses following IN immunization in a preceding macaque experiment; at SGUL), 36 in total (Fig 1). Statistical Software Stata (StataCorp. 2011. Release 12. College Station, TX) was used by the trial statistician to create the randomization sequence with variable block sizes (3 and 6 for HYMS; 4, 8 and 12 for SGUL). Women in the IVAG group received a single IM prime (100ug) followed by two IVAG immunizations. Volunteers from the IM100 and IN groups were invited back to receive two further boosts of 100 μg gp140 adjuvanted with GLA-AF IM 4 weeks apart within 12–24 weeks of the last vaccination (full details in S1 Study Protocol).

### Safety, reactogenicity and adverse events evaluation

Information on adverse events was collected at every visit. Information on solicited adverse events was collected one hour post-immunization, one week later, and recorded in diary cards by the participants in the seven days following each immunization. The primary safety outcome was a severe or worse local or systemic adverse event or an event that led to discontinuation of the vaccine schedule.

### Clinical specimens

Serum, PBMC and mucosal samples were collected for immunogenicity at baseline, and weeks 4, 5, 8, 12, 16 and 20. Genital tract secretions were collected using the Instead Softcup™ (Evofem Inc) or Weck-Cel® surgical spears (Medtronic). Weck-Cel samples were also taken from the cervical os and vaginal fornices from all volunteers at screening and weeks 5 and 12. Mucosal secretion samples were acquired and processed as detailed in S1 Text.

### Assays of humoral immune responses

Serum and mucosal binding antibodies against recombinant CN54gp140 were measured using a standardized ELISA with minor modifications as described in the S1 Text. Serum specificity mapping was performed using a peptide epitope array of heterologous strains as previously described [24]. Neutralizing titers were measured in TZM-bl and ADCC activity was assessed as described [25, 26].

### Assays of cellular immune response

Intracellular cytokine staining (ICS) was performed as described previously [27]. Vaccine-induced T cell responses were analyzed using the mixture models for single-cell assays (MIMOSA) [28] statistical analysis to delineate vaccine-specific T-cell induced profiles. Multiplex bead array (MBA) to measure cytokine profiles of responding cells was conducted as previously described [29].

### Statistical analysis

Categorical variables were described by number, percentage and, where indicated, their 95% confidence interval (Wilson CI suitable for small sample sizes). Comparisons of categorical variables were made using Fisher’s exact test. The magnitude of an antibody response and other continuous variables were compared between groups using non-parametric tests (Wilcoxon matched-pairs signed rank test, or Mann-Whitney test). Association between continuous variables was examined using rank correlation. The level of statistical significance was 5% for all analyses, without adjustment for multiple comparisons. Statistical analyses were performed using Stata (Version 12.1).

## Results

### Participant accrual, safety and reactogenicity

36 individuals were enrolled and randomized into four groups. As a result of differential enrolment at the two sites, a small chance imbalance between the groups emerged with 11 participants allocated to IM20, 9 to IM100, 5 to IN, and 11 to IVAG groups. Additional boosting was given to 5 participants in the IM100 group, and 3 in the IN group (Fig 1). Demographic characteristics across randomization groups were balanced. There were no primary safety outcomes during the trial. After 3 immunizations, all 36 participants had experienced at least one mild or moderate solicited adverse event (Table 1). There were no significant differences in the median number of events per participant (p = 0.27) or in the maximum grade between groups (p = 0.61).

**Table 1:** Summary safety data–adverse events during main trial^¶^.

	IM 20 μg(n = 11)	IM 100 μg (n = 9)	Intranasal (n = 5)	IM + IVAG (n = 11)
**Primary Safety outcome:**	0	0	0	0
**SAEs:**	2*	0	0	0
**Total Solicited adverse events:**	69	39	24	62
**No. of events**; **Grade 1**	63	37	24	60
**No. of events: Grade 2**	6	2	0	2
**No. of subjects with any event**	11	9	5	11
**Median no. of events per subject**	6	4	2	5
**Max grade per subject: Grade** 1	8	8	5	10
**Max grade per subject: Grade 2**	3	1	0	1
**Total No. of subjects with local events**	11	7	5	9
**Injection-site**	10	7	0	9
**Nasal**	1	0	5	1
**Intravaginal**	4	0	0	4
**No. of subjects with systemic event**	8	4	1	8
**No. of subjects with lab event**	2	5	3	6
**Unsolicited adverse events**				
**No. of events:**	36	33	27	34
**No. of subjects with any event**	8	8	5	10
**Median no. of events per subject**	2	2	5	2

Table 1 footnotes:

**¶** before boosting phase; there were no grade 3 or 4 solicited adverse events.

* Two serious adverse events were reported, the first was a suspected (accidental) paracetamol overdose, the second, a Bartholin abscess. Since this individual was not in the IVAG group the cyst was considered unlikely vaccine related.

### Immunogenicity

#### CN54gp140-specific serum antibody responses

**Fig 2:**
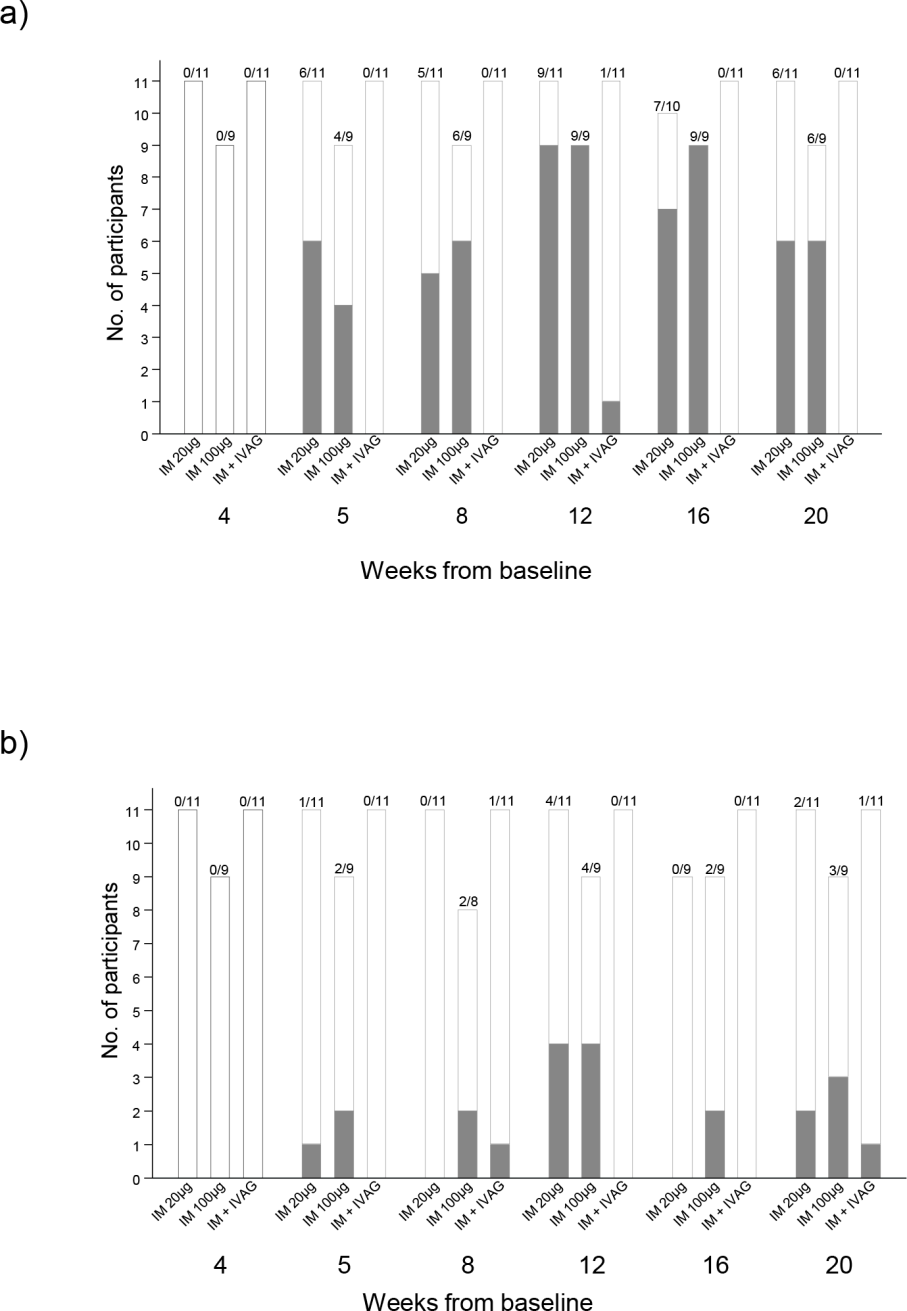
Participant response rates in systemic and mucosal compartments; Panel a) shows the numbers of participants with a CN54gp140 IgG antibodies in serum according to vaccination group and timepoint from randomisation. Panel b) shows the numbers of participants with CN54gp140 IgG antibodies in mucosal samples by vaccination group. Filled bars show the number of participants with detectable CN54 IgG, open bars show the number of participants with no CN54gp140 IgG antibodies.

**Fig 3:**
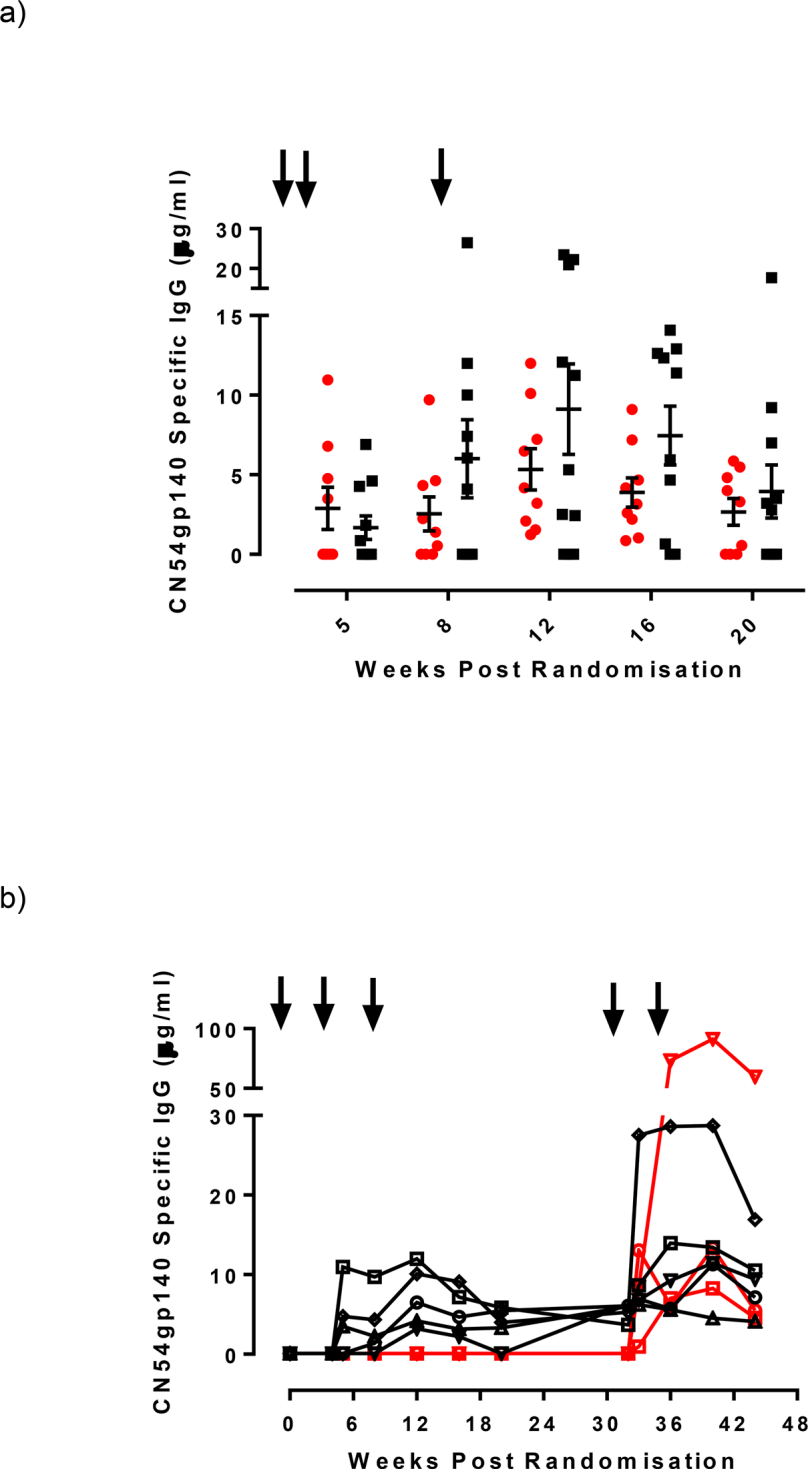
Magnitude of serum CN54gp140 IgG antibody responses for the two intramuscular vaccination groups by timepoint from randomisation. Panel a) Serum CN54gp140 IgG levels measured by ELISA expressed as μg/ml in either IM20 group (red circles) or IM100 group (black squares) at weeks 5, 8 12, 16 and 20. The black arrows indicate immunizations administered at weeks 0, 4 and 8 weeks. Panel b) Serum IgG responses measured in the 8 participants that received boost immunizations at weeks 32 and 34 (shown as arrows). Black symbols/lines indicate the 5 participants from the IM100 group, red symbols/lines indicate 3 participants from the IN prime group.

Systemic CN54gp140-specific IgG responses were detectable one week after the second vaccination (Fig 2A) in the two IM groups. The highest number of responders with a detectable serum IgG response across all groups (20/36; 55%) was at Week 12, 4 weeks after the third immunization. 9/11 responded (detectable IgG) in the IM20 group (median 5.32μg/ml; range 0.01–23.47 μg/ml) and 9/9 (median 4.18 μg/ml; 1.23–12.01 μg/ml) in the IM100 group, with no significant difference between doses (p = 0.71, Mann Whitney test). One individual in the IVAG group had a detectable serum IgG response at Week 12 only and there were no detectable responses in the IN group. At Week 20, 6/11 (54.5%) and 6/9 (60%) still had detectable serum IgG in the IM20 and IM100 groups respectively. Serum IgG antibody concentrations in responders from these two groups were comparable throughout the vaccination schedule and during the follow-up period (Fig 3A). There were no systemic vaccine-induced CN54gp140-specific IgA responses detected in any of the samples from any of the participants during the trial.

#### CN54gp140-specific cervico-vaginal antibodies

CN54gp140-specific IgG antibodies were detected in the cervico-vaginal secretions of eight individuals at Week 12 (Fig 2B and S1 Table). Of these, 4/11 (36%; 95% CI 15–65%) were in the IM20 group, and 4/9 (44%; 95% CI 19–73%) in the IM100 group. There were no responders in the IN or IVAG groups at Week 12. Responses were more reliably detected in Softcup samples than Weck-Cel (S1 Table). Across all time-points, CN54gp140-specific IgG was detected in only 1/11 participants in the IVAG group, and none of the participants in the IN group. At Week 20, 12 weeks after the third vaccination, 2 individuals in the IM20 group, 3 in the IM100 group, and 1 in the IVAG group still had detectable CN54gp140-specific IgG antibody (Fig 2B).

Only 1/36 trial participants, an individual in the IVAG group, developed a cervico-vaginal IgA response post vaccination, detected in the Softcup sample taken at Week 20 (0.157 μg/ml), but not detectable at any other time-point. CN54gp140-specific IgG was also detected in the Softcup sample at this time-point and at Week 8 in the absence of a serum IgG response. In addition, two other participants had low but detectable IgA binding antibodies prior to CN54gp140 vaccination thought to be polyreactive. In one of these, a response was also detected at Weeks 2, 16, and 20. Total IgG and IgA were reliably and reproducibly detected in softcup samples across all visits (S1A Fig), where the ratio of IgG:IgA was somewhere in between cervical os and vaginal Weck-Cel samples (S1B and S1C Fig and S2 Table).

In the majority of participants, detection of specific IgG antibodies in cervico-vaginal secretions corresponded with detection of specific IgG in serum (S2 Table). At Week 12, the 8 participants with vaginal specific IgG (Softcup or Weck-Cel) had significantly higher serum IgG titres than participants without detectable specific vaginal IgG (n = 8: median serum CN54gp140 IgG 10.67μg [1.23–23.47μg/ml] versus n = 11 median 2.47μg/ml [0.01–20.96μg/ml];p = 0.02 Mann Whitney test). Detectable specific vaginal IgG (Softcup) was significantly correlated with serum IgG titer (Spearman’s rank r_s_ = 0.79, p = 0.036). At Week 16 and Week 20, median serum IgG titres were also greater in those with cervico-vaginal IgG, but this was not significantly different. However, the systemic and mucosal levels were not necessarily correlated as there were individuals who had systemic responses of a similar magnitude in the absence of any corresponding mucosal antibodies.

#### CN54gp140-specific IgG responses after additional boosting

Eight participants, 5 in the IM100 and 3 in the IN group, received two further boosts delivered by IM immunization with 100 μg of CN54gp140 adjuvanted with GLA-AF, 4 weeks apart, within 12–24 weeks of the last vaccination. IM boosting elicited systemic IgG responses in all 3 participants in the IN group, which were not previously apparent. These were detectable after the first IM vaccination and were present at all 4 scheduled sampling time-points (Fig 3B). In addition, specific IgG antibodies were also detected in the cervico-vaginal secretions from 1/3 participants, albeit only at 2 Weeks post boost. Of the five subjects in the IM100 group who received the additional two IM boosts, augmented IgG levels were observed after the first IM boost which although sustained by the second boost did not show an apparent additional benefit (Fig 3B). 3/5 had detectable cervico-vaginal specific IgG after the boost immunizations. Two of these individuals had previously shown mucosal IgG antibodies during the main phase of the trial.

#### Serum peptide microarray analysis

**Fig 4:**
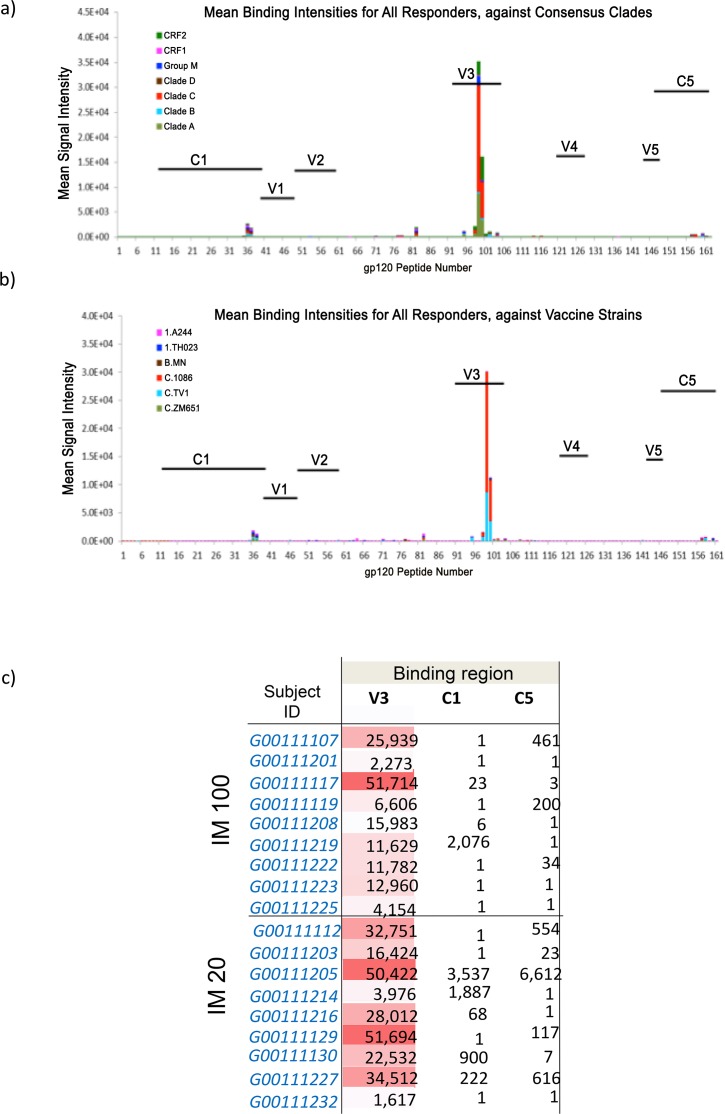
Peptide binding array analysis against gp120 peptides in serum samples from participants that developed a serum CN54gp140 IgG antibody response. Panel a) shows mean binding intensities against consensus clades; Panel b) shows mean binding intensities against a variety of HIV-1 vaccine strains. Panel c) Details the maximum binding intensities are shown according to participant at peak serum IgG response for the two groups that received intramuscular immunizations.

The specificity of induced serum binding antibodies was assessed by peptide microarray analysis. All responding subjects displayed predominant responses to the V3 loop (Fig 4A and 4B). A subset of vaccinees also developed low levels of C5 or C1 gp120 Env IgG responses (Fig 4C). There was no significant binding to gp41 Env (data not shown). The V3 gp120 IgG responses were focused on Clade C, with evidence of cross-clade reactivity (mainly against Clade A, CRF02, and Group M). Among the 3 clade C peptide sequences in the vaccine strain panel, the V3 response was targeted to 1086C and TV1 sequences. There was little correlation between the serum antibody titer and the number of epitopes recognized in the peptide array (Spearman’s correlation 0.42).

#### Neutralizing antibody activity and ADCC

**Fig 5:**
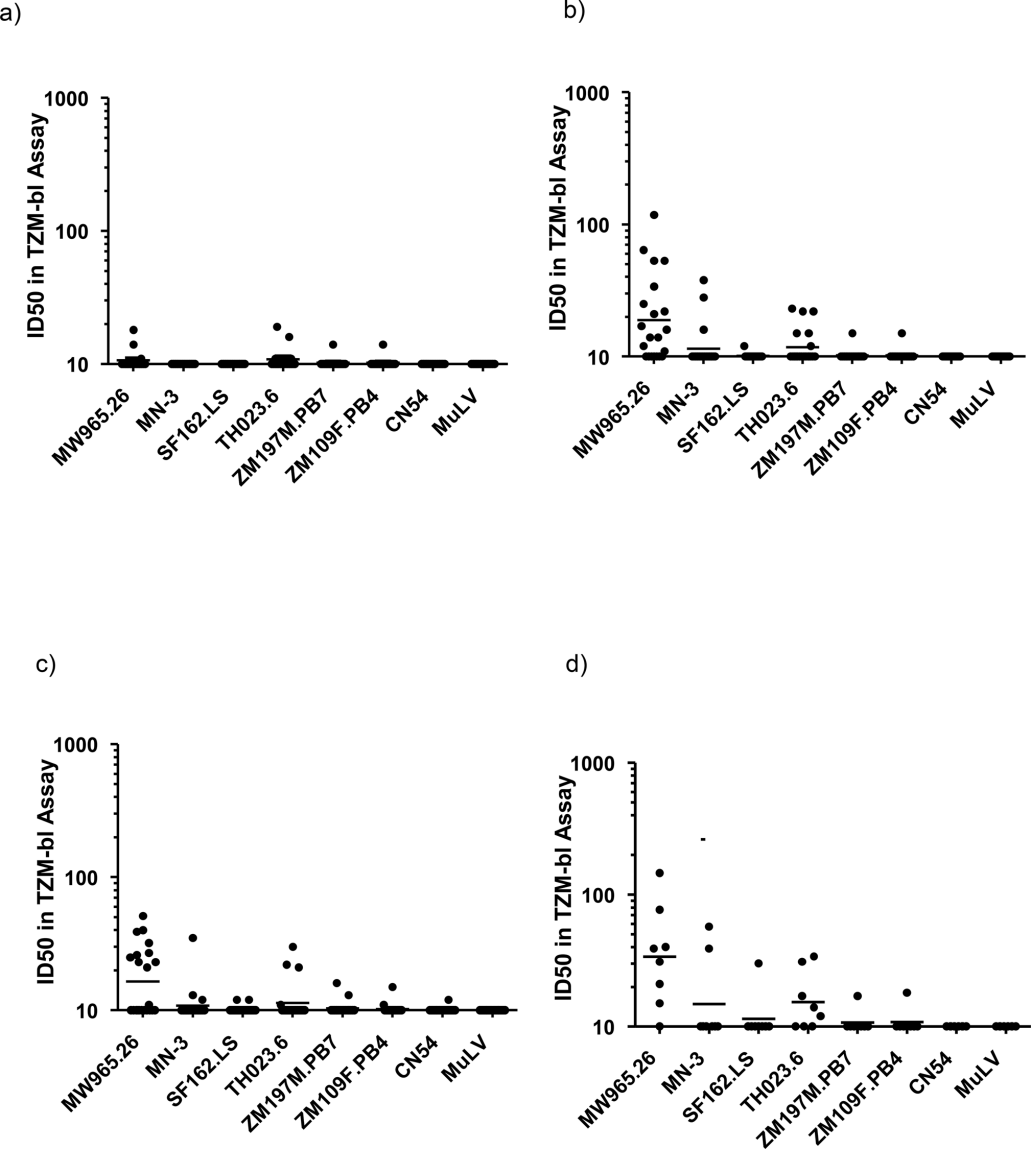
Serum neutralizing antibody titres, analysed by TZM-bl assay. **Data** expressed as ID50 against a variety of viruses at (a) week 0, b) week 12, c) week 20, and following additional boost in panel d).

Overall, neutralizing responses to tier 1 viruses were weak except for MW965.26 and TH023.6. Four weeks post third vaccination 7/11 individuals (87%) in the IM20 group, and 6/9 in the IM100 group (66.7%) showed responses against MW965.26, with no responses in the IVAG or IN groups (Fig 5A–5D). For individuals in the IN group that received additional IM boosting, response rate to MW965.26 increased from 0 to 67% (2/3). The mean global binding antibody titer moderately correlated with the neutralizing titer to MW965.26 (0.76, Spearman’s correlation), while binding intensity in the V3 region by peptide microarray positively correlated with neutralization titer for TH023.6 (0.92, Spearman’s correlation) and weakly for MW965.26 (0.50). There was no detectable ADCC activity in any of the samples exhibiting anti-Env antibodies four weeks after the third gp140 vaccination (data not shown).

#### Cell-mediated immune responses

**Fig 6:**
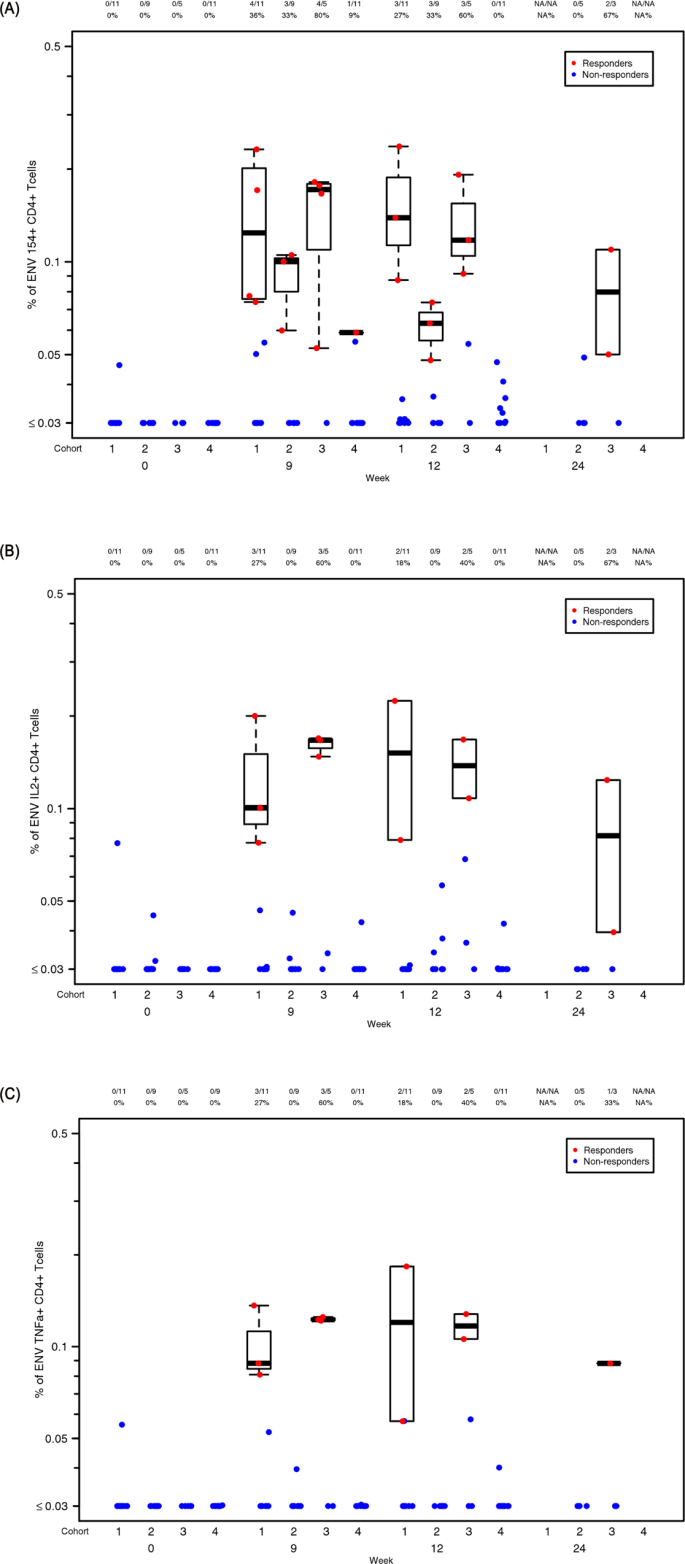
T cell ICS analysis. The graphs display background corrected magnitude of T cell response for each cytokine and T cell subset by visit as percentage staining shown as box plots. Here, cohort 1 = IM20, cohort 2 = IM100, cohort 3 = IN and cohort 4 = IVAG. Responders are colored red and non-responders blue. Box plots based upon data from responders only are superimposed on the distributions, mid-line denotes median,ends of the box denote 25^th^ and 75^th^ percentiles and where whiskers that extend from the top and bottom are the extreme data points. Panel a) shows staining of CN54gp140 specific live CD3+/CD4+ CD154+ cells, b) CD3+/CD4+ IL-2+, c) CD3+/CD4+ TNF-α+ according to vaccination group at weeks 0, 12 and 24.

T-cell mediated immunity was assessed by intracellular cytokine staining (ICS). There were no detectable cytokine responses in the CD8 T-cell population in any group during the study (data not shown). Interestingly, modest responses to Env were primarily observed in the CD4 cells from participants that received IN immunizations, despite mounting no detectable antibody response. Responses were greatest in this cohort one week post third immunization (Week 9), with 80% of individuals positive for CD154, 60% for IL-2 and 60% for TNF-α expression (Fig 6A–6C). By contrast, responses were infrequent, and of lower magnitude, in the IM20 parenteral group, and poorer still in the IM100 group. ICS responsiveness was not significantly associated with IgG antibody titer or neutralizing titer. Cytokine profiles were also measured by cytokine multiplex bead array (MBA) on supernatants of stimulated PBMC with whole CN54gp140 or matched peptides. Again, responses were predominantly detected in participants that received IN immunizations with poor or no responses detected in all other vaccination groups. In subjects where cells were available, the most consistent responses to whole CN54gp140 in the IN group were seen in 75% of participants at Week 9 for IL-2, IL-5 and IL-13 (S2A–S2C Fig), with weaker and less frequent responses for GM-CSF (2/4), IL-4 (2/4), IL-10 (2/4), and IL-17A (3/4 participants, data not depicted). Similar responses were seen for the IN group when PBMC were stimulated with matched peptides (two pools) in a Luminex assay. Here modest responses to most cytokines were seen in the IN group at Weeks 9 and 12, specifically IL-2, IL-15 and TNF-β, with weak responses across the other groups (S3A and S3B Fig).

## Discussion

We conducted a novel randomized phase I trial of an HIV-1 Clade C gp140 envelope protein comparing the safety and immunogenicity of IM immunization with two different mucosal routes: IN and IVAG. All the participants completed their immunizations and there were no severe adverse reactions reported (Table 1).

Serum CN54gp140 IgG responses were detected in the majority of participants in the two parenteral groups. There was no statistical difference between the groups, suggesting the lower dose of gp140 (20 μg) was similar to the standard dose (100 μg) when administered with adjuvant. There were no detectable serum IgA responses. This may reflect the adjuvant properties of GLA-AF. Given the potential negative correlation of specific systemic IgA in the RV144 trial [6, 7] this may be a positive characteristic of the GLA formulation. Detectable specific vaginal IgG (softcup) was significantly correlated with serum IgG titer, likely reflecting transfer of systemic antibody into the vagina by passive transudation [13, 14]. It is likely that the frequency of detectable vaginal antibody would be increased using strategies that elicit higher systemic responses. However, the systemic and mucosal antibodies were not necessarily correlated as there were some individuals who had systemic responses of a similar magnitude in the absence of any specific mucosal IgG, suggesting factors in addition to serum titer may influence IgG transfer to the vaginal lumen. The only previous clinical study of an HIV-1 subtype D gp140 envelope protein formulated in alum also showed antibody responses in all nine of the trial participants. It is not possible to compare data between two studies as only very limited immunological analysis of serum antibody response was reported using a clinical diagnostic ELISA with no indication of serum dilutions. Mucosal and cellular responses were not assessed [15].

Serum IgG responses were highly focused on the immunodominant V3 loop. This is reflective of a number of gp140 immunogens recently evaluated in non-human primate models [30]. V3 dominated responses were mirrored by the low level of detectable neutralization to closely matched tier 1 isolates. Binding intensity to the V3 region positively correlated to the neutralization of TH023.6 and only modestly with neutralization of MW965.26. Interestingly, as in rabbits and macaques [16, 17], serum antibodies did not recognize the gp41-immunodominant domain (residues 598–609) [31] and showed no significant binding to any part of gp41. This suggests that the gp41 region of the molecule may be occluded from immune recognition, potentially avoiding induction of diverting responses associated with gp41-cross reacting microbiota [32]. The lack of any detectable ADCC activity likely reflects lack of response to dominant epitopes associated with ADCC, typically V1/V2, C1 and gp41 [24, 33]. CN54 gp140 is naturally cleavage resistant constraining its ability to adopt a native-like conformation. It is anticipated that next generation stabilized cleaved soluble trimers may provide significant gains in neutralization breadth and potency [34].

Antibody responses to HIV-1 gp140 are thought to be dependent upon CD4 T- follicular helper (Tfh) cells. The paucity of detectable systemic CD4 T-cell responses in the two IM groups, despite robust antibody responses, suggests that required Tfh responses are likely restricted to the germinal centers of localized lymph nodes, and/or do not circulate in sufficient numbers at sampled time points (1 and 4 weeks post third immunization) to be detected. The observed paucity in CD4 response following parenteral immunization is in marked contrast to the IN group where 80% of participants had detectable circulating CD4 T-cell responses as assessed by CD154 expression (CD40L, a marker of T cell activation) in the apparent absence of detectable serum or vaginal antibody responses. It is unclear why we saw no effect of IN chitosan on humoral immunity, given that previous clinical studies have shown that chitosan promoted potent antibody responses to other immunogens [23, 35, 36], but it may be that these were intrinsically more antigenic. However, the induction of CD4 T-cell responses suggests effective uptake of CN54gp140 by local antigen presenting cells, and subsequent presentation to CD4+ helper T cells. These data contrast with early preclinical studies from our group in mice, which suggested that chitosan efficiently promoted both humoral systemic and mucosal responses to CN54gp140 [9, 37]. Interestingly, all participants in the IN group that agreed to the boosting protocol immediately responded to the first IM boost with serum antibody responses equivalent to those in the parenteral groups after three immunizations. Thus IN administration appeared to act as a mucosal prime for subsequent systemic immunization. It is unclear whether priming was restricted to induction of CD4 T cell help or included priming of antibody responses that were too low to be detected. However the rapid anamnestic response on IM immunization is supportive of a boosting of primed antibodies rather than de novo priming at inductive sites. The possible dependence on potential adjuvant properties of chitosan cannot be inferred in the absence of a control arm. It is likely that higher doses, and/or the inclusion of a more potent mucosal adjuvant, could provide more positive immunogenicity data, even during the priming phase [38].

Vaginal CN54gp140-specific IgG antibody was detected in 36% and 44% of volunteers in the parenteral IM20 and IM100 groups, and at low intermittent levels in one individual in the IVAG group. There was no evidence for induction of specific IgA responses in any group, including the IVAG group. We did however observe evidence of pre-existent, polyreactive CN54gp140-binding IgA in two individuals prior to vaccination, as previously described [39]. The lack of IVAG boosting following a single IM prime contrasts to our previous studies in macaques where a single IM immunization primed animals for induced antibody following IVAG immunization [13]. However, animals received 9 IVAG immunizations (100 μg each) over one menstrual cycle, as opposed to two higher doses (500 μg) used in this human study. Lack of responsiveness following IVAG administration may reflect the absence of mucosal adjuvant, prompted by our desire to minimize any local immune activation that might enhance HIV susceptibility [40]. However, in a different human study using CN54gp140 and HSP70 as an adjuvant, IVAG administration also failed to induce detectable systemic or mucosal antibody responses [18]. Furthermore, in recent studies a wider range of adjuvants failed to induce vaginal antibodies in NHP [38].

In summary, this study comparing different routes of administration demonstrates that IM administration of CN54gp140 elicited potent systemic IgG responses in the majority of subjects, and detectable vaginal IgG in just over a third. The observation that the lower dose of CN54gp140 (20 μg) was similar to the standard dose (100 μg) when formulated with GLA-AF adjuvant, provides opportunities for dose sparing regimes. Interestingly, IN administration induced detectable CD4 T cell responses and primed subsequent intramuscular boosting, while IVAG administration in the absence of adjuvant had no detectable impact on systemic or mucosal responses in spite of IM priming.

## Supporting Information

S1 CONSORT Checklist CONSORT 2010 Checklist MUCOVAC2. (DOC)

S1 Fig Measurement of total IgG (black symbols) and total IgA (red symbols) mucosal secretions. Data shown in either soft cup samples at weeks 0, 5, 8, 12, 16 and 20 (a) or cervical-os Weck-cel samples, (b) or vaginal vault Weck-Cel cel samples (c) at weeks 0, 5 and 12.
(TIF)

S2 Fig Cytokine Multiplex Bead Array (MBA) analysis measured in participants at weeks 0, 9 &12. Panel a) IL-2 responses, b) IL-5 responders are shown as red symbols and blue symbols non-responders. Here, cohort 1 = IM20, cohort 2 = IM100, cohort 3 = IN and cohort 4 = IVAG. c) IL-13 responders are shown as red symbols and blue symbols non-responders. Here, cohort 1 = IM20, cohort 2 = IM100, cohort 3 = IN and cohort 4 = IVAG.
(TIF)

S3 Fig Luminex analysis shown as Trellis Plots. Analysis was performed on samples at weeks 0, 9, 12 and during the boost phase at weeks 28 or 40 in samples stimulated with either peptide pool 1 panel a) covering half the sequence of CN54gp140 peptides 1–78. Responders are shown as red symbols and non-responders as blue symbols. Here, cohort 1 = IM20, cohort 2 = IM100, cohort 3 = IN and cohort. b) peptide pool 2 covering latter half the sequence of CN54gp140 peptides 79–169. Responders are shown as red symbols and non-responders as blue symbols. Here, cohort 1 = IM20, cohort 2 = IM100, cohort 3 = IN and cohort.
(TIF)

S1 Study Protocol MUCOVAC2 Clinical Study Protocol. (PDF)

S1 Table Cervico-vaginal specific IgG responses at Week 12 and corresponding serum responses. (DOCX)

S2 Table Total IgG and IgA levels (combined data from all groups) detected in cervico-vaginal secretions collected by Softcup or Weck-Cel sampling. (DOCX)

S1 Text Supporting Methods. (DOCX)
