# Supplementary figures and images for: Comparative Immunogenicity of HIV-1 gp140 Vaccine Delivered by Parenteral, and Mucosal Routes in Female Volunteers; MUCOVAC2, A Randomized Two Centre Study
Source: PLoS One. 2016 May 9;11(5):e0152038. doi: 10.1371/journal.pone.0152038 (PMC4861263; doi:10.1371/journal.pone.0152038)

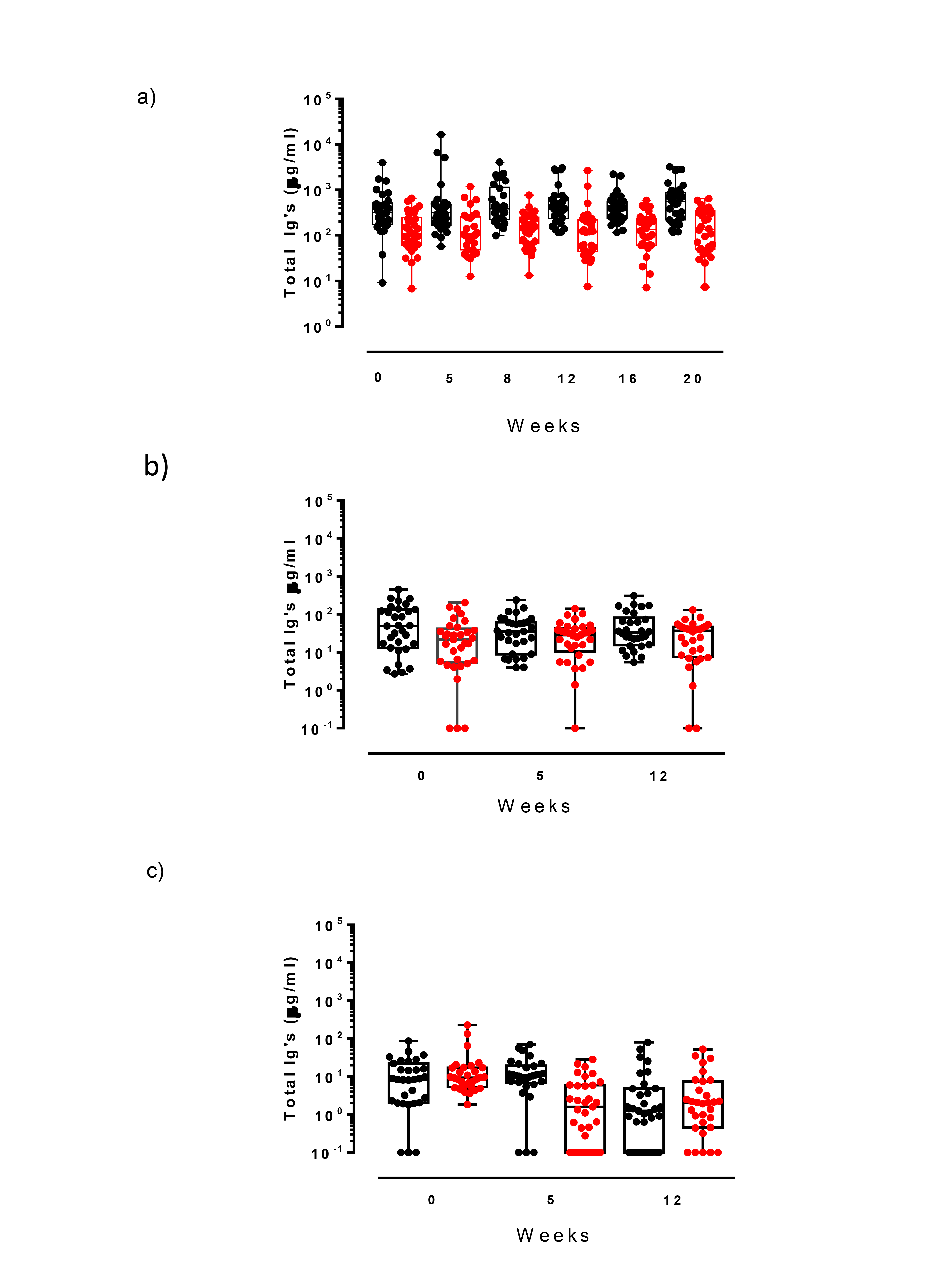

Supplement: S1 Fig — Data shown in either soft cup samples at weeks 0, 5, 8, 12, 16 and 20 (a) or cervical-os Weck-cel samples, (b) or vaginal vault Weck-Cel cel samples (c) at weeks 0, 5 and 12. (TIF) [file pone.0152038.s002.tif]

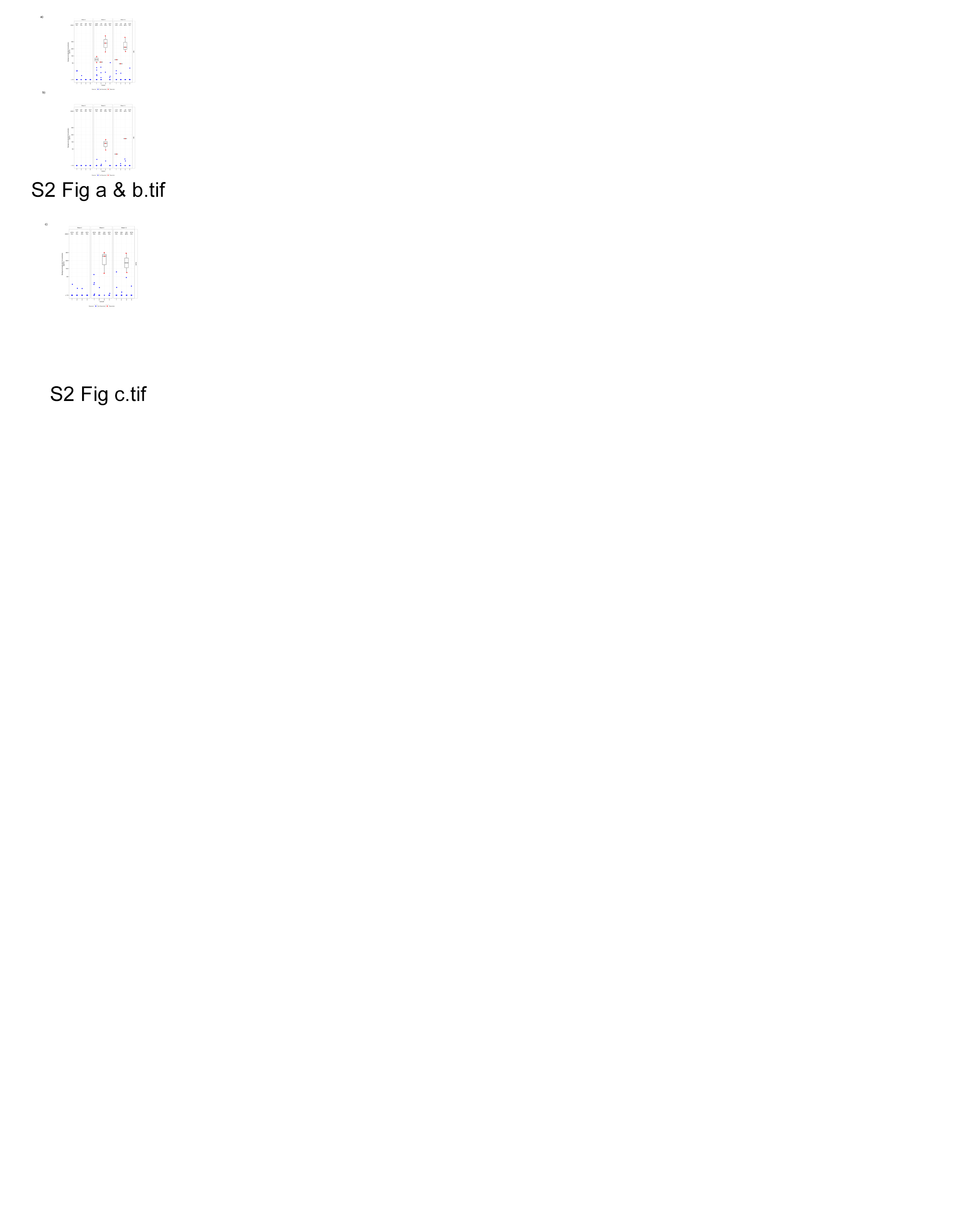

Supplement: S2 Fig — Panel a) IL-2 responses, b) IL-5 responders are shown as red symbols and blue symbols non-responders. Here, cohort 1 = IM20, cohort 2 = IM100, cohort 3 = IN and cohort 4 = IVAG. c) IL-13 responders are shown as red symbols and blue symbols non-responders. Here, cohort 1 = IM20, cohort 2 = IM100, cohort 3 = IN and cohort 4 = IVAG. (TIF) [file pone.0152038.s003.tif]

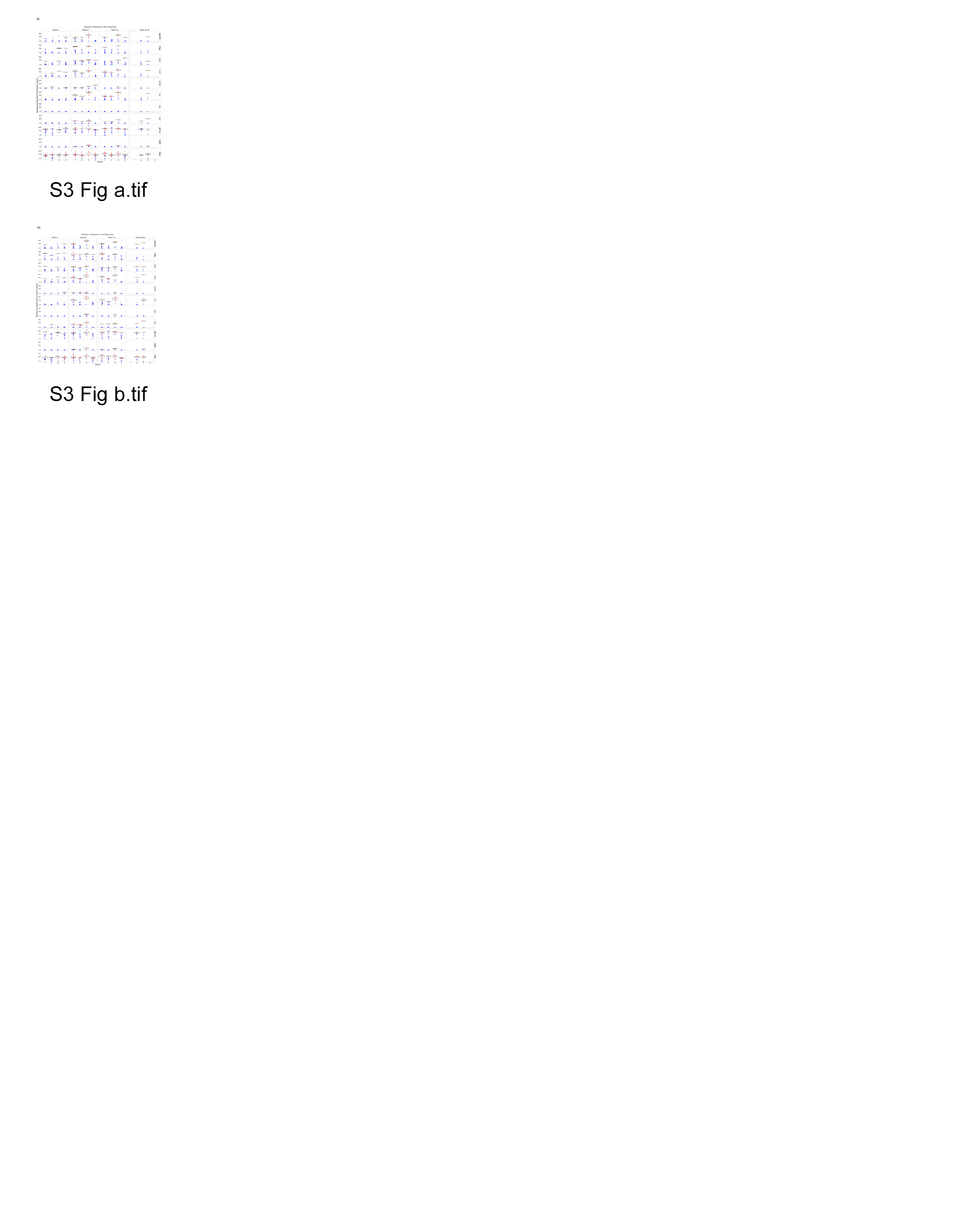

Supplement: S3 Fig — Analysis was performed on samples at weeks 0, 9, 12 and during the boost phase at weeks 28 or 40 in samples stimulated with either peptide pool 1 panel a) covering half the sequence of CN54gp140 peptides 1–78. Responders are shown as red symbols and non-responders as blue symbols. Here, cohort 1 = IM20, cohort 2 = IM100, cohort 3 = IN and cohort. b) peptide pool 2 covering latter half the sequence of CN54gp140 peptides 79–169. Responders are shown as red symbols and non-responders as blue symbols. Here, cohort 1 = IM20, cohort 2 = IM100, cohort 3 = IN and cohort. (TIF) [file pone.0152038.s004.tif]
