# Supplementary material for: Comparative Immunogenicity of HIV-1 gp140 Vaccine Delivered by Parenteral, and Mucosal Routes in Female Volunteers; MUCOVAC2, A Randomized Two Centre Study
Source: PLoS One. 2016 May 9;11(5):e0152038. doi: 10.1371/journal.pone.0152038 (PMC4861263; doi:10.1371/journal.pone.0152038)
Supplement: S1 Study Protocol — (PDF) [file pone.0152038.s005.pdf]

MUCOVAC2:

A Phase I clinical trial to assess the safety and immunogenicity of three HIV CN54gp140 immunisations administered through the intramuscular, intranasal and intravaginal routes in healthy female volunteers

EUDRACT 2010-019103-27

Protocol, version number 2.0

Protocol date 11.06.2012

(Based on MRC CTU template protocol version 3.15)

**Authorised by:**			
Name:	Dr Catherine Cosgrove	Role:	Chief/Principal Investigator,<br>St George's University London (SGUL)
Signature:		Date:	
Name:	Professor Charles Lacey	Role:	Principal Investigator,<br>University of York (UoY)
Signature:		Date:	
Name:	Dr Sheena McCormack	Role:	Medical Expert and co-Investigator,<br>Medical Research Council Clinical Trials Unit (CTU)
Signature:		Date:	
Name:	Professor Adrian Clark	Role:	Sponsor representative<br>St George's University London (SGUL)
Signature:		Date:	

GENERAL INFORMATION

This document describes the MUCOVAC2 trial and provides information about procedures for entering participants into it. Every care was taken in drafting the protocol, but corrections or amendments may be necessary. These will be circulated to the Principal Investigators in the trial, prior to implementation. Clinical problems relating to this trial should be referred to one of the Principal Investigators or the Medical Expert.

- **Compliance**

  The trial will be conducted in compliance with the protocol, UK Clinical Trial Regulations, principles of GCP, Data Protection Act (DPA number: Z5886415), NHS research governance and other regulatory requirements, as appropriate.

- **Sponsor**

  St George's University of London
  Cranmer Terrace
  London SW17 0RE
  Tel: 020 8672 5012

- **Funder**

  The Wellcome Trust under Grand Challenges in Global Health Initiative and the UK HIV Vaccine Consortium (UKHVC)

Main Contacts:

- **Chief Investigator/Principal Investigator, SGUL**

Dr Catherine Cosgrove MRCP PhD
Honorary Consultant in Infectious Diseases and General Medicine
St George's Hospital NHS Healthcare Trust
Blackshaw Road
Tooting
London
SW17 0QT
Email: catherine.cosgrove@nhs.net

St Georges Vaccine Institute
Ingleby House
Blackshaw Road
London
SW17 0QT

- **Principal Investigator, York**

Professor Charles J N Lacey, MD FRCP
Centre for Immunology and Infection
Hull York Medical School
University of York
York YO10 5DD
Tel: 01904 725423
email: Charles.Lacey@hyms.ac.uk

HYMS Experimental Medicine Unit
Learning And Research Centre
York Hospital
Wiggington Road
York
YO31 8HE
Tel. 01904 721893

Temporary Principal Investigator, York, 01/02/2012 to 29/02/2012 inclusive:

Dr Georgina Morris
Centre for Immunology and Infection
Hull York Medical School
University of York
York YO10 5DD
Tel: 01904 642116
Email: Georgina.Morris@hyms.ac.uk

- **Investigator and Medical Expert**

Dr Sheena McCormack, MSc FRCP

MRC CTU
Aviation House
125 Kingsway
London WC2B 6NH
Tel: 020 7670 4708 or 0755 411 4309
email: smc@ctu.mrc.ac.uk

- **Trial Management**

MRC CTU
Aviation House
125 Kingsway
London WC2B 6NH

Trial Manager and Monitor:

Liz Brodnicki
Tel: 020 7670 4783
Fax: 020 7670 4659
email: eb@ctu.mrc.ac.uk

CTU Project Lead:

Dr Sheena McCormack MSc FRCP
Tel: 020 7670 4708
Fax: 020 7670 4659
email: smc@ctu.mrc.ac.uk

Trial Statistician:

Wolfgang Stöhr PhD
Tel: 020 7670 4802
Fax: 020 7670 4659
email: ws@ctu.mrc.ac.uk

CTU Laboratory Expert:

Sarah Joseph PhD
Tel: 020 7670 4928
Fax: 020 7670 4659
email: sjo@ctu.mrc.ac.uk

- **Product Management**

Tom Cole
Department of Cellular and Molecular Medicine
St George's University of London,
Cranmer Terrace,
London SW17 0RE
Tel: 07980 650 679
email: tcole@sgul.ac.uk

Immunology Core Laboratories

Professor Robin Shattock PhD
Section of Infectious Diseases
Imperial College London
St Mary's Campus
Norfolk Place
London
W2 1PG
Tel 020 7594 3611
Fax 020 7706 0094
Email r.shattock@imperial.ac.uk

Dr Jill Gilmour
IAVI Core Immune Monitoring Laboratory
Imperial College London
Chelsea & Westminster Hospital
369 Fulham Road
London
SW10 9NH
Tel: 020 8746 5098
Email: j.gilmour@imperial.ac.uk

RANDOMISATIONS

Tel: 020 7670 4783 **(Mon - Fri, 08:30 – 17:00)**
Fax: 020 7670 4659

SAE AND IMPORTANT AE NOTIFICATION

Within 1 working day of becoming aware of an SAE or Important AE, please fax/email a completed SAE form 7 to the MRC Clinical Trials Unit on:

Fax: 020 7670 4659
Or email: HIVvaccine@ctu.mrc.ac.uk
Or phone: 020 7670 4783 **(Mon - Fri, 08:30 – 17:00)**

Serious Breach NOTIFICATION

Within 1 working day of becoming aware of a serious breach, please phone/fax details to the MRC Clinical Trials Unit on:

Tel: 020 7670 4783 **(Mon - Fri, 08:30 – 17:00)**
Fax: 020 7670 4659

CONTENTS

1 **Summary** 8
1.1 Abstract and summary of trial design 8
1.2 Flow diagram 11

2 **Background** 12
2.1 Background 12
2.2 Rationale and objective 18
2.3 Background and rationale for additional boosting 21

3 **Selection of Centres/Clinicians** 23

4 **Selection of Participants** 24
4.1 Participant inclusion criteria 24
4.2 Participant exclusion criteria 24
4.3 Number and source of participants 25
4.4 Screening procedures and pre-randomisation investigations 26
4.5 Screening procedures and investigations for boosting phase 26

5 **Randomisation & Enrolment Procedure** 27
5.1 Randomisation practicalities 27
5.2 Randomisation codes and unblinding 27
5.3 Co-enrolment guidelines 27

6 **Treatment of Participants** 29
6.1 Vaccine products 29
6.2 Clinical management of adverse events 32
6.3 Non-trial treatment 32
6.4 Issues related to HIV 33

7 **Assessments and follow-up** 34
7.1 Duration of follow-up and schedule 34
7.2 Assessments at Screening 34
7.3 Procedures for assessing immunogenicity 35
7.4 Procedures for assessing safety 36
7.5 Criteria for stopping treatment groups or whole trial 38
7.6 Procedures at the end of the trial 38

8 **Safety Reporting** 41
8.1 Definitions 41
8.2 Reporting adverse events 42
8.3 Pregnancy 43

9 **Withdrawal of participants** 45

10 **Statistical Considerations** 46
10.1 Method of Randomisation 46
10.2 Outcome measures 46
10.3 Sample size 46
10.4 Interim monitoring and analyses 47
10.5 Data analyses and presentations 47

11 **Data Management** 49
11.1 Data management at the Clinical Centre 49
11.2 Data management in the immunology laboratories 50
11.3 Data management at the MRC CTU 50

12 Trial Monitoring 51
12.1 Risk assessment 51
12.2 Monitoring at MRC CTU 51
12.3 Clinical site monitoring 51
12.4 Monitoring by the Trial Management Group 52
12.5 Confidentiality 52
12.6 Quality Assurance and Quality Control of Data 52
13 Ethical Considerations and Approval 53
13.1 Ethical issues 53
13.2 Ethical considerations 53
14 Indemnity 55
15 Finance 56
16 Trial Committees 57
16.1 Trial Management Group (TMG) 57
16.2 UK HIV Vaccine Consortium Steering Committee (TSC) 57
16.3 Independent Data Monitoring Committee (IDMC) 57
17 Publication 59
18 Protocol Amendments 60
19 References 63
Appendices 66

LIST OF TABLES

Table 1: Schedule of doses .... 8 & 31
Table 1a: Schedule of doses for boosts .... 8 & 31
Table 2: Solicited adverse events .... 9 & 37
Table 3: Schedule of visits 38
Table 3a: schedule of visits for boosts 40
Table 4: Source documents 49

LIST OF FIGURES

Figure 1: Flow diagram 11
Figure 2: Safety Reporting Flowchart 44
Figure 3: Diagram of relationships between trial groups and committees 58

APPENDICES

APPENDIX 1: PARTICIPANT INFORMATION SHEET 67
APPENDIX 1A: PARTICIPANT INFORMATION SHEET- BOOSTING SUB-STUDY 73
APPENDIX 2: CONSENT FORM 79
APPENDIX 2A: CONSENT FORM FOR BOOSTING SUBSTUDY 80
APPENDIX 3a: GP LETTER (pre-trial) 81
APPENDIX 3b: GP LETTER 86
APPENDIX 3c: GP LETTER BOOSTING PHASE 87
APPENDIX 4: TOXICITY TABLE 88
APPENDIX 5: DIARY CARD 91

ABBREVIATIONS AND GLOSSARY

**AE**	Adverse event
**AR**	Adverse reaction
**CF**	Consent form
**CI**	Chief Investigator
**CRF**	Case Report Form
**CTA**	Clinical Trial Authorisation
**ERC**	Endpoint Review Committee
**EUDRACT**	European Union Drug Regulatory Agency Clinical Trial
**GLA**	aqueous glucopyranosyl lipid A adjuvant : GLA-AF
**HYMS EMU**	Hull York Medical School Experimental Medicine Unit
**IAVI**	International AIDS Vaccine Initiative
**IB**	Investigator's Brochure
**ID**	Intradermal
**IDMC**	Independent Data Monitoring Committee
**IM**	Intramuscular
**IN**	Intranasal
**IVAG**	Intravaginal
**JRO**	Joint research office
**MHRA**	Medicines and Healthcare Regulatory Agency
**MRC**	Medical Research Council
**MRC CTU**	Clinical Trials Unit
**NHS**	National Health Service
**PI**	Principal Investigator
**PIS**	Participant Information Sheet
**R&D**	Research and development
**SAE**	Serious adverse event
**SAR**	Serious adverse reaction
**SGUL**	St Georges University London
**SOP**	Standard operating procedures
**SUSAR**	Suspected unexpected serious adverse reaction
**TMG**	Trial Management Group
**TSC**	Trial Steering Committee
**UAR**	Unexpected adverse reaction
**UKHVC**	UK HIV vaccine consortium
**UoY**	University of York

1 SUMMARY

1.1 Abstract and summary of trial design

1.1.1 Type of design

This is a Phase I, randomised, two-centre, exploratory trial of three immunisations with CN54gp140 vaccine formulations, administered in four different regimens. The laboratory staff will be blind to the regimen throughout and the participants will be blind to the dose administered in the intramuscular regimen.

IM boosting will be carried out in up to 10 volunteers (5 from the IN group and 5 from the IM 100µg group) who have completed their original vaccination schedule.

1.1.2 Disease/participants studied

36 healthy female volunteers 18 to 45 years old who are at low risk of HIV infection are to be recruited, approximately 18 from each site. For more details refer to section 4.

1.1.3 Trial interventions

The doses, formulations, routes and schedule of immunisation are described in Table 1 below. The 100µg CN54gp140 administered in 3 IM injections is the most conventional dose and route and can be considered the standard or control regimen. For more details refer to section 6.

Table 1 Schedule of doses, formulation and routes of immunisation

Group	Route of immunisation; dose of CN54gp140			Total cumulative dose of CN54gp140
	Dose 1 at wk0	Dose 2 at wk4	Dose 3 at wk8	
1<br>N=10	0.4ml IM<br>20µg CN54gp140<br>5µg GLA	0.4ml IM<br>20µg CN54gp140<br>5µg GLA	0.4ml IM<br>20µg CN54gp140<br>5µg GLA	60 µg CN54gp140
2<br>N=10	0.4ml IM<br>100µg CN54gp140<br>5µg GLA	0.4ml IM<br>100µg CN54gp140<br>5µg GLA	0.4ml IM<br>100µg CN54gp140<br>5µg GLA	300µg CN54gp140
3<br>N=6	0.4ml IN<br>100µg CN54gp140<br>Chitosan 0.5%	0.4ml IN<br>100µg CN54gp140<br>Chitosan 0.5%	0.4ml IN<br>100µg CN54gp140<br>Chitosan 0.5%	300µg CN54gp140
4<br>N=10	0.4ml IM<br>100µg CN54gp140<br>5µg GLA	3ml IVAG<br>500µg CN54gp140<br>Gel #2734	3ml IVAG<br>500µg CN54gp140<br>Gel #2734	1100µg CN54gp140

Table 1a – boost schedule (dose 4 & 5 should be 4 weeks apart)

Group	Route of immunisation; dose of CN54gp140		Total cumulative dose of CN54gp140
	Dose 4 at wk20-32	Dose 5 at wk24-36	
2<br>N=5	0.4ml IM<br>100µg CN54gp140<br>5µg GLA	0.4ml IM<br>100µg CN54gp140<br>5µg GLA	500µg CN54gp140
3<br>N=5	0.4ml IM<br>100µg CN54gp140<br>5µg GLA	0.4ml IM<br>100µg CN54gp140<br>5µg GLA	500µg CN54gp140

1.1.4 Objectives and outcome measures

Primary objective

To assess the safety and immunogenicity of three HIV CN54gp140 immunisations administered through the IM, IN and IVAG routes in a variety of combination regimens.

Outcome measures

Immunogenicity

- Presence and titre of anti-CN54gp140 IgG antibodies in cervico-vaginal secretions
- Presence and titre of anti-CN54gp140 IgG in the serum
- Presence and titre of anti-CN54gp140 IgA antibodies in cervico-vaginal secretions

Safety

- Grade 3 or above local solicited adverse event (Table 2)
- Grade 3 or above systemic clinical and laboratory solicited adverse event (Table 2)
- Any grade of adverse event that results in a clinical decision to discontinue further immunisations
- Any grade of adverse event that occurs in a participant that has received at least one immunisation

Table 2 Solicited adverse events

Route	Intramuscular	Intranasal	Intravaginal
Local AEs<br>(immunisation site)	Discomfort<br>Redness<br>Swelling (soft)<br>Induration (hard)<br>Blisters	Discomfort<br>Congestion<br>Discharge<br>Bleed	Discomfort (itching, burning)<br>Pain during sexual intercourse<br>Abnormal (unusual) discharge<br>Non-menstrual bleeding<br>Redness[1]<br>Swelling[1]<br>Sores/ulcers[1]
Systemic Clinical AEs	Temperature<br>Chills<br>Myalgia/flu-like general muscle aches<br>Malaise (excess fatigue)<br>Headache<br>Nausea		
Systemic Laboratory AEs	Creatinine, AST, ALT, alkaline phosphatase, total bilirubin, glucose<br>Hb, total WCC, neutrophils, lymphocytes, platelets		

1.1.5 Duration

It is anticipated that the first screenings will commence in the third or fourth quarter of 2010. Enrolment is anticipated to take 6 months and each subject's participation in the trial, from screening until final visit, a total of 10 visits, will be approximately 6 months. Therefore the end of the trial, defined here as the final visit of the last participant, should be achieved approximately one year after the start.

Up to 10 volunteers (5 from the IN group and 5 from the IM 100µg group) will be boosted with 2 further IM 100µg vaccinations 12-24 weeks after last priming vaccination and will be followed to a total of 15 or 16 visits. Visit 16 will take place if within the limits of funding.

1.1.6 Data recorded directly on case report forms (CRFs)

Data will be recorded directly onto the CRFs, which will provide the majority of source data for the trial. There will be some additional source data in the clinical notes, such as medical

history related to eligibility, dates of visits including immunisation, results of pregnancy tests, and details of clinical management (description of adverse events and concomitant medication). Participants will record information on solicited adverse events in a diary card which will act primarily as an aide memoire to be checked at the next clinic visit. The diary card data will be entered into the database hence will be considered to be source data. However, the investigators cannot be held responsible for the accuracy of the diary card. Where the information on the card is discrepant with data collected during a clinical visit, the grade that the clinician records will take precedence with the diary card finding footnoted. (see section 11)

1.1.7 Organisation

St George's University of London is the Sponsor of the MUCOVAC2 trial and trial co-ordination is being carried out by the Medical Research Council (MRC) Clinical Trials Unit (CTU). The project is funded by the Wellcome Trust under the Grand Challenges in Global Health initiative and the UK HIV Vaccine Consortium (UKHVC).

1.2 Flow diagram

Figure 1: Screening, randomisation, immunisations and follow-up

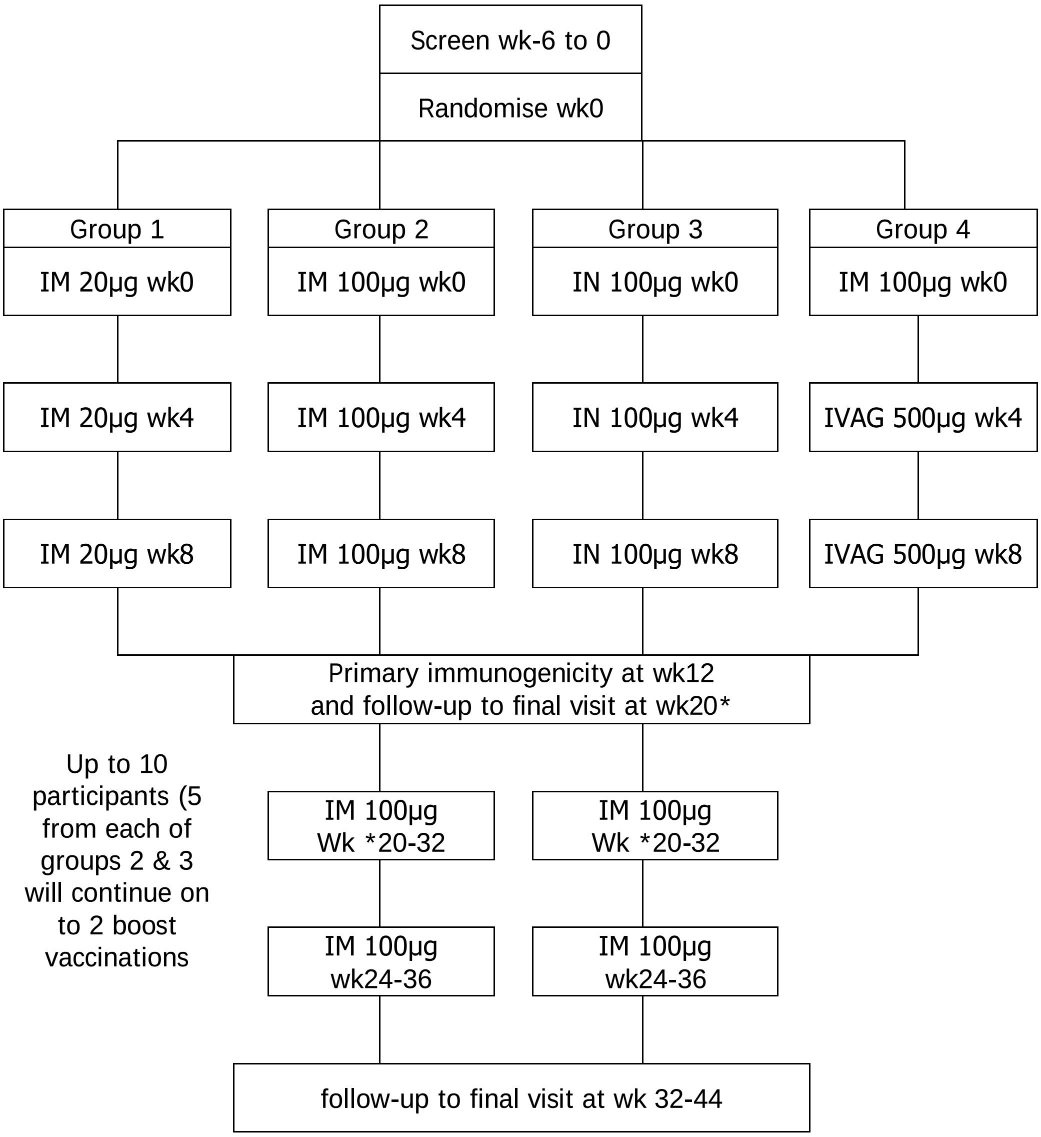

* for participants taking part in the boost schedule week 20 can also be 1st boost vaccination. Boosts should be given 4 weeks apart

2 BACKGROUND

2.1 Background

2.1.1 The global HIV-1 situation and the need for a vaccine

The global AIDS epidemic continues to grow. In its *2009 Report on the global AIDS epidemic*, UNAIDS published the following estimates for 2008:[1]

- 33.4 million people were living with HIV
- There were 7400 new infections per day
- Women were disproportionately susceptible to infection within sub-Saharan Africa, acquiring 60% of the infections
- Gains in expanding access to HIV treatment cannot be sustained without a reduction in the rate of new HIV infections

2.1.2 The status of the field

A prophylactic HIV vaccine is widely considered to be one of the most effective and sustainable ways of reducing the rate of new infections. The International AIDS Vaccine Initiative (IAVI) has estimated that a vaccine has the potential to prevent over 70 million infections in 15 years.[2]

The first clinical trials of candidate HIV vaccines started over 20 years ago and despite unrelenting effort and four efficacy trials, there had been virtually no good news until late last year when the “Thai trial” (RV144) reported a modest protective effect in a cohort of low risk, predominantly heterosexual individuals.[3] RV144 was a community-based, randomised, multicentre, double-blind, placebo-controlled trial consisting of four “priming” injections of a recombinant canarypox vector (ALVAC-HIV [vCP1521]) with two “booster” injections of a recombinant gp120 subunit vaccine (AIDSVAX B/E - given with the last two injections of ALVAC). The cohort recruited 16,402 healthy men and women between the ages of 18 and 30. In the modified intention-to-treat analysis involving 16,395 subjects (excluding 7 subjects who were found to have had HIV-1 infection at baseline), the vaccine efficacy was 31.2% (95% CI, 1.1-52.1; p = 0.04). Vaccination did not affect the level of viremia or the CD4+ T-cell count in subjects in whom HIV-1 infection was subsequently diagnosed. This modest effect was seen as a major step forward in vaccine research as it provided the first evidence that the development of a safe and effective preventive HIV vaccine was possible.

Initially the strategic focus was to identify and elicit protective antibodies, and when there was no demonstrable efficacy with this approach, the emphasis switched to immunogens that elicited T-cell responses. The first efficacy trials started in North America and Europe in 1998 and in Thailand in 1999 and assessed two versions of an HIV-1 envelope protein derived from gp120. In early 2003 preliminary results from the North American trial showed that whilst the product was safe and immunogenic, it failed to confer protection.[4] Results from the trial in Thailand (which also used AIDSVAX B/E), also indicated that the vaccine was safe but failed to confer protection.[5] Two large phase IIB trials of a “T-cell vaccine” (STEP and Phambili) using a non-replicating recombinant Adenovirus 5 vector containing the genes *gag*, *pol* and *nef* from subtype B were terminated due to futility and worryingly, there was also a suggestion of increased risk of HIV acquisition in some of those who received the vaccine although whether this increased susceptibility was caused by vaccine itself is still debated.[6,7]

2.1.3 The immune response to HIV

(i) The role of T-cells

Whilst CD4 T-cells are key regulators of the adaptive immune response, and therefore central to the generation and maintenance of both humoral and cellular immunity, CD8 T-cell responses have often been a focus of attention in vaccine development because they have the potential to kill virally infected cells. There seems no doubt that HIV peptide specific T-cell responses contribute directly towards protective immunity in those that are naturally infected. [8,9] The observation that the appearance of these cellular responses coincides with the control of acute HIV-1 viremia in both humans and macaques has been taken as evidence that T-cells are instrumental in controlling viral replication, especially as there is no such correlation between viral control the appearance of antibody responses. Recent work in animal models has suggested that recombinant viral vectors which stimulate a broad and strong CD8+ T-cell response can also partially protect against challenge with the highly virulent SIV mac239 strain something which had hitherto proved impossible.[10,11] When a cytomegalovirus vector is used to continuously deliver SIV antigens, systemic infection can be averted in some animals following intra-rectal challenge with SIV[12] perhaps highlighting the persistence of immune responses as central to vaccine efficacy. One area which is particularly poorly understood is why so many SIV and HIV-specific T-cells are so poor at killing virally infected (as opposed to peptide pulsed) target cells in vitro[13] and a deeper understanding of the relationship between the phenotype of a T-cell and its' protective potential is urgently needed. In this context, there is also pressing need for studies employing functional assays which better reflect what actually needs to happen during a natural infection and ideally these assays should be incorporated as endpoints in clinical trials.[14]

(ii) The role of antibodies

Animal studies have suggested that pre-existing neutralising antibody can prevent HIV infection but it is still far from clear how to artificially induce such responses. A significant minority of HIV infections result in heterogeneous mixtures of polyclonal broadly neutralizing antibodies to different epitopes on the virus envelope.[15] Several such broadly neutralising antibodies have been isolated from HIV infected individuals but to date, the antibodies elicited by vaccines have not been protective and many of the most relevant epitopes remain undefined. Some of the underlying reasons for this are now being understood. The target of the most potent neutralising antibodies, the envelope spike, is very unstable and difficult to synthesise in the relevant immunogenic form[16] and the most conserved epitopes within this envelope spike are some of the most difficult to access.[17] Even if it were possible to induce neutralising antibodies, access to infectious virions might also prove limiting. It is not yet known whether virus is transmitted in a predominantly free or intracellular form in vivo and it is also still far from clear precisely how the virus passes between cells. There is some doubt about any role for neutralising antibodies in processes such as synapse mediated transmission for example.[18] It is anticipated that the insights gained from the structural analysis of naturally occurring antibodies will feed directly into rational vaccine design hopefully accelerating the process.[19] Passive transfer experiments have already shown that realistically achievable levels of an existing broadly neutralising antibody can block infection following low dose intravaginal challenge with SIV - providing proof of concept for the strategy.[20,21]

2.1.4 Current vaccine strategies

Analysis of host responses natural and experimental infections suggest that that there are protective immune responses which could be harnessed by a vaccine.[22] Immune correlates of protection are still poorly understood, but it is widely agreed that broad and durable humoral and cellular immune responses are needed- ideally present at the portals of virus

entry. There has recently been a concerted effort to maximise vaccine immunogenicity within the T-cell compartment and several of the strategies currently under investigation are exploring heterologous prime boost regimens specifically with this in mind.[23,24,25] The rationale for the combined prime-boost regimen initially stemmed from pre-clinical studies which showed that priming with DNA followed by boosting with modified viruses induced stronger cellular immunity than either DNA or virus alone. DNA is thought to focus the immune response to the desired antigens, and the virus to boost this response, by expressing higher levels of the desired antigens and as a result of intrinsic adjuvanting properties.[26] The approach has proved partially protective in animal models and early phase trials in humans have reported particularly strong immunogenicity within the T-cell compartment and varying levels of success at within the B-cell compartment [23,24] In the past year the field has been reinvigorated by the success reported in RV144 and partly as a result, attention has turned again to the use of protein antigens derived from the HIV envelope as vaccines. Some of the earliest attempts to make vaccines focussed on this strategy but when used alone, such vaccines were not protective [27]. The lack of efficacy has been explained by the absence of a T-cell response and the failure of the vaccine used to induce relevant broadly neutralising antibodies. It is thought that this was due at least in part due to the conformation of the particular proteins used (monomeric) which were unable to induce broadly neutralising responses. By using a trimeric protein we hope to overcome these limitations and by using a mucosal adjuvant we hope to generate the appropriate response at the site where it is most needed. Several groups (UKHVC, HIVIS/TamoVac) are also planning to incorporate the same trimeric gp140 into existing prime boost regimens- with the aim of optimising systemic T-cell and antibody responses. The optimisation of immunogen design and modes/timing of vaccine delivery is currently the focus of several current vaccine networks (e.g EuroVacc, HIVIS/TamoVac, WRAIR, HVTN).

2.1.5 A role for immune responses at mucosal surfaces

Recent studies of acute HIV infection have shed some light on what is required of an effective vaccine. During the vast majority of natural infections, the antibody response which develops is “too little, too late” - occurring approximately 12 weeks after initial exposure which is after viral expansion has occurred.[28] Recent data also suggests that under normal circumstances, heterosexual transmission is a relatively rare event and that there is a small “window of opportunity” – in the order of days – during which it might be possible to stop the establishment of a latent infection. An ideal vaccine would prime a very early and broad antibody response targeting multiple neutralising epitopes for effective control of early viral replication Conventional ways of programming memory T- and B-cells to “remember” their encounter with antigen will probably not be sufficient to contain HIV infection because such responses will be too slow and novel immunisation strategies are needed [29]. Attention has turned to vaginal immune responses – because this is typically the first portal of virus entry in women. Both animal [30-32] and clinical studies have provided several lines of evidence that such local antibodies contribute directly to mucosal resistance to infection with HIV; (1) anti-HIV-1 specific IgA isolated from the mucosal secretions of HIV-infected patients are able to block HIV transcytosis *in vitro*;[33] and (2) the detection of neutralising IgA in highly exposed HIV-seronegative sex workers.[34] The work of our group and others has previously shown that topical vaginal application of antibodies with specificity for the envelope of HIV-1 is able to protect monkeys from subsequent challenge with virulent SIV providing further support for the approach.[35] It is far from clear how it will be possible to elicit and maintain sufficiently high levels of mucosal antibody in humans and it is believed that protection would require not only the generation of mucosal antibodies but also, the development of a cost effective strategy for the continuous maintenance of such responses - which is likely to be technically challenging.[36]

2.1.6 Research leading up to the proposed trial

(i) Animal studies

We have a long standing interest in mucosal vaccination and have completed several preliminary studies which directly inform the design of the study proposed here. We recently completed a study in mice which demonstrated the particular ability of Chitosan to adjuvant mucosal responses when delivered IN. Groups of mice (n=5) were immunised IN or ID with 20µg of CN54 gp140 in the presence or absence of Chitosan at 0,3, 6 weeks and antigen specific antibody responses were measured 3 weeks later both systemically and in vaginal lavage. High titres of systemic IgG and modest titres of IgA could be seen following ID vaccination with the protein alone and these responses were only marginally enhanced by Chitosan. Interestingly, there were negligible vaginal IgA responses following ID administration of this vaccine. The most dramatic effect of Chitosan was seen on the magnitude of vaginal IgA responses following IN vaccination. These hallmark mucosal antibody responses were only seen after IN vaccination and then, not in response to protein alone, but only when it was administered with Chitosan. The data support the relative superiority of the IN route for priming of IgA responses in the vagina in mice. The IN route also has obvious advantages for use in resource poor settings in that it is needle free and relatively non-invasive. In a GLP rabbit toxicity and tolerance study, done to support the dose levels and immunisation regimens, the vaccine formulations caused no systemic toxicity or unacceptable local side effects, and induced specific antibody responses.[37] The rabbit study explored the use of CN54gp140 given in combination with both chitosan and GLA and also included all the vaccination routes and regimens proposed here. However, because it was primarily a toxicity assessment, the study was not designed to generate endpoint immunogenicity data – which meant that the different vaccination strategies could not be formally compared. Nevertheless, all the regimens elicited CN54gp140 specific immune responses. These results form the basis of our proposal that the current protocol will be safe and is scientifically justified.

(ii) Phase I clinical trial (SG06RS02)

We have recently completed the first clinical trial of the protein in healthy women. SG06RS02 was completed in 2008 and was the first time that this vaccine (CN54gp140) has been administered to humans. This placebo controlled Phase I trial explored the safety and immunogenicity of 9 intravaginal vaccinations of 100µg CN54gp140 administered without adjuvant in 3.0ml carbopol gel to 17 healthy women at regular intervals over the course of one monthly cycle.[38] The vaccine was well tolerated and there were no serious adverse events in any of the 17 participants who started the course or the 11 who completed the regimen according to the protocol. A slightly higher proportion who received the active product experienced “local or systemic” adverse events (125 in total) with most falling into the categories of gastrointestinal, reproductive system/breast, nervous system or general disorders. The most commonly reported individual AEs were vaginal discharge, headache and metrorrhagia in that order. Whilst safe, vaccination via this route and in the absence of adjuvant was not immunogenic in this schedule and the results were disappointing. There were no gp140 specific IgA, IgG or T-cell responses detected in any of the serum or cervico-vaginal samples collected from those who completed the vaccination schedule. One positive T-cell response was detected in a sample from a participant 2 weeks after receiving her first immunisation but as she only went on to receive 8/9 vaccinations, the data were not included in the final analysis. The design of the proposed study will allow an exploration of the impact of giving the vaccine with GLA – which is a new adjuvant developed at the Infectious Disease Research Institute in Seattle which targets a toll like receptor on dendritic cells. In addition to rabbits the adjuvant has been shown to be well tolerated in humans[39] and rats[40].

2.1.7 Strategies to induce mucosal antibody responses

The study proposed here will allow a comparison of two vaccination strategies to the conventional regimen of 3 IM immunisations of 100µg CN54gp140 in the context of eliciting and maintaining cervico-vaginal antibody responses: firstly, a lower dose of 20µg CN54gp140 administered IM, and secondly an IM prime of 100µg CN54gp140 followed by 2 high dose IVAG immunisations of 500µg CN54gp140. The second strategic question builds upon promising data generated in macaques by other members of our team which suggested that IVAG vaccination with CN54gp140 was able to augment local immune responses following one round of IM vaccination.[41] Another study has also reported a similar HIV-1 subtype D-derived envelope protein to be well tolerated and immunogenic in all participants when administered IM at 100µg per dose in Alum.[42] It seems, however, that priming at mucosal surfaces such as the nose and the vagina might be more technically challenging perhaps requiring higher doses to prime a response. ANRS VAC14 was a recent Phase I trial which explored the safety and immunogenicity of 3 doses of 25µg or 50µg of MN/LAI (recombinant purified protein derived from gp160) administered via the nose or the vagina in the presence or absence of DC-Chol adjuvant at 4 weekly intervals. The vaccine was well tolerated under all circumstances and there were no vaccine related serious adverse events reported. Unfortunately there were no antibodies to gp160 detected in the serum, saliva, cervico-vaginal or nasal secretions from any of the participants.[43] The result was disappointing and directly contradicted what had been reported in Rhesus macaques when gp160 specific antibodies were detected in the serum in all animals and in vaginal, nasal and rectal secretions in some in when the vaccine was administered with adjuvant. Anatomical differences might have explained the discrepancy but it is more likely that the doses of vaccine administered in Phase I trial (ANRS VAC14) were suboptimal. The macaques received 5 monthly doses of vaccine simultaneously administered via the nose, vagina and rectum and although the doses per immunisation were the same as used in the volunteers, the doses of vaccine administered per kg body weight were clearly much higher in the animal experiment so the two were far from directly comparable. It is hoped that by the use of novel adjuvants and systemic priming followed by mucosal boosting, it might be possible to overcome the limited immunogenicity encountered previously.

2.1.8 Parallel macaque study and future work.

An identically designed parallel study has been initiated in cynomolgus macaques (January 2010). Animals (n=6 per group) were randomized to the same doses, formulations, routes and schedules of immunisation as described in Figure 1 (above), plus a fifth group which received a single IN prime and 2xIVAG boost. There were no detectable mucosal antibody responses at week 12 in the macaques receiving either of the regimens containing IN immunisations. We have therefore decided not to include the IN/IVAG combination, and to reduce the INx3 group from 10 volunteers to 6, as this will be sufficient to establish whether or not the lack of response in macaques is species specific.

Animals in which mucosal antibody responses were detected at week 12 will go on to receive additional IM protein boosts at weeks 24 and 36 to determine whether such responses can be sustained and/or increased. Should similar responses be observed at week 12 in human participants recruited to this study, it would be interesting to consider the option of additional IM protein booster immunisations at week 24 and/or 36. Immunised macaques will be challenged with infectious SHIV to determine if induced immune response provide mucosal protection which also will shed light on the responses most relevant to protection.

2.1.9 Investigational products/intervention(s)

There are four investigational medicinal products (IMPs) in this trial:

- The vaccine immunogen, CN54gp140
- Two vaccine adjuvants, GLA-AF and chitosan 1%
- An inert gel vehicle, Gel#2734

The vaccine immunogen

There is a general consensus that an effective vaccine against HIV-1 will induce neutralising antibodies directed to the viral coat protein, Env. The vaccine immunogen, CN54gp140, is a recombinant C-clade Env, derived from a Chinese viral isolate. The C-clade HIV subtype is believed to cause more than 50% of worldwide HIV-1 infections, and is predominant in southern and eastern Africa and India.[44]

The CN54gp140 immunogen is a recombinant protein manufactured using a mammalian cell expression system. It comprises a sequence of 670 amino acids, and has been shown to be immunogenic in non-human primates and other animal models. To date there has been just one human clinical trial of CN54gp140, EudraCT number 2007-000781-20, carried out at the same trial sites as the current trial. Healthy women were given nine intravaginal (IVAG) immunisations with CN54gp140 in a gel vehicle. The vaccine formulation did not cause any serious adverse events; however, only negligible immune responses were observed.[38]

The vaccine adjuvants

Adjuvants (immune potentiators or immunomodulators) are additives that have long been used to improve the immune response to vaccine immunogens, resulting in enhanced, accelerated and prolonged specific immune responses. Advantages of adjuvants include:

- enhancement of the immunogenicity of antigens
- reduction of the immunogen amount, and number of immunisations, needed for a successful vaccination
- modification of the nature of the immune response

In the current trial we will use two adjuvants: Protasan UP G 213, a chitosan glutamate, for intranasal (IN) immunisations; and GLA-AF, an aqueous glucopyranosyl lipid A adjuvant (GLA), for intramuscular (IM) immunisations.

Chitosan is a cationic polysaccharide comprising copolymers of glucosamine and

N-acetylglucosamine. It is usually manufactured as a deacetylation derivative of chitin obtained from the shells of crustaceans, and is widely used as a food additive and slimming aid. There are many types of chitosan salt, varying in degree of deacetylation and average molecular weight of the chitosan. Chitosan's IN adjuvant activity is believed to act mainly through its direct effect on epithelial cell tight junctions, which it opens transiently to allow enhanced paracellular penetration of immunogen across the nasal epithelial layer towards underlying antigen presenting cells. Protasan UP G 213 has been used in two previous clinical trials of IN immunisation conducted at St George's University of London.[45,46] In both trials participants received two IN immunisations containing 7 mg of chitosan powder, plus immunogen, 28 days apart. The immunisations were safe and well tolerated, with only transient, mild-to-moderate symptoms. The first of these trials[45] was designed to assess the adjuvant activity of IN chitosan, and the immune response induced by the chitosan-containing formulation was significantly greater than that induced by the immunogen in the absence of chitosan.

GLA-AF is an aqueous adjuvant formulation containing glucopyranosyl lipid A, a completely synthetic monophosphoryl lipid A (MPL®) like molecule. MPL is an adjuvant component of human vaccines licensed worldwide. Both GLA and MPL adjuvant are potent stimulators of antigen presenting cells through their binding and activation of toll-like receptor 4 (TLR4). GLA has been used in one previous clinical trial,[39] in which volunteers received one IM

immunisation with fluzone plus GLA formulated in emulsion (GLA-SE). Four concentrations of GLA in emulsion were studied (0.5, 1, 2.5 and 5μg). 5μg was administered to 4 individuals, 2 adults and 2 elderly. Three of four experienced multiple grade 2 and above adverse events, attributed to the formulation in emulsion. The lower concentrations were safe and well tolerated, with only transient, mild-to-moderate symptoms and signs, and GLA significantly enhanced the immune response to the immunogen.

The gel vehicle

Gel#2734 is an inert gel vehicle, specially formulated for administration of the intravaginal doses of CN54gp140.

2.2 Rationale and objective

We aim to induce durable immune responses at the vaginal and cervical mucosal surfaces of healthy women. The most commonly used route of vaccine delivery, IM, does elicit mucosal responses which correlate with, but are weaker than, systemic responses and not maintained for as long. Direct administration of protein vaccines to the mucosa of the female vaginal tract can result in the generation of local immune responses[47] but the first clinical trial of CN54gp140, in which healthy women were given nine intravaginal (IVAG) immunisations with the protein was very disappointing and only negligible systemic/local immune responses were observed. This lack of immunogenicity was most likely due to the insensitivity of the vagina to immunisation relative to other sites such as the skin – and is perhaps consistent with the physiological role of the vaginal tract as a natural barrier to infection. However, it's possible that the 9 consecutive doses were perceived as a single immunisation, and therefore we propose to administer 2 high doses, 4 weeks apart after an IM prime. The rationale for such a heterologous- prime boost regimen is supported by results generated in macaques as discussed above.[40]

In a GLP rabbit toxicity and tolerance study, done to support the doses and immunisation regimens described above, the vaccines were immunogenic within the B-cell compartment- resulting in the generation of CN54 gp140 specific Abs whilst not resulting in any worrying systemic toxicity or unacceptable local side effects.[37] These results form the basis of our proposal that the current protocol will be safe and is scientifically justified.

CN54gp140 doses

The nine doses given in the previous trial of CN54gp140 were administered over the course of a single menstrual cycle, the first immunisation about 7 days after the start of menses and the final one about 25 days after the start of menses. We now believe that from an immunological perspective the nine doses may have been 'seen' by the immune system as a single dose, since they were so tightly spaced. For this reason the IVAG immunisations in the current trial will be given four weeks apart.

In the previous trial of CN54gp140, each IVAG immunisation contained 100 µg CN54gp140, meaning that a total dose of 900 µg was given. 100 µg CN54gp140 per immunisation was chosen to harmonise with contemporaneous clinical trials of closely related recombinant Env proteins given via the IM and IN routes. The 100 µg IVAG doses were well tolerated, but it is now apparent that the vagina is a relatively insensitive site for immunisation, and we will increase the IVAG dose of CN54gp140 accordingly, to 500 µg per dose.

For the IN and IM immunisations we have chosen to stick with 100 µg CN54gp140, since this dose has provided good immunogenicity in other vaccine trials of recombinant Env proteins. In a recent Phase I trial of a multi-protein recombinant vaccine which included varying doses of monomeric gp120 (5,20,100μg) administered IM with a constant dose of a NefTat protein in the adjuvant AS02A, titres of binding and neutralising antibody to gp120 were independent of the dose of gp120 given and were of equal durability[48]. In light of these results and also our own experiences in animal studies, we believe the adjuvant GLA-AF (IM) also has the potential to augment the response to the immunogen Since the study of Goepfert reported maximal antibody responses to 5 µg of gp120 injected with NefTat, we

propose to assess immune responses to two doses CN54gp140 and are confident that the choices are justified. Group 1 will received three injections of 20 µg and Group 2 three injections of 100 µg CN54gp140 IM with GLA-AF.

The maximum cumulative dose of CN54gp140 is 1100 µg, in Group 4.

Chitosan dose

Each IN dose of 0.4ml (0.2ml/nostril) will contain 0.5% (w/v) chitosan glutamate; therefore 2mg chitosan will be administered per dose. This is less than the 7mg doses of chitosan glutamate administered in previous trials, during which only mild-to-moderate, transient adverse events were reported. Animal experiments suggest that 0.5% chitosan will have good adjuvanticity.

GLA dose

Good adjuvanticity was observed in a previous clinical trial of 2.5µg GLA in an oil-in-water emulsion formulation (GLA-SE), which was safe and well tolerated. Much of the reactogenicity of GLA-SE is believed to be associated with its oil-in-water form, so the aqueous formulation GLA-AF should be less reactogenic. Animal studies show that GLA-AF has broadly equivalent adjuvant activity to GLA-SE. Based on these findings we have chosen for the current trial a 5µg dose of GLA, which should approach optimal adjuvanticity while being safe and well tolerated.

2.2.1 Hypotheses to be explored and possible next steps

To assess the safety and immunogenicity of three HIV CN54gp140 immunisations administered in regimens that differ in dose and route.

This is a Phase I trial, and as such statistically significant results are not expected a priori. The following hypotheses will be explored with a view to informing the design of the next trial both in terms of size and selection of regimens:

- The lower IM dose is similar to the conventional IM dose in terms of the proportion of participants with detectable IgG or IgA antibodies in cervico-vaginal secretions, and the strength of response (titre) 12 weeks after enrolment
  - ⇒ If the lower dose is similar the next step would be to formally assess this in a larger trial
- Priming the immune system via the IM route and administering high IVAG doses of CN54gp140 in a schedule that allows for time to develop immunological memory will lead to detectable IgG or IgA antibodies in cervico-vaginal secretions in 3 or more participants that complete the schedule
  - ⇒ If less than 3 participants have detectable mucosal antibodies, there will be no further exploration of the IVAG route of administration with this product
- That IN administration of 3 CN54gp140 at conventional doses will elicit mucosal immune responses in 2 or more participants that complete the schedule, even though the responses in macaques were negligible
  - ⇒ If 2 or more participants have responses, we will be more cautious about extrapolating from the macaque model to the human one; if there are similarly negligible responses in human, then the macaque model will help select product for the IN route in future.
- That each of the routes and combination regimens is safe and acceptable
  - ⇒ If any of the routes or regimens have unacceptable safety defined in terms of the proportion of participants that experience a grade 3 or above solicited adverse event or an event that leads to an investigator decision to

discontinue immunisations, then there will be no further exploration of the route with the product/regimen.

2.2.2 Risks and benefits

This is a Phase I exploratory study in healthy female volunteers.

The CN54gp140 is being administered systemically for the first time in humans. However, there are extensive safety data on similar recombinant HIV protein vaccines, including from the large Phase III VaxGen trials,[4,5] and no additional concerns have arisen in the toxicology which was conducted in rats and rabbits. Volunteers in UK received up to 4 immunisations of DNA or NYVAC carrying the CN54 derived genes in three preceding vaccine trials without concern (EuroVacc 01, 02 and 03).

The GLA and chitosan adjuvants have been used in previous clinical trials, and appear to be well tolerated. The investigators do not feel dose escalation is warranted in the proposed trial as chitosan will be applied at a lower concentration than preceding trials, and the GLA adjuvant does not contain the oil and water emulsion which is recognised to be the main component causing local reactions.

Although the IVAG dose is higher per dose, the adverse events experienced in the preceding trial were probably precipitated by the volume of gel that was administered in repeated doses close together, rather than the chemical itself.

In view of the limited experience we will ensure through the central randomisation that at least one individual per intranasal or intramuscular route completes the first safety visit following immunisation before further individuals are enrolled into that group and immunised. The maximum number of participants receiving their first immunisation in the schedule per day per centre will be two.

There is no direct benefit to the volunteers. They will be reimbursed for their time and travel.

This may be a difficult trial to recruit to as the IN and IVAG routes are less usual. SGUL and York are experienced centres, and the CN54gp140 is very stable if immunisations have to continue beyond the anticipated year.

As there is no placebo group, there is a risk that adverse events will be over-reported, but this should not influence grade and is not a concern at this stage of evaluation.

The CN54gp140 has to be reconstituted with adjuvant or gel for each of the immunisation routes. This will be carried out by qualified health care professionals who have had appropriate training, according to standard operating procedures for each clinical centre. The qualifications, training and operating procedures will be approved by a pharmacist with clinical trials experience local to each clinical centre, the Sponsor and MRC CTU as part of the clearance procedures for each clinical centre prior to enrolment. The dispensing records will be checked at each monitoring visit including used vials.

There are challenges in consistently administering vaccine by the IN route. As the number of participants allocated to this group is only 6 we propose that these are only administered at SGUL where the clinical staff have extensive experience. As the regimens utilise a variety of routes, it would only be possible to blind clinical staff and participants to the low and high dose IM. Although this would avoid any risk of bias in adverse event reporting, the investigators have decided not to blind clinical staff so that they can check the vials containing CN54gp140 and the reconstitution with adjuvant. However, the allocation will not be known to the investigator until the participant is in clinic on the day of enrolment. The participants will be blind to the low or high dose IM.

The laboratory endpoints will be analysed in laboratories at Imperial College, and the staff blind to the regimen, although not the timepoint. Every precaution will be taken to ensure

specimens are not lost in transfer from York or SGUL to IC, and aliquots kept behind at York as a back-up should sufficient sample be available.

2.3 Background and rationale for additional boosting

The initial design of the intranasal arm of the MUCOVAC2 study was based on two key criteria. First chitosan had been previously used as a carrier for intranasal immunisation with genetically detoxified diphtheria toxin in humans and was assessed as safe, therefore entry into human trials with this mucosal delivery strategy was seen to have a relatively low bar in terms of potential safety concerns. Second, at the time of designing the study we had recently completed a study in mice that demonstrated the particular ability of chitosan to adjuvant mucosal responses to gp140 when delivered IN. While intranasal immunisation induced similar systemic IgG responses to parenteral immunisation, the most dramatic effect of chitosan was seen on the magnitude of vaginal IgA responses following IN vaccination (not seen with parenteral immunisation). These data supported the relative superiority of the IN route for priming of IgA responses in the vagina in mice. However, subsequent to design of the human clinical protocol the responses to intranasal immunisation using the clinical dose of gp140 and chitosan was assessed in non-human primate studies. Here following an identical protocol to that used in MUCOVAC2 there was minimal responsiveness to intranasal immunisations with very low responses detected in 3 of 12 animals. The MUCOVAC2 study as currently configured will assess whether the observations from non-human primate studies are predictive of human responses, however the working assumption is that they are likely to be the same. Based on this assumption and to optimise potential scientific output of the project it is proposed to assess the impact of additional intramuscular boosts on the profile of systemic and mucosal responses following intranasal priming. In parallel it is proposed to boost a comparative group having received three intramuscular immunisations.

The scientific justification for such an approach is as follows. Previous studies have shown that intranasal immunisation by itself can often induce poor systemic and mucosal responses in non-human primates and humans, but when combined with intramuscular boosting can induce better mucosal and systemic responses than either route of immunisation alone. This has been most clearly demonstrated for HIV-1 gp140 in a macaque study undertaken by Barnett et al [49]. This study compared the utility of single route (IM or IN) to mixed route immunisations.

Assessment of serum antibodies demonstrated that the 3xIM group (with MF59 as adjuvant) induced relatively strong serum antigp140 IgG responses in all animals. In contrast, the 3xIN group (with LTK63 as adjuvant) generally induced low or undetectable serum antibody responses. These data reflect what we have observed in our macaque study with 3xIN immunisation together with chitosan, and perhaps what we will see in the human clinical trial. However, in the Barnett study, the 3xIN/2xIM group induced serum anti-gp140 IgG titers that were significantly higher than that seen with 5xIM.

Assessment of mucosal (vaginal, nasal and saliva) antibody responses by Barnett et al demonstrated that IN/IM immunisations induced significantly higher IgG responses in vaginal washes in comparison with all other immunisation groups. Interestingly, IgA responses in vaginal washes were significantly higher in the macaques immunised IN/IM compared with the macaques immunised IM/IN or intranasally, but not in the macaques immunised intramuscularly. Serum IgA responses were significantly higher in the macaques immunised IN/IM in comparison with all other groups. These data strongly argue that additional intramuscular boosting of the intranasal group in the MUCOVAC2 trial should be assessed and may result in superior mucosal responses.

Boost groups

The boost phase will aim to re-consent and enrol up to ten volunteers having received either three intranasal immunizations (IN) (five subjects) to receive two additional boosting

immunisations by intramuscular injection (3xIN + 2xIM) or three intramuscular immunisations (IM) (five subjects) to receive two additional boosting immunisations by intramuscular injection (3xIM + 2xIM). The spacing between the priming phase (3x immunisations) and the boosting phase is not critical but should be 3-6 months from the initial three immunisations. However the spacing between the two boosting immunisations should be exactly 1 month.

3 SELECTION OF CENTRES/CLINICIANS

The principal investigators will be from St. George's University London and University of York. Volunteers will be seen by clinical staff from Vaccine Research Institute, St George's and Hull York Medical School Experimental Medicine Unit (HYMS EMU), York Hospital who have experience of Phase 1 clinical trials.

4 SELECTION OF PARTICIPANTS

4.1 Participant inclusion criteria

1. women aged between 18 and 45 years on the day of screening
2. available for follow-up for the duration of the study (6 months from screening)
3. willing and able to give written informed consent
4. at low risk of HIV and willing to remain so for the duration of the study defined as:
    - no history of injecting drug use in the previous ten years
    - no gonorrhoea or syphilis in the last six months
    - no high risk partner (e.g. injecting drug use, HIV positive partner) either currently or within the past six months
    - no unprotected anal intercourse in the last six months, outside a relationship with a regular partner known to be HIV negative
    - no unprotected vaginal intercourse in the last six months outside a relationship with a regular known/presumed HIV negative partner
5. willing to undergo a HIV test
6. willing to undergo a vaginal examination and genital infection screen
7. Normal cervical smear within 12 months of screening
8. if heterosexually active female, using an effective method of contraception with partner (combined oral contraceptive pill; injectable or implanted contraceptive; any IUCD/IUS; consistent record with condoms if using these; physiological or anatomical sterility in self or partner) from 14 days prior to the first vaccination until 4 months after the last, and willing to undergo urine pregnancy tests prior to each vaccination
9. agree, should they be allocated to IVAG immunisations, to abstain from having a bath or shower for 4 hrs after each dose, and from sex for 24 hrs after each dose, and to use condoms (without spermicide) for one week after each dose
10. agree, should they be allocated to IVAG or IN immunisations to abstain from medications or other agents that are applied via the same route from 24 hrs prior to dosing through to the safety assessment 4 weeks later
11. agree to abstain from donating blood for three months after the end of their participation in the trial, or longer if necessary
12. registered with a GP for at least the past three months
13. satisfactory response received from GP before randomisation

For participants proceeding to boost phase:

14. satisfactory completion of the priming vaccinations and followup in the main study
15. willing to continue in the study for at least a further 12 weeks with 5-6 extra visits

4.2 Participant exclusion criteria

1. pregnant or lactating
2. clinically relevant abnormality on history or examination including
    - history of grand-mal epilepsy
    - cranial nerve palsies
    - severe eczema
    - severe epistaxis
    - liver disease with inadequate hepatic function
    - haematological, metabolic, gastrointestinal or cardio-pulmonary disorders
    - uncontrolled infection; autoimmune disease, immunodeficiency or use of immunosuppressives in preceding 3 months

- using inhaled cortico-steroids and IN medications

3. known or suspected history of clinically relevant cervico-vaginal disease, malignancy or abnormality, which in the opinion of the investigator might interfere with IVAG dose administration
4. known or suspected history of clinically relevant nasal surgery, injury or condition likely to require regular intranasal medication, which in the opinion of the investigator might interfere with IN dose administration
5. known hypersensitivity to any component of the vaccine formulations used in this trial, or a seafood allergy or have severe or multiple allergies to drugs or pharmaceutical agents
6. history of severe local or general reaction to vaccination defined as
    a. **local**: extensive, indurated redness and swelling involving most of the antero-lateral thigh or the major circumference of the arm, not resolving within 72 hours
    b. **general**: fever >= 39.5^{o}C within 48 hours; anaphylaxis; bronchospasm; laryngeal oedema; collapse; convulsions or encephalopathy within 72 hours
7. receipt of live attenuated vaccine within 60 days or other vaccine within 14 days of enrolment
8. receipt of an experimental vaccine containing HIV envelope proteins at any time in the past
9. receipt of blood products or immunoglobin within 4 months of screening
10. participation in another trial of a medicinal product, completed less than 30 days prior to enrolment
11. HIV 1/2 positive or indeterminate on screening
12. positive for hepatitis B surface antigen, hepatitis C antibody or serology indicating active syphilis requiring treatment
13. grade 1 or above routine laboratory parameters (see appendix 4 for definitions). *Hyperbilirubinemia to be considered an exclusion criterion only when confirmed to be conjugated bilirubinemia*
14. unable to read and speak English to a fluency level adequate for the full comprehension of procedures required in participation and consent.
15. unlikely to comply with protocol

4.3 Number and source of participants

Healthy female volunteers will be recruited through advertising and given a telephone number to contact. They will be provided with further information about the study and asked to complete a short interview (by telephone or in person) to assess their suitability. At this point they will be allocated a number from the screening register. They will be given or sent an information sheet (appendix 1).

The target enrolment is 36 women, and the intention is to enrol equally between the two clinical centres. In the event that one centre is initiated in considerable advance of the second centre, or enrols more quickly than expected, then modifications can be made in the interests of completing the trial in a timely manner. The decision to modify the target enrolment will be proposed by the Trial Management Group (section 16.1) to the Sponsor for approval.

Up to 10 participants (5 from IN group and 5 from IM 100µg group) approaching or having reached visit 10 will be invited to be boosted with 2 further IM 100µg vaccinations 4 weeks apart with the first 12-24 weeks after last priming vaccination. They will be provided with an information sheet and asked to sign a consent form.

4.4 Screening procedures and pre-randomisation investigations

At the screening visit the trial will be discussed in detail, and a check of eligibility conducted using a case report form to standardise this procedure. Any questions about the study will be answered. If volunteers are still willing and interested they will be asked to sign the informed consent form (appendix 2).

To ensure informed consent, volunteers will go through the following processes in detail with a member of the study team

1) Pre-HIV risk assessment & discussion
2) Safe sex counselling
3) That it is unknown whether or not the study vaccines will protect against HIV infection
4) That following immunisation they may develop antibodies that will produce a positive reaction in a routine HIV test, but that provisions have been made to distinguish between a post vaccination response and HIV infection during and after the trial
5) The level of care that will be made available to them should they be found to be HIV infected at any time during their participation in the study, including the screening period
6) That they, or their partner should continue to use a reliable form of contraception for 14 days prior to the immunisation period and for 4 months afterwards
7) That they should continue to use condoms with sexual partners whose HIV status is not known
8) That they may be subject to social risk if they develop HIV antibodies, or by revealing their participation in the study

After informed consent has been collected, assessments and investigations will be undertaken according to the schedule in section 7, table 3. These include demographic, sexual and medical histories, general and genital examinations, and collection of urine and blood samples for routine laboratory investigations. Screening investigations for sexually transmitted infections including HIV will be collected.

See section 7.2

As soon as the medical report is received from the volunteer’s GP and all required test results are available:

- data will be entered onto the screening CRFs and the results of the screening investigations will be reviewed and eligibility signed off by a physician;
- completed screening CRFs for all volunteers, whether or not they are eligible will be faxed promptly to the MRC CTU for entry onto the trial database.

4.5 Screening procedures and investigations for boosting phase

It is envisaged that most participants for the boosting substudy will enter at visit 10 or soon after and as this is final visit of the main study they will have had a full laboratory and clinical assessment done.

If more than six weeks has elapsed since visit 10 a full rescreen is not required but there should be a risk assessment and biochemistry and haematology bloods should be repeated but there is no need for repeat GP letter or smear test. There is no need for repeat sexual health screen (HIV, hepatitis B&C, gonorrhoea or Chlamydia tests) unless a history of a new partner or other potential risk factor.

5 RANDOMISATION & ENROLMENT PROCEDURE

5.1 Randomisation practicalities

The enrolment visit will take place within 6 weeks (42 days) of the screening visit and will be the same day as the first dosing visit. Assessments and procedures will be undertaken according to the schedules (section 7 and table 3), and data entered on the case report forms.

Volunteers who are eligible and willing will be randomised by completing the appropriate case report form (Form 2) and contacting the MRC CTU with the participant present. Eligibility will be checked at CTU, the trial number confirmed and regimen allocated.

The rate of enrolment will be controlled centrally.

- The first participant enrolled in each centre will complete the safety visit following the first IM rgp140 100μcg (York) and the first IN rgp140 100μcg (St George's) before any other individuals are randomised in that centre
- This gap between the first individual enrolled and the subsequent cohort in each centre will remain in the trial schedule for the second and third immunisations, providing sufficient time to interrupt further immunisations in the unlikely event that this is necessary

In the event of multiple grade 2 or above adverse reactions in any of these early enrolments, there will be a hold on further immunisations until an urgent TMG review to determine whether an unscheduled review by the Independent Data Monitoring Committee is required.

Both sites will be notified once the first randomisation has taken place and kept informed of ongoing randomisations.

Further details on the process of randomisation can be found in section 10.1.

RANDOMISATIONS

Tel: 020 7670 4783 Mon - Fri, 08:30 – 17:00)
Fax: 020 7670 4659

5.2 Randomisation codes and unblinding

The randomisation list linking subject numbers to treatment will be generated by Medical Research Council Clinical Trials Unit (MRC CTU), London, UK and copies held by the designated member of staff responsible for the inventory of products on delivery to each clinical centre.

The trial is open-label but the laboratory staff will be blind to the regimen throughout and the participants will be blind to the dose administered in the intramuscular regimen.

5.3 Co-enrolment guidelines

Participants will be advised that they cannot enrol in any other trials during the period from screening to the final visit in MUCOVAC2. All participants will be entered onto the TOPS database as a measure to prevent over- volunteering.

If staff discover that a participant is enrolled on another study during this period, they must immediately contact the Principal Investigator for the clinical centre, or the Medical Expert

for MRC CTU for advice. Decisions will be reviewed on a case by case basis, and participant safety will be the primary concern.

6 TREATMENT OF PARTICIPANTS

6.1 Vaccine products

Thirty-six participants will receive CN54gp140 vaccine in a schedule of dose, formulations and routes as described below. Each participant will receive three immunisations, four weeks apart.

CN54gp140 aqueous solution will be manufactured by Polymun Scientific, Vienna, Austria.

6.1.1 Products and administration

IM vaccine (group 1,2)

IM immunisations will contain 20 or 100µg CN54gp140 and 5µg GLA (Infectious Disease Research Institute, Seattle, USA).

The dose volume will be 0.4ml injected into the deltoid muscle of the upper arm, using a 21–25 gauge needle long enough to reach deep into the muscle. The needle will be inserted at an angle of approximately 90° to the skin. Participants receiving IM immunisations will be asked at each immunisation which arm they would like to be injected.

IN vaccine (group 3)

IN immunisations will contain 100µg CN54gp140 in an aqueous solution containing 0.5% (w/v) chitosan glutamate (Protasan UP G 213; FMC BioPolymer AS/NovaMatrix, Sandvika, Norway). The dose volume will be 0.4ml – approximately 0.2ml per nostril.

Participants will lie or sit with the head tilted back such that gravity pulls the administered dose into the nostrils towards the nasal cavity. The participants must be asked to breathe through their mouths while approximately equal volumes of dose are dropped into both nostrils, at a rate of approximately one drop per nostril every 5 sec. The participant should remain with the head tilted back for 5 minutes after completion of dosing, and must not blow her nose until 1 hour after dosing.

IVAG vaccine (group 4)

IVAG immunisations will contain 500µg CN54gp140 in an aqueous gel vehicle (Gel#2734; Particle Sciences Inc, Bethlehem, USA). Gel#2734 comprises common pharmaceutical excipients, used in many products for topical, IVAG use: carbopol (gelling agent); benzyl alcohol (preservative); sodium hydroxide (pH adjuster); and water. The dose volume will be 3.0ml.

The dose will be extruded into the vaginal vault according to the manufacturer's instructions: an applicator is simply inserted well into the vagina and the plunger is depressed to extrude the vaccine. Participants may self-administer if they wish.

Boosts: -IM vaccine

IM immunisations will contain 100µg CN54gp140 and 5µg GLA (Infectious Disease Research Institute, Seattle, USA).

The dose volume will be 0.4ml injected into the deltoid muscle of the upper arm, using a 21–25 gauge needle long enough to reach deep into the muscle. The needle will be inserted at an angle of approximately 90° to the skin. Participants receiving IM immunisations will be asked at each immunisation which arm they would like to be injected.

6.1.2 Accountability for used and unused supplies

The PIs must ensure that all IMP supplies are kept in a secure area accessible only to authorised individuals, and maintained in storage that guarantees the following temperatures:

- CN54gp140 protein at below –15°C
- GLA-AF at 2-8°C
- 1% chitosan glutamate at below –15°C
- Gel#2734 at room temperature

Upon receipt of supplies, a designated member of staff will conduct an inventory and acknowledge receipt to the supplier.

A record must be kept of all CN54gp140, GLA-AF, 1% chitosan glutamate and Gel#2734 vehicle used during the trial. This will include the description (lot numbers and expiry dates) and quantity of IMP received at the trial site and date of receipt, as well as a record of when (date of administration) and to whom (subject number) it was dispensed.

At the end of the trial, IMP accountability will be checked by the designated member of staff responsible for the inventory and trial monitors. The Sponsor and the PIs will retain copies of the complete IMP accountability records.

All supplies (used and unused) will be retained at the trial sites until the Sponsor gives instructions for their return/destruction.

6.1.3 Vaccine reconstitution

The CN54gp140 has to be reconstituted with adjuvant or gel for each of the immunisation routes. This will be carried out by qualified health care professionals who have had appropriate training, according to standard operating procedures for each clinical centre, which are summarised below.

The vials containing the mixed formulation may be stored at room temperature during the period between mixing and administration. Although stable at 24 hours, Investigators will be encouraged to administer the vaccine as soon as possible after mixing, and not beyond 8 hours.

The date and time of administration will be recorded in the CRF.

IM doses

The CN54gp140 will be supplied at 0.53 mg/mL and 0.11mg/mL, and the GLA at 25 µg/mL (0.5mL/vial).

For the 100 µg CN54gp140 dose, reconstitution will involve gentle mixing of 0.35 mL of 0.53 mg/mL CN54gp140 solution with 0.35 mL GLA-AF, and drawing up of 0.4 mL of this mixture into a 0.5–1 mL syringe, for administration.

For the 20 µg CN54gp140 dose, reconstitution will involve gentle mixing of 0.35 mL of 0.11 mg/mL CN54gp140 solution with 0.35 mL GLA-AF, and drawing up of 0.4 mL of this mixture into a 0.5–1 mL syringe, for administration.

IN doses

The CN54gp140 will be supplied at 0.53 mg/mL, and the chitosan glutamate as a 1% (w/v) aqueous solution (0.5mL/vial).

Reconstitution will be achieved by gentle mixing of 0.35 mL of 0.53 mg/mL CN54gp140 solution with 0.35 mL 1% chitosan solution. Immediately before dosing, 0.4 mL will be drawn into a 0.5–1 mL syringe for administration.

IVAG doses

The CN54gp140 will be supplied at 0.53 mg/mL (1.3mL/vial), and the Gel#2734 in pre-filled 5ml syringes (4mL/syringe).

Reconstitution will be achieved by mixing 1.1 mL of 0.53 mg/mL CN54gp140 solution with 2.4 mL Gel#2734, by passing the mixture 20 times between two connected 5 mL syringes. Immediately before dosing the mixture, contained in one of the two syringes used to mix,

will be transferred into an ORTHO® vaginal applicator (Janssen-Cilag) for administration. Due to predictable losses during the mixing procedure and the transfer to the ORTHO applicator, and because a predictable proportion of the mixture remains in the applicator after administration, the dose volume administered is 3.0 mL.

6.1.4 Immunisation schedule

The doses, formulation, routes and schedule of immunisation are described in table 1 below.

Table 1 Schedule of doses, formulation and routes of immunisation

Group	Route of immunisation; dose of CN54gp140			Total cumulative dose of CN54gp140
	Dose 1 at wk0	Dose 2 at wk4	Dose 3 at wk8	
1<br>N=10	0.4ml IM<br>20µg CN54gp140<br>5µg GLA	0.4ml IM<br>20µg CN54gp140<br>5µg GLA	0.4ml IM<br>20µg CN54gp140<br>5µg GLA	60µg CN54gp140
2<br>N=10	0.4ml IM<br>100µg CN54gp140<br>5µg GLA	0.4ml IM<br>100µg CN54gp140<br>5µg GLA	0.4ml IM<br>100µg CN54gp140<br>5µg GLA	300µg CN54gp140
3<br>N=6	0.4ml IN<br>100µg CN54gp140<br>Chitosan 0.5%	0.4ml IN<br>100µg CN54gp140<br>Chitosan 0.5%	0.4ml IN<br>100µg CN54gp140<br>Chitosan 0.5%	300µg CN54gp140
4<br>N=10	0.4ml IM<br>100µg CN54gp140<br>5µg GLA	3ml IVAG<br>500µg CN54gp140<br>Gel #2734	3ml IVAG<br>500µg CN54gp140<br>Gel #2734	1100µg CN54gp140

Table 1a – boost schedule (dose 4 & 5 should be 4 weeks apart)

Group	Route of immunisation; dose of CN54gp140		Total cumulative dose of CN54gp140
	Dose 4 at wk20-32	Dose 5 at wk24-36	
2<br>N=5	0.4ml IM<br>100µg CN54gp140<br>5µg GLA	0.4ml IM<br>100µg CN54gp140<br>5µg GLA	500µg CN54gp140
3<br>N=5	0.4ml IM<br>100µg CN54gp140<br>5µg GLA	0.4ml IM<br>100µg CN54gp140<br>5µg GLA	500µg CN54gp140

6.1.5 Compliance and adherence

All IM and IN immunisations will be administered by site staff to ensure compliance. IVAG immunisations may be self-administered by participants at the trial sites, but applicators will be checked by staff after each administration. If staff believe an incomplete dose was given at any immunisation, for instance if a participant sneezes during IN administration, this will be recorded together with the reason in the CRF.

6.1.6 Dose modifications and discontinuation

There are no planned modifications to dose, other than discontinuation.

The schedule may be modified if a participant has symptoms or signs on the day of scheduled immunisation, and the investigator considers it best to defer the immunisation. The participant will be asked to return for review within the window period of the scheduled

immunisations (-3/+3 days from week 4 and 8 for the second and third immunisations respectively). Examples of the type of symptoms and signs are given in section 7 table 2.

An investigator may decide to permanently discontinue dosing in a participant who has received one or two immunisations, if the investigator deems that continuing might compromise participant wellbeing or interfere with the achievement of the trial's objectives. Such a decision should only be taken in consultation with the local Principal Investigator and CTU Medical Expert. Participants will be encouraged to continue to attend trial visits for sampling and safety monitoring.

Discontinuation is recommended in the event of a grade 3 or 4 clinical or laboratory event (confirmed on examination or repeat testing respectively) which is considered possibly, probably or definitely related and which did not resolve within 72 hours.

Dosing must be discontinued in participants who become pregnant.

Participants may decide to discontinue dosing. They will be encouraged to provide a reason, and to remain in follow-up. If the participant explicitly states their wish not to contribute further data to the study, the MRC CTU should be informed in writing.

MRC CTU should be informed as soon as possible about any possible discontinuation of dosing or withdrawal from the trial, and within 1 working day of a decision being taken to discontinue when that decision is informed by an adverse event (section 8.2).

6.2 Clinical management of adverse events

Events will be managed by the clinical trial team who will assess and treat the event as appropriate, including referral to an independent physician and/or the participant's General Practitioner if required. There will be clinical operating procedures in place for the management of abnormalities detected following urinalysis or routine laboratory tests.

See also section 7.4

6.3 Non-trial treatment

As stated in exclusion criteria 7, 8, 9 and 10 in Section 4.2, participants should not have received other immunisations, blood products, immunoglobulin and other trial medication within specified periods prior to enrolment. This applies during the trial follow-up through to the final visit 20 weeks from enrolment, 12 weeks after the last scheduled immunisation, unless the treatment is required for an emergency.

Should a participant require immunisation for the purposes of travel or occupation during the trial, the request will be reviewed by the Trial Management Group who will advise on timing and whether or not the trial immunisation schedule needs to be amended.

As stated in inclusion criterion 9, participants should be willing to abstain from IN or IVAG medications and agents applied via the same routes from the time of the IN/IVAG immunisation through to the safety assessment scheduled 4 weeks later. If participants experience congestion following immunisation, or independent of this as part of an allergic rhinitis or hayfever, this will be managed with oral anti-histamines wherever possible. Should the participant require inhaled corticosteroids, further immunisations via the nasal route will be discontinued.

Participants will be allowed to continue with hormonal contraception if this forms part of their regular appropriate contraception plan. The precise method will be recorded on the screening CRF and any changes on the medication CRF.

All concomitant medication will be recorded in CRF, including any dispensed by the investigators in the management of adverse events or reactions.

6.4 Issues related to HIV

Only volunteers with a negative 4th generation HIV antibody/antigen result will be enrolled onto the study.

A 4th generation HIV antibody/antigen assay, the standard laboratory method for diagnosing HIV infection, will be used to screen volunteers after they have received appropriate counselling. An HIV test will be performed at two time-points in the study: the screening visit, and the final visit 20 weeks from enrolment.

At each immunisation visit a risk assessment will be conducted and an additional HIV test will be performed if there has been a change in risk status. Participants will be counselled by study personnel about the importance of condoms and reminded on the day of each immunisation. Hypo-allergenic condoms will be provided free of charge.

In the event that the blood specimen collected at the final visit is positive, participants will be provided with appropriate certification of their HIV status, and invited to return to the clinical centre on an annual basis for re-testing until such time as the test becomes negative.

6.4.1 Verification of HIV status of participants

In the event of an equivocal or positive result, a specimen will be processed through a range of assays according to the local laboratory operating procedures to establish the HIV status of the individuals. A confirmatory specimen will be collected at a later date, if the first result suggests that the participant is HIV infected.

If a participant requires certification independent of the local laboratory, then this can be arranged through the MRC Clinical Trials Unit.

6.4.2 HIV infection

In the unexpected circumstances that a participant in the trial acquires HIV infection, they will be referred for clinical care and counselling.

6.4.2.1 Referral for clinical care

Participants will be referred initially to a specialist physician for a full discussion of the clinical aspects of HIV infection. Further investigations will be undertaken as necessary. Should the participant prefer to be managed at a hospital closer to their home, this will be arranged.

6.4.2.2 Referral for counselling:

This will be arranged by the specialist physician, to a counsellor at their clinical centre.

The counselling process will assist the participant in the following issues:

- psychological and social implications of HIV infection
- who to inform and what to say
- implications for sexual partners
- avoidance of risk to others in future

7 ASSESSMENTS AND FOLLOW-UP

7.1 Duration of follow-up and schedule

The assessments that will be performed at each visit are described in Table 3 (and for those receiving boosts in table 3a).

Participants will be required to make a minimum of ten scheduled outpatient visits over the course of 26 weeks (182 days). Time 0 will start on the day of enrolment, which is also the day of randomisation and first immunisation. There will be five or six further visits over ~12 weeks for those on the boost schedule.

Screening can take place up to 6 weeks (42 days) before time 0.

Immunisations will take place at weeks 0, 4 and 8 (and visits 10/11 and 13 for those receiving boosts).

Serum will be collected for immunogenicity at screening, baseline, and weeks 4, 5, 8, 12 (the primary endpoint), 16 and 20. Vaginal samples will be collected at screening, baseline, and weeks 4, 5, 8, 12 (the primary endpoint), 16 and 20. Cervical samples will be collected at screening and weeks 5 and 12. Peripheral blood mononuclear cells (PBMC) will be collected at baseline, and weeks 1, 4, 5, 8, 9, 12 and 20. For those receiving boosts details of specimens to be taken are given in table 3a.

Adverse events will be assessed during the enrolment visit following immunisation and at every visit thereafter. Routine laboratory safety parameters will be collected at screening, and weeks 1, 5, 9 and 20 (and visits 12 and 14 for those receiving boosts).

Additional visits

Additional visits and assessments may be required to evaluate an adverse event, and/or identify a diagnosis. These are compatible with the protocol.

Referral to an independent specialist with the appropriate expertise will be arranged if there is uncertainty about the relationship to vaccine.

Visit windows

The second and third immunisation visits scheduled for weeks 4 and 8 will be compliant with the protocol if they take place ±3 days either side of the target date determined by the date of enrolment. If there is a delay >4 weeks a decision will be made by the TMG as to whether immunisation will continue.

The post-immunisation safety visits scheduled for weeks 1, 5 and 9 will be compliant with the protocol if they take place -5 to +3 days of the target date.

The primary endpoint and other follow-up visits scheduled for weeks 12, 16 and 20 will be compliant with the protocol if they take place ±7 days either side of the target date.

7.2 Assessments at Screening

7.2.1 Demographics, medical history and examination

Demographic information such as age, ethnic origin, smoking, alcohol and illicit drug history will be collected at screening and entered onto the screening CRF.

A past and current medical and gynaecological history will be collected at screening during a face to face structured interview using a case report form, including details of any previous reaction to vaccination, allergies, history of epileptic fit, reproductive and respiratory symptoms and contraceptive practices (current method and the length of time using the method). Information on the menstrual cycle (start of last menses and typical duration and cycle) and concomitant medications will be collected.

The general examination will include weight (kg), height (cm), temperature and arm circumference, blood pressure, inspection of the skin to exclude severe eczema, inspection of the nasal, upper respiratory passages and ears, respiratory, cardio-vascular, abdominal, and genital examination with a speculum in order to inspect the vagina and cervix. An assessment of cervical and axillary lymph nodes will also be undertaken.

7.2.2 Sexual history and genital infection screen

A sexual history will be taken for the preceding 6 month period, and participants will be asked whether they have ever been tested for sexually transmitted infections in the past and if so, whether any were found.

The following will be collected in all participants:

- serology for syphilis (ELISA), HBV (hepatitis B surface antigen), HCV (hepatitis C antibody) and HIV-1/HIV-2 (4th generation HIV antibody/antigen test)
- endo-cervical samples for *Neisseria gonorrhoea* and *Chlamydia trachomatis*
- vaginal samples for *Trichomonas vaginalis*, *candida albicans* and bacterial vaginosis

7.2.3 Routine laboratory parameters

Peripheral blood will be collected by clinical staff experienced in phlebotomy into the appropriate containers and transported to the local laboratory.

Mid-stream urine will be collected into a sterile container and either tested on site or transported to the local laboratory.

At the St George's trial site all laboratory assessments, except urine dipstick and pregnancy tests which will be done at site, will be performed by The Doctors Laboratory, London, UK.

At the York trial site all laboratory assessments will be performed by the York Hospital Pathology Laboratories, except for urine dipstick, pregnancy tests and microscopy for *Trichomonas vaginalis, Candida albicans* and bacterial vaginosis which will be performed by HYMS EMU. Should confirmatory HIV testing be required, this will be performed at the Leeds Teaching Hospitals NHS Trust Virology Laboratory.

The following parameters will be collected in all participants:

- Hb, total WBC, neutrophils, lymphocytes and platelets
- creatinine, total bilirubin, alkaline phosphatase, AST, ALT and glucose
- urinalysis using a dipstick for protein, ketones, blood, leukocyte esterase, and nitrites
- urine test to exclude pregnancy

A urine specimen will be sent to the laboratory if the level of protein, blood, leukocyte esterase or nitrites is considered clinically relevant.

7.3 Procedures for assessing immunogenicity

Samples will be collected at the timepoints specified in Table 3 (and table 3a for boosts) and transferred to the appropriate Immunology Core Laboratory for analysis. Detailed instructions for collection and processing of samples at trial sites will be supplied by the Sponsor in the form of a technical manual.

For measurement of **anti-CN54gp140 antibodies in serum**, 7ml peripheral venous blood will be collected into a serum separator tube (SST, gold top) or plain blood collection tube, and then processed to obtain serum.

For the measurement of **anti-CN54gp140 antibodies in cervical and vaginal secretions**, two Weck-Cel® ophthalmic sponges (Medtronic) will each be placed in the cervical os for 1 minute to absorb secretions, and then a further two Weck-Cel® sponges

placed in the vaginal fornices for 1 minute. After 1 minute the sponges are immediately transferred into Costar® Spin-X® polypropylene microcentrifuge filter tubes (Corning Inc) containing extraction buffer. The samples will then be processed. Sampling will take place during speculum examination at screening and weeks 5 and 12

For measurement of **anti-CN54gp140 antibodies in vaginal secretions**, participants will use an INSTEAD Softcup™ (Evofem Inc) to self-sample (unless they are using IUCD). Softcup is a commercially available alternative to tampons which comprises a flexible plastic ring from which hangs a very thin plastic 'cup'. The device is inserted into the vagina and secretions collect in the cup. The Softcup is then removed for sample processing. Participants will be trained by site staff to use the Softcup, which on each sampling occasion must be inserted into the vagina for at least 1 hour. For those participants with an IUCD in situ 2 Weck-Cel sponges will be inserted into the vagina using sponge forceps without the need for a speculum examination and held there for one minute by a health care professional.

For measurement of **B-cell** and **T-cell responses**, 30-35ml of peripheral venous blood will be collected into tubes containing sodium heparin as an anti-coagulant, mixed by inverting gently several times and then processed to obtain PBMC.

7.4 Procedures for assessing safety

7.4.1 Adverse event assessment

Information on adverse events will be collected through open questions at every visit from and including the enrolment visit as indicated in Table 3 (and 3a). Examples of an open question are 'have you had any problems or seen a doctor since your last visit?' and 'have you taken any medicines since your last visit, and if so, what for?'

The investigator will record the diagnosis or the symptoms if a diagnosis is not apparent, the date of onset and the date of resolution if appropriate. The severity will be determined according to the CTCAE table version 4.02
http://www.acrin.org/Portals/0/Administration/Regulatory/CTCAE_4.02_2009-09-15_QuickReference_5x7.pdf

The relationship to vaccine will be determined by the investigator according to the definitions provided in section 8. All of this information will be recorded in the adverse event CRF.

7.4.2 General examination and vital signs

A general examination, and assessment of vital signs as described in section 7.2.1, will be performed at time points specified in tables 3 and 3a, and at additional time points if indicated according to the answers given during the adverse event assessment.

7.4.3 Routine laboratory parameters, urinalysis and vaginal flora

A urine specimen will be collected for a urinary pregnancy test prior to each immunisation, and at the final visit.

Peripheral blood and urine will be collected and analysed, as described in 7.2.1, for the following parameters at the time points specified in Table 3 and 3a, and at additional time points if indicated to further evaluate or follow up adverse events:

Blood

- Creatinine, AST, ALT, alkaline phosphatase, total bilirubin, glucose
- Hb, total WCC, neutrophils, lymphocytes, platelets.

Urine

- protein, ketones, blood, leukocyte esterase, and nitrites

A sample of vaginal secretions will be taken and smeared onto a slide at weeks 5 and 12. These will be gram stained and analysed at the end of the study for evidence of any alteration of vaginal flora compared to baseline.

7.4.4 Solicited local and systemic clinical and laboratory adverse events

Various local and systemic adverse events are known to be associated with licensed vaccines, and are referred to as '**solicited adverse events'**. These include disturbances in routine laboratory parameters, and are described in table 2 below.

Information on solicited adverse events will be collected on the day and 3-10 days following each immunisation through direct questions asked during a structured face to face interview and through examination according to the schedule. This information will be recorded directly into the CRF.

In addition systemic laboratory adverse events will be collected through routine laboratory testing according to the schedule. These will be recorded on the standard laboratory reports and may be transcribed into the CRF or a copy of the laboratory report attached to the CRF.

Participants will also be asked to complete a diary card recording solicited adverse events relevant to each of the three routes that start within 7 days of each immunisation, regardless of the route of immunisation they receive.

Table 2 Solicited adverse events

Route	Intramuscular	Intranasal	Intravaginal
Local AEs<br>(immunisation site)	Discomfort<br>Redness<br>Swelling (soft)<br>Induration (hard)<br>Blisters	Discomfort<br>Congestion<br>Discharge<br>Bleed	Discomfort (itching, burning)<br>Pain during sexual intercourse<br>Abnormal (unusual) discharge<br>Non-menstrual bleeding<br>Redness[1]<br>Swelling[1]<br>Sores/ulcers[1]
Systemic Clinical AEs	Temperature<br>Chills<br>Myalgia/flu-like general muscle aches<br>Malaise (excess fatigue)<br>Headache<br>Nausea		
Systemic Laboratory AEs	Creatinine, AST, ALT, alkaline phosphatase, total bilirubin, glucose<br>Hb, total WCC, neutrophils, lymphocytes, platelets		

[1]these solicited adverse events can only be captured during a speculum examination

The events will be **graded** according to the toxicity table in appendix 4.

Relationship will be recorded in the immunisation CRF but not on the diary card, on the assumption that any of these events starting within 7 days of an immunisation are at least possibly related. It will also be assumed that nasal and vaginal symptoms recorded in the diary card are unlikely to be related to IVAG/IM and IN/IM immunisations respectively. If the onset is beyond 7 days, the event will be recorded on the adverse event CRF and a relationship determined by the investigator reviewing the event.

7.4.5 Procedure for follow-up of adverse events and pregnancy

The Investigator will make every effort to monitor all adverse events, regardless of severity, until resolution or stabilisation, and to obtain documentary evidence of the outcome of pregnancy, in order to report this on the CRF during the trial.

After the database is locked and the trial is closed, any additional information about adverse events or pregnancy that comes to the attention of the investigator should be reported by email to the Medical Expert at MRC CTU and the Chief Investigator on behalf of the Sponsor.

7.4.6 Serious adverse events

A serious adverse event is defined in section 8.

All serious adverse events should be reviewed by the Principal Investigator for the clinical centre, and discussed at the next Trial Management Group call (section 16.1).

A serious adverse event might cause a PI to consider discontinuing dosing or withdrawing a participant from the trial.

For all serious adverse events, the investigator will complete a serious adverse event CRF and report it to MRC CTU within 1 working day of learning of the event, as described in section 8.

7.5 Criteria for stopping treatment groups or whole trial

In the event of a SUSAR, MRC CTU will organise the expert multi-disciplinary review within 3 working days, as described in section 8.2.

Further immunisations via that route will be held until the review is completed.

A component of the expert review will be to consider whether or not further immunisations by that route or for all routes of administration should be discontinued, and to make a recommendation.

If 3 or more participants experience an unexplained, unexpected grade 3 or 4 clinical or laboratory event (confirmed on attendance or repeat testing) not resolved within 72 hours and considered possibly, probably or definitely related to vaccine product, the Trial Management Group will determine whether or not to call an unscheduled meeting of the Independent Data Monitoring Committee to review the safety data, and whether or not to hold further immunisations until this has taken place. If an unscheduled IDMC is warranted, the Sponsor will be informed and the IDMC asked to make a recommendation to the Chief Investigator and the Sponsor about continuing further immunisations.

Recommendations from the expert panel of Independent Data Monitoring Committee will be made to the Chief Investigator and Sponsor. The decision to stop further immunisations by that route or for all routes of administration rests with the Chief Investigator and Sponsor.

The Sponsor reserves the right to stop the whole trial at any time.

7.6 Procedures at the end of the trial

The trial will be closed when all participants have made their final follow-up visit (visit 10 or visit 15/16 for those on the boost schedule), the data entered into the database and the database locked. There will be a final monitoring/closeout visit to the clinical sites between the last visit and the database lock.

Table 3: Schedule of visits, immunisations and assessments [X means mandatory, (X) means if clinically indicated]

**Visit number**	**1**	**2**	**3**	**4**	**5**	**6**	**7**	**8**	**9**	**10***
**Nature of visit**	**Screening visit**	**Dosing visit**	**Safety visit**	**Dosing visit**	**Safety visit**	**Dosing visit**	**Safety visit**	**Follow-up visit**	**Follow-up visit**	**Follow-up visit**
**Week**	**-6**	**0**	**1**	**4**	**5**	**8**	**9**	**12**	**16**	**20**
**Visit window (days)**	**N/A**	**N/A**	**-5d+3d**	**-3d+3d**	**-5d+3d**	**-3d+3d**	**-5d+3d**	**-7d+7d**	**-7d+7d**	**-7d+7d**
Informed consent	X									
Medical history and demographics	X									
General examination and vital signs	X	(X)	(X)	(X)	X	(X)	(X)	X		(X)
Inspection of administration site[1]	X	(X)	(X)	(X)	X	(X)	(X)	X		(X)
Adverse event assessment		X	X	X	X	X	X	X	X	X
Urine pregnancy test	X	X		X		X				X
Routine laboratory parameters[2]	X		X	(X)	X	(X)	X	(X)	(X)	X
Urinalysis	X		X		X		X			X
HIV test[3]	X	(X)		(X)		(X)				X
HBV, HCV and syphilis tests	X									
Screening for genital infections	X									
Vaginal secretions- Flora analysis	X				X			X		
Blood for serum – immunogenicity	X	X		X	X	X		X	X	X
Blood for PBMC – immunogenicity		X	X	X	X	X	X	X		X
Vaginal secretions – Instead cup[5]	X	X		X	X	X		X	X	X
Cervical & vaginal secretions –Weck-Cels	X				X			X		
**Immunisation[4]**		**X**		**X**		**X**				
Diary card										

[1]Inspection of the administration site will involve an inspection of the skin overlying the muscle of entry, visual inspection of the nasal passages, or a speculum examination of the cervico-vaginal tissues. Inspection will be carried out if indicated according to the clinical symptoms using common operating procedures.

[2]The parameters are detailed in section 7.2.3

[3] At each dosing visit clinic staff will determine whether the risk status for HIV has changed and repeat the test if necessary

[4] Immunisation should be after completion of the other procedures scheduled for that visit except for the AE assessment which will be conducted before and after each Immunisation

[5] Unless the participant has an IUCD in situ when vaginal Weck-Cel sampling will be used instead

Table 3a: Schedule of visits, immunisations and assessments for boosting [X means mandatory, (X) means if clinically indicated]

**Visit number**	**11***	**12**	**13**	**14**	**15**	**(16)[a]**
**Nature of visit**	**Dosing visit**	**Safety visit**	**Dosing visit**	**Safety visit**	**Follow-up visit**	**Follow-up visit**
**Week**	**20-32[b]**	**21-33**	**24-36 [b]**	**25-37**	**28-40**	**32-44**
**Visit window (days)**	**-3d+3d**	**-5d+3d**	**-3d+3d**	**-5d+3d**	**-7d+7d**	**-7d+7d**
Informed consent						
Medical history and demographics						
General examination and vital signs	(X)	X	(X)	X	(X)	(X)
Inspection of administration site[1]	(X)	X	(X)	X	(X)	(X)
Adverse event assessment	X	X	X	X	X	X
Urine pregnancy test	X		X		X	
Routine laboratory parameters[2]	(X)	X	(X)	X	X	(X)
Urinalysis		X		X	X	(X)
HIV test[3]	(X)		(X)		X	(X)
HBV, HCV and syphilis tests						
Screening for genital infections						
Vaginal secretions- Flora analysis						
Blood for serum – immunogenicity	X	X	X		X	X
Blood for PBMC – immunogenicity	X	X	X	X	X	X
Vaginal secretions – Instead cup[5]	X	X	X		X	X
Cervical & vaginal secretions –Weck-Cels						
**Immunisation[4]**	**X**		**X**			
Diary card						

*visit 10 and 11 can be combined as visit 10 falls 12 weeks post final vaccination in priming phase
[a] visit 16 will take place if time allows within the funding restraints
[b] vaccinations 4 and 5 (visit 11 & 13) should be 4 weeks apart

8 SAFETY REPORTING

UK clinical trials regulations require that both investigators and sponsors follow specific procedures when notifying and reporting adverse events/reactions in clinical trials. These procedures are described in this section of the protocol. Section 8.1 lists definitions, section 8.2 describes details of the responsibilities of the institutions/investigators and MRC CTU.

8.1 Definitions

Adverse event

Any untoward medical occurrence in a subject to whom a medicinal product has been administered, including occurrences which are not necessarily caused by or related to that product.

The investigator will use the following criteria when deciding whether to report a laboratory parameter that falls outside the normal range according to the local laboratory guidelines is an adverse event:

- The test result is associated with relevant accompanying symptoms
- Additional diagnostic tests or medication are indicated
- As a consequence of the test result, an immunisation is delayed or further immunisations are discontinued
- The investigator considers the result to constitute an adverse event for any other reason

Adverse reaction

Any untoward and unintended response in a subject to an investigational medicinal product which is related to any dose administered to that subject.

'Related' means possibly, probably or definitely as defined below.

Unexpected adverse reaction

An adverse reaction, the nature or severity of which is not consistent with the information about the medicinal product in question set out in the Investigator's Brochure.

Serious Adverse Events (SAEs), Serious Adverse Reaction (SARs), Unexpected Serious Adverse Reactions (SUSARs)

Any adverse event, adverse reaction, or unexpected adverse reaction is considered to be a "serious" according to the UK Clinical Trials Regulation (2004) if it:

- Results in death
- Is life-threatening
- requires hospitalisation or prolongation of existing hospitalisation
- results in persistent or significant disability or incapacity
- consists of a congenital anomaly or birth defect

Notes:

- '**A threat to life**' refers to an event or reaction in which the patient was at risk of death at the time of the event; it does not refer to an event or reaction which hypothetically might have caused death had it been more severe.

Any SAE that is considered possibly, probably or definitely related to study product according to the classification below meet the criteria for "unexpected".

Relationship to study product

This can be classified as:

**Unrelated**	adverse events that can be clearly explained by extraneous causes and for which there is no plausible association with study product, or adverse events for which there is no temporal relationship
**Unlikely to be**	adverse events that may be temporally linked, but which are much more likely to be due to other causes than study product and which do not get worse with continuing use of product
**Possibly**	adverse events that could equally well be explained by study product or other causes, which are usually temporally linked and may improve when not using study product but do not reappear when using study product
**Probably**	adverse events that are temporally linked and for which the study product is more likely to be the explanation than other causes, which may improve when not using study product
**Definitely**	adverse events that are temporally linked and for which the study product is the most likely explanation, which disappear or decrease when not using study product and reappear when using study product

8.2 Reporting adverse events

Adverse events should be recorded on the appropriate case report form (see section 7) and reported to the MRC CTU by faxing the CRF within two weeks of completion.

All **SAEs** should be reported to the MRC CTU within 1 working day of the Clinical Investigator becomes aware of the event fulfilling the criteria. This can be done by telephone, email or fax. The minimum criteria required in reporting a SAE are the participant identifiers (trial number/date of birth), reporting source (name of Investigator), why the adverse event is identifiable as serious.

Other important adverse events that should be reported to the MRC CTU within 1 working day of the Clinical Investigator becoming aware of the event, include

- allergic bronchospasm requiring intensive emergency treatment
- a seizure
- a cranial nerve palsy
- any adverse event that results in **discontinuation of the immunisation schedule**
- any adverse event that requires intervention to prevent a threat to life or death

SAE AND IMPORTANT AE NOTIFICATION

Within 1 working day of becoming aware of an SAE or Important AE, please fax a completed SAE form 7 to the MRC Clinical Trials Unit on:

Fax: 020 7670 4659

Or email: HIVvaccine@ctu.mrc.ac.uk

Or phone: 020 7670 4783 (Mon - Fri, 08:30 – 17:00)

They will be acknowledged by the MRC CTU immediately on receipt, and the Trial Management Group (section 16.1) notified by the MRC CTU the same day. If the Clinical Investigator does not receive an acknowledgement they will assume that MRC CTU is not aware of the event and use an alternative method of notification.

Staff at the MRC CTU will confirm that the event qualifies in seriousness and relationship as a **Suspected Unexpected Serious Adverse Drug Reaction (SUSAR)** and arrange for multi-disciplinary review of the case to take place within 3 working days of receiving the notification. Those involved in this review will include the site Clinical Investigator, the Medical Expert at MRC CTU, the Principal and Chief Investigators. If additional or independent expertise is required, individuals with experience interpreting preclinical and clinical data relevant to vaccines will be drawn from the UK HIV Vaccine Consortium Steering Committee.

The report will be prepared by the MRC CTU Medical Expert or their deputy and filed with the MHRA and main Research Ethics Committee within the timelines required by national legislation. MRC CTU will be responsible for circulating the report to the other clinical centre involved in the trial, and for coordination of the report review and filing with the Joint Research Office at SGUL. The site Principal Investigators are responsible for notifying their local R & D office.

The detailed process for the reporting and filing of SUSARs to the relevant authorities, as well as the writing of the annual safety report will be further defined in a trial specific SOP to be approved by the Sponsor.

8.3 Pregnancy

Pregnancy is not an adverse event. However, it is a reportable event in a Phase I trial, and should be reported to MRC CTU within 1 working day of the clinical investigator becoming aware of the pregnancy by email, fax or phone.

All pregnancies will be followed up to collect information about the outcome which will be recorded in the clinical study report.

Figure 2: Safety Reporting Flowchart

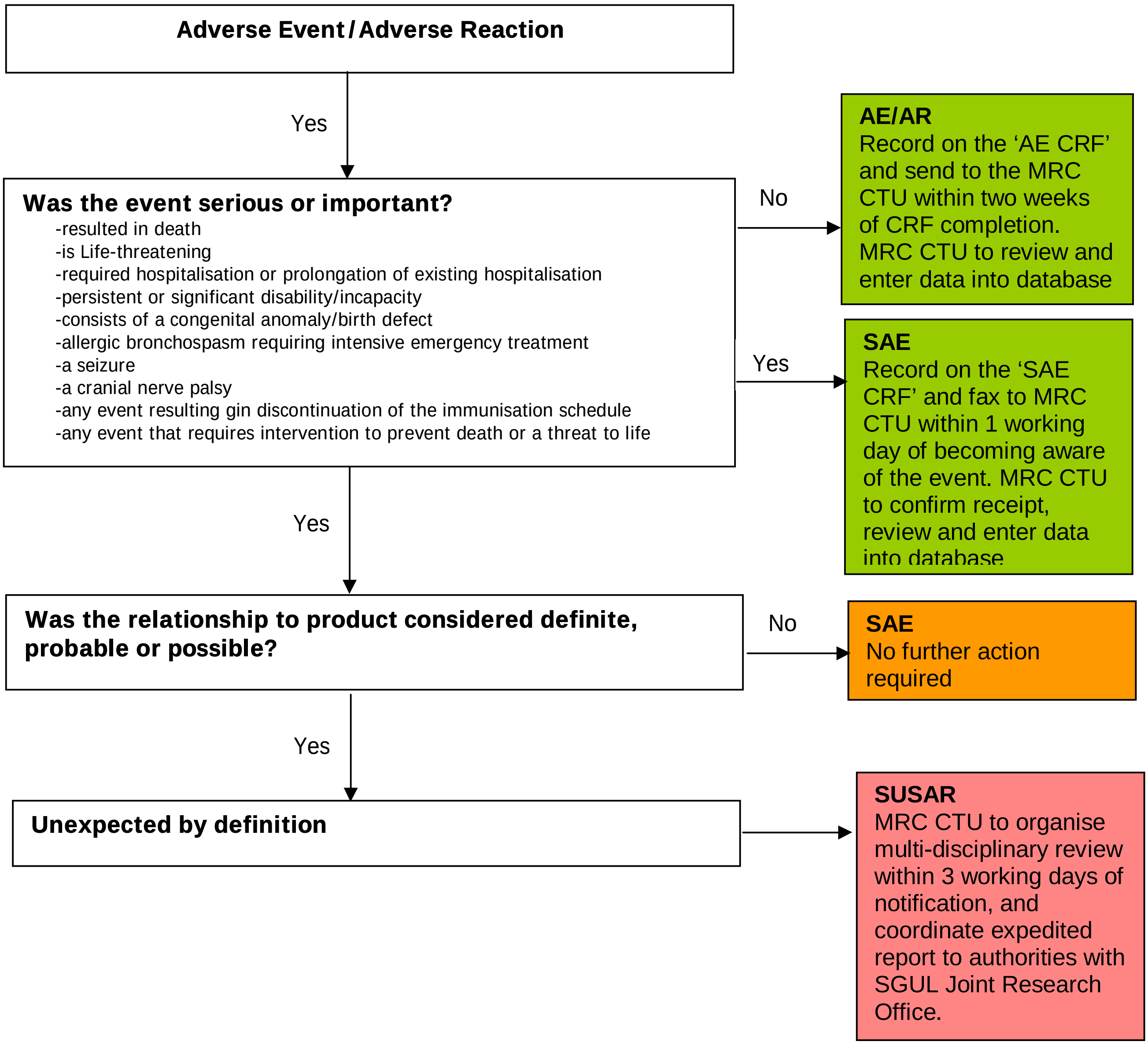

AE: Adverse Event
AR: Adverse Reaction
CRF: Case report form
SAE: Serious adverse event
SUSAR: Suspected unexpected serious adverse reaction

9 WITHDRAWAL OF PARTICIPANTS

Withdrawal means stopping all visits.

The reason(s) for withdrawal should be recorded in the CRF.

All participants are free to withdraw from the trial at any time, for any reason, without affecting their future medical care. An investigator may decide to withdraw a participant if the investigator deems that continuing might compromise participant wellbeing or interfere with the achievement of the trial's objectives.

Participants who are withdrawn due to an adverse event (AE) will be followed-up until the event has stabilised.

Withdrawn participants who have received any immunisations should be asked to undergo the procedures scheduled for the primary endpoint visit (Visit 8, week 12).

Policy for replacing withdrawals

There will be no replacement of participants withdrawn from the trial.

10 STATISTICAL CONSIDERATIONS

10.1 Method of Randomisation

Participants will be randomised centrally using a computer-generated algorithm based on random permuted blocks stratified over one factor, clinical centre. The algorithm will ensure that participants allocated to the IN group are enrolled at SGUL.

10.2 Outcome measures

Primary outcome

The primary immunogenicity outcome measures are the presence of an anti-CN54gp140 IgG antibody response and its strength (titre) in cervico-vaginal secretions at week 12. A sample will be classified as 'response detected' if the ELISA absorbance reading is greater than a cut-off which will be defined before the actual analysis; endpoint titration to quantify the strength of the response will be restricted to such samples.

Primary safety outcomes are grade 3 or above local or systemic (see section 7, Table 2) solicited adverse events and any adverse event that results in a clinical decision to discontinue further immunisations.

Secondary outcomes

Immunogenicity

- Presence and titre of anti-CN54gp140 IgG in cervico-vaginal secretions at other time-points
- Presence and titre of anti-CN54gp140 IgG in serum
- Presence and titre of anti-CN54gp140 IgA antibodies in cervico-vaginal secretions

Safety

- Any grade of adverse event that occurs in a participant that has received at least one immunisation

10.3 Sample size

It is not the remit of this Phase I trial to recruit a sufficient number of participants to be statistically confident about the result. However, by the end of this study 10 participants will have been exposed to each schedule in groups 1,2, and 4, and this provides confidence around the response/event proportions of 0, 1, 2 and 3 as follows:

Number of observed events	Proportion if n=10	95% confidence interval¶
0	0%	0 – 28%
1	10%	2 – 40%
2	20%	6 – 51%
3	30%	11 – 60%

¶ Wilson interval (suitable for small sample sizes)

It is difficult to give an estimate of the power of group comparisons using quantitative antibody titre outcomes at this stage as this is dependent on the number of responders.

10.4 Interim monitoring and analyses

Analyses will be performed at the MRC Clinical Trials Unit.

The accumulating safety and immunogenicity data will be reviewed once by an Independent Data Monitoring Committee (IDMC) (see also section 16.2) after half of the participants have completed the immunisation schedule. The IDMC will make a recommendation to the statistical team should any modifications to the analysis plan or statistical tables be required.

An unscheduled meeting of the IDMC may be required at the request of the Trial Management Group (see section 7.5), in which case the IDMC will make a recommendation about whether or not to continue further immunisations.

10.5 Data analyses and presentations

A full statistical analysis plan will be developed before the trial is analysed. It will be based on the following summary:

Participant populations

- Intention-to-treat (ITT) population: all participants randomised and given at least one immunisation in the trial.
- Per-protocol (PP) population: all participants randomised and immunised with all scheduled immunisations, and who complete the trial with no major protocol deviations.

Immunogenicity variables

ELISA absorbance readings for anti-CN54gp140 IgA and IgG antibodies in vaginal and cervical secretions, and in serum will be classified in 'response' or 'no response' using pre-defined cut-offs. Only samples with a detectable antibody response will be subjected to endpoint titration.

The number of 'responders' for each outcome will be presented by time-point and group with proportion and 95% confidence interval. An overall 'responder' is a participant in whom at least one post-treatment immunogenicity variable value was classified as 'response detected'. Comparison will be made using Fisher's exact test.

Titres of anti-CN54gp140 IgA and IgG antibodies will be described by time-point and group, and compared using rank tests.

Safety variables

The original verbatim terms used by the investigator to identify AEs in the CRFs will be coded using an appropriate medical coding scheme (MedDra v13). In all summaries, if a participant reports the same system organ class or preferred term more than once then the worst severity and worst relationship to trial vaccine will be taken. Discrepancies between diary card and CRF reports will be queried by MRC CTU. It is assumed that the grade assigned by the clinician is more accurate, and this will be the grade reported in the tables. If the diary card grade is worse, this will be foot noted.

All safety end-points will be graded by the Clinical Investigators and reviewed by the Trial Management Group. In the unlikely event that the TMG cannot reach consensus about grade and relationship to study product, an expert Endpoint Panel will be assembled. Members of the UK HIV Vaccine Consortium Steering Committee with vaccine trial experience and who are independent of the MUCOVAC2 trial will be asked to join this panel and make a final decision about the grade and relationship (see section 16; figure 2).

Safety outcomes will be reported overall with proportion and 95% confidence interval, and by group and time-point, and by relationship to study product.

For the primary analysis of safety endpoints (as defined in section 10.2), results will be expressed as a proportion with confidence interval, and groups compared using Fisher's exact test.

11 DATA MANAGEMENT

Data management, analysis and reporting of all trial data will be prepared by MRC CTU, according to the detailed Data Management and Statistical Analysis Plans.

11.1 Data management at the Clinical Centre

Staff at the clinical centres will be responsible for:

- Entering relevant information in the clinical notes and holding a record for each participant which includes the CRFs with any changes made signed and dated
- The accurate completion of the CRFs
- Collection of the diary cards from participants
- The prompt return of the completed CRFs and diary cards to the MRC CTU
- Notification of SAEs within 1 working day of becoming aware of the event to the MRC CTU
- Notification of pregnancy within 1 working day of becoming aware of the pregnancy to the MRC CTU

Data will be recorded directly onto the clinical notes and the CRFs in the most logical order, which may not necessarily always be the clinical notes followed by the CRF. For example the recording of the solicited adverse events will be done directly onto Form 4 with additional details provided in the clinical notes if required. Similarly if the screening CRF does not reveal any medical history of relevance then this negative finding will not be duplicated in the clinical notes. Additional detail will be expected should there be a clinical abnormality relevant to eligibility. The dates of visits including immunisation, dates and results of pregnancy tests, and details of clinical management (description of adverse events and concomitant medication) will be detailed in the clinical notes.

Other source data are described in table 4.

Table 4: The following table identifies the source documents:

Type of data	Source document
Informed consent	Signed informed consent form
Medical history	Clinical notes and screening CRF Form 0
Physical examination, including height and weight	Clinical notes and screening CRF Form 1a
Demographics	Registration sheet and Screening CRF Form 0
Laboratory data (except for urinalysis dipstick and urine pregnancy test results)	Laboratory reports
Date/time of routine bloods; urinalysis dipstick and urine pregnancy test sampling and results	Clinical notes and CRF Form 5
Date/time/result of solicited AEs including vital signs measurements post immunisation	CRF Form 4
Date/time of immunisations	Clinical notes and CRF Form 4
Date/time of immunogenicity sampling	Clinical notes and CRF Form 11
Immunogenicity results	Core Immunogenicity Laboratory File

IMP accountability	Pharmacy File
Adverse events	Clinical notes and CRF Form 6 or 7
Concomitant medication	Clinical notes and CRF Form 8
Protocol deviations	Clinical notes and CRF Form 9
Participant reported solicited AEs and concomitant medication	Diary cards

CRFs will be designed and provided to the sites by MRC CTU.

All data will be entered legibly in black ink with a ball-point pen. If an error is made, the error will be crossed through with a single line in such a way that the original entry can still be read. The correct entry will then be clearly inserted and the alterations will be initialed and dated by the person making the alteration. Overwriting or use of correction fluid will not be permitted.

To preserve confidentiality, the CRFs will not bear the participant's name. The participant's date of birth and trial number will be used for identification.

Copies of laboratory reports containing the results of routine haematology, chemical pathology and immunology may be sent instead of completing these sections of the CRF, provided they are clearly labelled with the trial number and the date of collection. A member of the clinical trial team must sign the laboratory report. In the event of an abnormality, an indication should be given whether or not action was taken, the date of review and the signature of the clinician reviewing the result.

CRFs and clinical notes should be kept in a secure location for 2 years after the last approval of a marketing application or until 2 years have elapsed since formal discontinuation of product development, and at least 15 years after the clinical trial has ended.

11.2 Data management in the immunology laboratories

Standard operating procedures will be followed in all laboratories to ensure the quality of the data. Data will be stored electronically in an agreed format and datafiles transferred to the data management centre for the main analysis.

11.3 Data management at the MRC CTU

MRC CTU will be responsible for:

- Design of the CRFs in collaboration with the Investigators
- The database applications that will contain the computerised trial data
- Data entry for the case report forms and diary cards
- Importing the immunological data from the core laboratories
- Monitoring the trial according to principles of GCP including monitoring vaccine accountability, and dispatch and arrival of immunological specimens
- Preparation of reports to assist the monitoring
- Holding a record for each participant which contains the copy of the CRF and documentation detailing all the changes made subsequent to monitoring visits, queries raised and how they were addressed
- Coordination of the committee and group meetings (section 16) in collaboration with the Investigators
- Development of the analysis plan and conducting the analyses
- Preparation of the analysis files from the database prior to analyses
- Coordination of the final report

12 TRIAL MONITORING

12.1 Risk assessment

The MRC CTU has performed a risk assessment to assess the risks and benefits of trial participation to the individual participant safety, as well as the risks that underlie the validity of the trial results with respect to safety and immunogenicity outcome measurements.

Particular consideration was given to the procedures for reconstituting the CN54gp140 in three different doses ranging from 20-500µg in four different formulations, and this informed the decision to make the trial open.

The risk assessment has been discussed and approved by the MRC CTU Quality Management Committee. The risk assessment is stored independently of this document.

The outcome of this assessment has been used to guide the development of procedures with respect to informed consent, confidentiality, trial monitoring and audit.

SGUL has also performed a risk assessment, stored independently of this document and this has been used to guide the development of the safety reporting and pharmacy procedures.

12.2 Monitoring at MRC CTU

All CRFs and laboratory reports returned to the MRC CTU will be reviewed by the Data Management Team, according to standard operating procedures. CRFs will be checked for completeness and passed for review by the Medical Expert if required. Data will be entered into a computerised database. Consistency checks and range checks will be embedded in the database.

The accuracy of data entry will be checked according to standard operating procedures at the data management centre.

The trial manager or their deputy will review adverse events, as they arise. Queries raised will be directed to the investigators at the relevant clinical centre by letter, fax, email or at a monitoring visit.

Prior to any analysis, the safety data will be checked, adverse events validated and data extracted in order for the trial statistician to run the analysis and prepare the tables.

12.3 Clinical site monitoring

The MRC CTU will produce a detailed Monitoring Plan. Trial sites will be monitored to ensure:

- the completeness and accuracy of the data entered on the CRFs
- compliance with the protocol and principles of GCP
- proper maintenance of all trial documentation
- complete IMP accountability
- smooth day-to-day running of the trial

All documents generated by the trial sites which form part of this trial, and the ensuing data, must be made directly available in order that the monitor can verify, using source documents, the data in the CRFs. This procedure is termed Source Document Verification (SDV).

The PIs agree to allow GCP audits of the trial sites and all trial documentation by the Sponsor or its representatives.

Direct Access to Data

Participating investigators should agree to allow trial-related monitoring, including audits, ethics committee review and regulatory inspections by providing direct access to source data/documents as required. Participants' consent for this is obtained as part of the consent process.

12.4 Monitoring by the Trial Management Group

The Trial Management Group (described in section 16.1) will monitor the following using a reporting template:

- Screening, enrolment and screen:enrolment ratio for each clinical centre
- Immunisations completed (first, second third) and any missed or outside the window
- Adverse events of note
- Missed visits and loss to follow-up
- Logistical difficulties at the clinical centres
- Data management issues (timeliness of CRFs, completeness)
- Immunology core lab issues (completeness of specimens, next batch transfer or analysis)
- GCP issues (minor or other breaches)

12.5 Confidentiality

All personal details of the participants and the results of the trial will be kept strictly confidential. The Sponsor, as represented by the SGUL JRO, will not keep any material on file containing the volunteers' full names; this information will be kept by PIs in the clinical trial facilities in a secure location. The confidentiality of volunteers will be respected and maintained at all times.

Each participant's GP (or equivalent physician) will be informed of the nature and timing of the trial and asked to complete a brief questionnaire.

12.6 Quality Assurance and Quality Control of Data

Sites must not screen any volunteers until the Sponsor and MRC CTU approvals are in place, and MRC CTU has conducted a site initiation visit. The Sponsor approval is dependent on receipt of a Clinical Trial Authorisation (CTA) from the Medicines and Healthcare Regulatory Authority (MHRA), approval from the main Research Ethics Committee (MREC), and site specific approval from the relevant NHS R&D offices (see section 13.1).

It will be the responsibility of the PIs to ensure the accuracy of all data entered in the CRFs at their respective sites. They must conduct the trial personally, or delegate to members of their research team specific tasks using a delegation log. They must ensure that each member of their research team is suitably qualified to perform delegated tasks by education, training and experience, and must ensure that written procedures are followed to enable collection high quality data.

13 ETHICAL CONSIDERATIONS AND APPROVAL

13.1 Ethical issues

There are three aspects of this trial that raise ethical issues

Firstly, because of the **limited human safety data on the investigational product**. Recombinant protein vaccines are in widespread use as licensed vaccines, and there have been 3 large Phases III trials of similar envelope proteins that did not raise any safety concerns. However, these were given by the IM route, and much less is known about the IN and IVAG routes. To mitigate against this concern the visit schedule is intense, and the enrolment will be timed to permit one individual to process through the first safety visit following IN or IM administration before any others are enrolled.

Secondly, because the product under investigation is a **candidate HIV vaccine**, and HIV is transmitted sexually, it is necessary to collect sensitive personal information and the volunteers will need to undergo a genital examination. The nature of the product may lead volunteers to erroneously conclude they are protected against HIV and to engage in riskier behaviour as a consequence. It is possible that following immunisations, participants may have equivocal results in the standard laboratory tests for HIV. However, it will be possible for any accredited laboratory to distinguish between a post-vaccination response and natural infection using routine assays.

Thirdly **the reimbursement** to compensate for the intense follow-up schedule, which is a feature of healthy volunteer trials, could be sufficient incentive for individuals to take part against their better judgement.

13.2 Ethical considerations

The trial will be conducted in compliance with UK Clinical Trial Regulations and any amendments, which include compliance with the principles of Good Clinical Practice (GCP) and will abide by the principles of the Declaration of Helsinki

The trial proposal will be reviewed by a recognised REC, and by the MHRA. These reviews will include a critique of the participant information sheet, which forms the basis of the information provided to volunteers before they are asked if they agree to be screened. The trial will not proceed unless the sponsor obtains a clinical trial authorisation (CTA) from the MHRA, and approval from the main REC. The trial will not proceed at site until NHS R&D office approval for the site has been given.

All volunteers must give written consent to participate in this trial. The trial-specific consent form will be signed by the volunteer before any screening evaluation. Before giving consent, volunteers will be asked to read the information sheet about the trial and raise questions. They must also read the consent form. They will have the opportunity to discuss the trial with a PI or their deputy and be asked to explain what the trial involves and describe the risks and benefits in their own words, so that the PI or their deputy can ensure that the participant understands. The trial-specific information sheet and the consent form must be approved by the main REC.

The safety assessments are intense. Participants will be asked to remain in clinic for 1-2 hours following each immunisation, to complete a diary card for at least 7 days thereafter, and return for a safety visit a week after each immunisation. They will be advised to call the clinic staff if they are concerned, and 24 hour cover will be available. The sponsor will ensure that procedures are in place to inform the MHRA and the main REC promptly of SUSARs arising in this trial, or of new SUSARs arising in other ongoing trials of the IMPs under investigation that come to the attention of the sponsor through the literature.

MRC CTU will promptly inform the Chief Investigator, and the Sponsor, of any SAE that occurs during this trial, regardless of relationship to the investigational product.

The Sponsor will ensure, by delegating this responsibility to MRC CTU that an annual safety report is provided to the Principal Investigators at each clinical centre, the main REC and the MHRA, and that this includes a description of all suspected unexpected serious adverse reaction (SUSAR) reports.

MRC CTU staff or the PIs will promptly inform the Sponsor if they suspect a serious breach of GCP or the trial protocol has occurred according to the criteria stated in the MHRA's guidance. The Sponsor, using the same criteria, will make the decision and notify the MHRA and other bodies such as main REC and NHS R&D office, within seven days of becoming aware of a serious breach.

MRC CTU staff will regularly inform the Sponsor of all breaches of GCP and deviations that impact on safety or validity by circulating the notes of the Trial Management Group calls to the CI. The CI will report to the main REC and to NHS R&D office any breaches or deviations that are, in his opinion, of major significance. Minor breaches and deviations will be summarised in the annual reports prepared by MRC CTU and circulated to the MHRA, the main REC and the local NHS R&D.

Within 90 days after the end of the trial, defined as the final visit of the last participant, the Sponsor will ensure that the main REC and the MHRA are notified that the trial has finished. If the trial is terminated prematurely, those reports will be issued within 15 days after the termination date which is defined as the final participant visit.

The Sponsor will supply a summary report of the clinical trial to the MHRA and main REC within one year after the end of the trial.

14 INDEMNITY

The Sponsor for the trial is St George's University of London (SGUL).

The Sponsor undertakes to compensate any volunteers for injuries which are considered, on the balance of probabilities to have arisen as a result of their participation in the trial regardless of whether the injuries were caused by negligence or not.

SGUL holds insurance to cover participants for injury caused by their participation in the clinical trial. Participants may be able to claim compensation if they can prove that SGUL has been negligent. However, as this clinical trial is being carried out in a hospital, the hospital continues to have a duty of care to the participant in the clinical trial. SGUL does not accept liability for any breach in the hospital's duty of care, or any negligence on the part of the hospital employees. This applies whether the hospital is an NHS Trust or not. This does not affect the participant's right to seek compensation via the non-negligence route.

Participants may also be able to claim compensation for injury caused by participation in this clinical trial without the need to prove negligence on the part of SGUL or another party. Participants who sustain injury and wish to make a claim for compensation should do so in writing in the first instance to the Chief Investigator, who will pass the claim to the Sponsor's Insurers, via the Joint Research Office.

The University of York shall provide clinical negligence insurance cover for harm caused by their employees and a copy of the relevant insurance policy or summary shall be provided to SGUL upon request.

15 FINANCE

The clinical trial activities and acquisition of product are funded by the Wellcome Trust under the Grand Challenges in Global Health Initiative.

The data management, monitoring and analysis activities are funded by the Wellcome Trust under the UK HIV Vaccine Consortium.

Participants will receive recompense for their time and travel, and the amount will be reviewed by the main REC.

There are no bonuses or per participant incentives paid to staff.

16 TRIAL COMMITTEES

16.1 Trial Management Group (TMG)

The Trial Management Group (TMG) will be formed of the Chief and Principal Investigators and other lead Investigators (clinical and non-clinical) from the two centres and core immunology laboratories as well as members of the MRC Clinical Trials Unit.

The TMG will be responsible for the day-to-day running and management of the trial and will be accountable to the Sponsor.

The TMG will also be responsible for the composition of the expert panel to review any emergent SUSARs and for requesting an unscheduled IDMC.

16.2 UK HIV Vaccine Consortium Steering Committee (TSC)

This committee has reviewed the design of the trial as part of the approval of release of funding for the data management and coordination provided by MRC CTU.

The UK HVC Steering Committee's purpose and membership is described on the UK HVC website (http://www.ukhvc.org/the-uk-hvc/management/steering-committee/)

If additional or independent expertise if required to review a SUSAR, then individuals will be drawn from the list of independent members of the UK HVC Steering committee and asked to join the expert panel review organised by MRC CTU (section 8.2).

16.3 Independent Data Monitoring Committee (IDMC)

An Independent Data Monitoring Committee (IDMC) will be invited to oversee this trial, and none of its members have direct involvement with the trial. The IDMC will report to the TMG via MRC CTU for scheduled meetings, and directly to the Sponsor should an unscheduled meeting be required to review a SUSAR or other significant adverse event. A charter will be developed to describe the functioning of the IDMC. Details of the interim analysis and monitoring are provided in section 10.4 and will be summarised in an IDMC charter.

Figure 3: Diagram of relationships between trial groups and committees

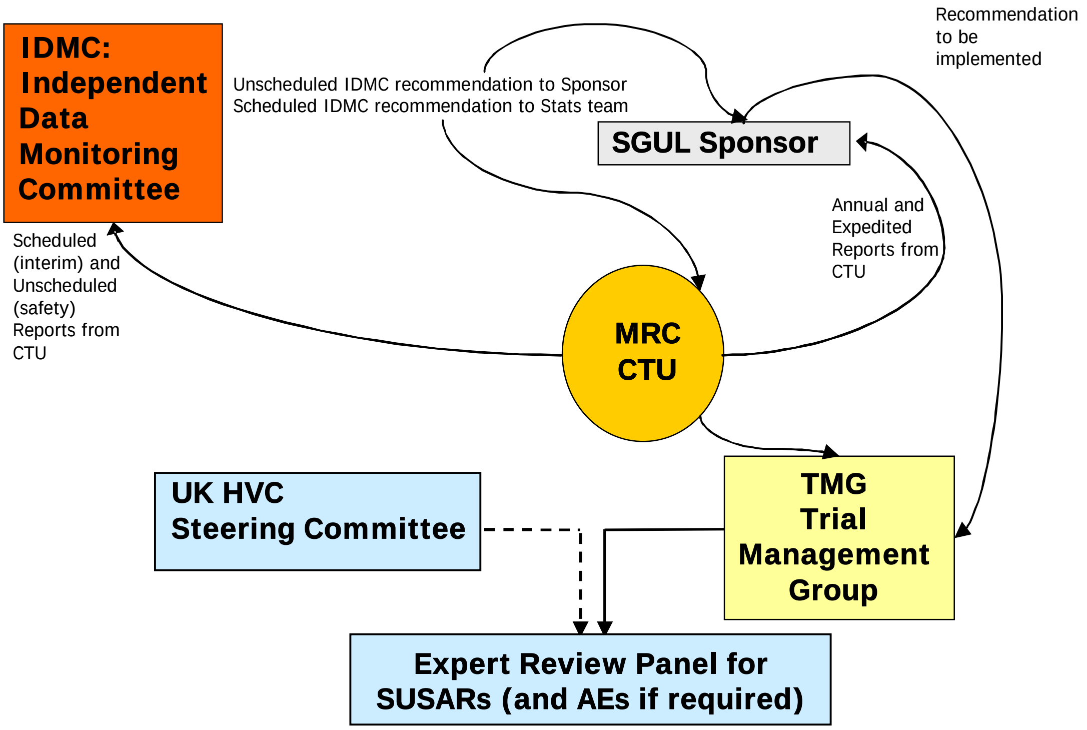

17 PUBLICATION

The preparation of a manuscript for publication in a peer-reviewed professional journal or an abstract for presentation, oral or written, to a learned society or symposium will be discussed on the Trial Management Group calls. The Sponsor will be notified of this intention through the Chief Investigator and the TMG notes. Every effort will be made to allow the Sponsor and other relevant parties involved in the clinical trial and named in the clinical trial agreement prepared by the Sponsor, 30 days to comment before any results are submitted. This timeline will be strictly observed for peer-review journals, but may be more difficult to adhere to for conference presentations. Approval from the Chief Investigator, the clinical centre Principal Investigators and at least one member of the MRC CTU trial team must be obtained as a minimum before submission to a conference.

Authorship should reflect work done by the investigators and personnel of the sponsor, in accordance with generally recognised principles of scientific collaboration.

18 PROTOCOL AMENDMENTS

After the protocol has been approved by the main REC and the MHRA, no changes may be made without the written agreement of both the investigators and the sponsor.

The MHRA and main REC do not need to approve any substantial change to the protocol that needs to be implemented urgently to avoid an immediate hazard to trial participants. The sponsor will ensure that the MHRA and main REC are informed of urgent amendments in accordance with UK clinical trials regulatory guidance.

The REC and/or MHRA must approve substantial amendments before they are implemented.

<u>Version 1.1 31/03/2011</u>

<u>At request of MHRA</u>

To ensure safety as “administered systemically for the first time in humans” –

Volunteers in UK received up to 4 immunisations of DNA or NYVAC carrying the CN54 derived genes in three preceding vaccine trials without concern (EuroVacc 01, 02 and 03) - added to section 2.2.2.

and in section 5.1:
“The rate of enrolment will be controlled centrally.

- A second participant will not be randomised to receive IM or IN immunisation until the first participant who has received an immunisation by that route has reached her first follow up safety visit (~7days post immunisation).
- There will be a 7 day pause in enrolments after the third individual has been allocated to IM 100μg rgp140 + GLA to ensure that there is a similar gap in the safety visits following the second immunisation.“

replaced with

The rate of enrolment will be controlled centrally.

- The first two participants enrolled will complete the safety visit following the first IM rgp140 100μcg (York) and the first IN rgp140 100μcg (St George’s) before any other individuals are randomised
- This gap between the first two individuals enrolled and the subsequent cohort will remain in the trial schedule for the second and third immunisations, providing sufficient time to interrupt further immunisations in the unlikely event that this is necessary

Addition to inclusion criteria (section 4.1, also added to information sheet and GP letter)
7. Normal cervical smear within 12 months of screening

<u>At request of South East London REC1</u>

Information sheet – Paragraphs added to clarify purpose of the study and to make it clear that participants will not be immunised against HIV
What are the possible disadvantages and risks of taking part? Paragraph 4 expanded to clarify effect of study

GP letter – “as far as you are aware” added to paragraph 3

<u>Version 1.2 10/05/2011</u>
Page 2 – St George’s NHS Healthcare Trust added as the new site and Dr Catherine Cosgrove added as local PI

Page 3 – New MRC CTU address added

Page 20 section 2.2.2 final paragraph updated to reflect the move of the immunology core laboratory from SGUL to Imperial College

Section 7.3 (page 34) and Patient information sheet section 7 - INSTEAD Softcup timing changed from 5-10 minutes to at least 1 hour as it was felt 5-10 minutes was not sufficient to collect sample

Appendix 3a GP questionnaire – question added for date and result of recent Cervical Cytology Smear test

Version 1.3 22/07/2011

References to Bill and Melinda Gates Foundation removed to reflect their withdrawal of funding. (pages 2, 9, 53 and 68)

Additional core immunology laboratory added and text amended to reflect 2 core laboratories (pages 3, 20 and 47)

Version 1.4 27/09/2011

CI at St Georges changed from Professor David Lewis to Dr Catherine Cosgrove (page 2 & appendix 3a page 70)
Clarification that the first participant in each centre will complete the safety visit following their first immunisation before other participants are enrolled at that centre (page 19 section 2.2.2. and page 25 section 5.1).
Destination of samples clarified on the participant information sheet section 7
On patient information sheet section 14 "anonymised" replaced by "your name and address will be removed from the data"

01/11/2011

At the request of the REC the following have been added:
To the Patient information sheet (Appendix 1):
If once the samples have been analysed there is any remaining they will be destroyed.
To the consent form (Appendix 2):
6. I give permission for my stored samples to be made available for research where the samples would be stored appropriately and some may be sent outside the UK
These have both been redated but remain part of this protocol

Version 1.5 16/01/2012

Cover – signatory for the Sponsor SGUL changed due to the departure of Dr Paul Craven

Page 2 - Dr Georgina Morris has been added as temporary PI at York as Professor Lacey will be out of contract for 1 month from 1st to 29th February 2012 (GP letter updated accordingly)

Added to Section 7.3 - a serum separator tube (SST, gold top) to allow choice as clot appears when plain tubes are used for some participants.

Also added to Section 7.3 and referenced in Table 3 - For those participants with an IUCD in situ 2 Weck-Cel sponges will be inserted into the vagina using sponge forceps without the need for a speculum examination and held there for one minute by a health care professional.

Added to participant information sheet (appendix 1) section 7 - in which case vaginal Weck-Cel sampling will be used. Date & version on this and consent form (appendix 2) updated accordingly.

Version 2.0 11/06/2012

This is a sub-study within MUCOVAC2 is to assess the impact of intramuscular boosting following intranasal priming on systemic and mucosal responses and to compare responses to intramuscular boosting following intramuscular priming.

Cover – signatory for the sponsor SGUL changed due to the departure of Ira Jakupovic

Dr Catherine Cosgrove's title updated to Honorary Consultant in Infectious Diseases and General Medicine

Section 1 updated with additional boost schedule & details, table 1a added

Section 1.1.1 design updated

Section 1.1.5 duration updated

Section 1.2 Flow diagram, updated to reflect boost schedule

Section 2.3 added to give background and rationale for additional boosting

Section 4.1 inclusion criteria updated

For participants proceeding to boost phase:

14. satisfactory completion of the priming vaccinations and followup in the main study
15. willing to continue in the study for at least a further 12 weeks with 5-6 extra visits

Section 4.3 numbers updated
Up to 10 participants (5 from IN group and 5 from IM 100µg group) approaching or having reached visit 10 will be invited to be boosted with 2 further IM 100µg vaccinations 4 weeks apart with the first 12-24 weeks after last priming vaccination. They will be provided with an information sheet and asked to sign a consent form.

Section 4.5 added to detail rescreening procedures

Section 6.1.1 updated with boost IM details

Section 7.1 duration of follow-up and schedule updated

Section 7.6 end of trial updated to visit 15 or 16

Table 3a added as schedule for the boosting phase

Section 19 references – 49 added as justification for boost

Appendix 1a (information sheet for boost) and 2a (Consent for Boost) added (date and version on originals remains unchanged)

Appendix 3c (GP letter boosting phase) added

Appendix 5 amended to add 2 extra vaccinations

19 REFERENCES

1. UNAIDS report 2009. http://www.unaids.org/en/default.asp (accessed on March 25th 2010

2. IAVI. Estimating the global impact of an AIDS vaccine. 2006. IAVI Policy brief

3. Rerks-Ngarm S et al., Vaccination with ALVAC and AIDSVAX to prevent HIV-1 infection in Thailand. 2009. N Eng J Med. 361:2209-20

4. Flynn NM et al., Placebo-controlled phase 3 trial of a recombinant glycoprotein 120 vaccine to prevent HIV-1 infection. J Infect Dis 2005; 191:654–665.

5. Pitisuttithum P et al., Randomized, double-blind, placebo-controlled efficacy trial of a bivalent recombinant glycoprotein 120 HIV-1 vaccine among injection drug users in Bangkok, Thailand. J Infect Dis 2006; 194:1661–1671.

6. Buchbinder SP t al.,Efficacy assessment of a cell-mediated immunity HIV-1 vaccine (the Step Study): a doubleblind, randomised, placebo-controlled, test-of-concept trial. Lancet. 2008; 372:1881–1893.

7. D'Souza MP and Frahm N. Adenovirus 5 seroptype vector-specific immunity and HIV-1 infection; a tale of T cells and antibodies. AIDS. 2010; 24:803-809

8. Rowland-Jones S et al., HIV-specific cytotoxic T-cells in HIV-1 exposed but uninfected Gambian women. Nature Med 1997;1:59-64

9. Mazzoli et al., HIV-1 specific mucosal and cellular immunity in HIV-seronegative partners of HIV-1 seropositive individuals. Nature Med 1997;3:1250-57

10. Wilson NA et al., Vaccine-induced cellular responses control simian immunodeficiency virus replication after heterologous challenge. J Virol 2009;83:6508-6521

11 Liu J et al., Immune control of an SIV challenge by a T-cell based vaccine in rhesus monkeys. 2009. Nature 457:87-91

12. Hansen SG et al., Effector memory T cell responses are associated with protection of rhesus monkeys from mucosal simian immunodeficiency virus challenge. Nature Med 2009. 15:293-299

13 Chung C et al., Not all cytokine producing CD8+ T cells suppress simian immunodeficiency virus replication. J Virol 2007;81:1517-23

14. Spentzou A et al., Viral inhibition assay: A CD8 T cell neutralization assay for use in clinical trials of HIV-1 vaccine candidates. J Inf Dis 2010; 201:720-29

15. Scheid JF et al., Broad diversity of neutralizing antibodies isolated from memory B cells in HIV-infected individuals. Nature 2009:458;6636-40

16. Pancera M et al., Structure of HIV-1 gp120 with gp41-interactive region reveals layered envelope architecture and basis of conformational mobility.PNAS 2010; 107:1166-71

17. Chen L et al., Structural basis of immune evasion at the site of CD4 attachment on HIV-1 gp120. Science 2009;326:1123-27

18. Hubner et al.,quantitative 3D video microscopy of HIV transfer across T- cell virological synapses. Science 2009;323:1743-47

19. Walker LM et al., Broad and potent neutralizing antibodies from an African donor reveal a new HIV-1 vaccine target. Science . 2009;326:285-9

20. Hessell AJ et al., Effective, low-titer antibody production against low-dose repeated mucosal SHIV challenge in macaques. Nature Med 2009;15:951-54

21. Hessel AJ et al., Broadly neutralizing human anti-HIV antibody 2G12 is effective in protection against mucosal SHICV challenge even at low serum neutralizing titres. PLoS Pathog 2009;5 e1000433

22. Johnston MI and Fauci AS. An HIV vaccine—challenges and prospects. N Engl J Med 2008; 359:888–890.)

23. McCormack S et al., EV02 a phase I trial to compare the safety and immunogenicity of HIV DNA-C prime-NYVAC-C boost to NYVAC-C alone. Vaccine 2008;26:3162-74

24. Sandstrom E et al. Broad immunogenicity of a multigene, multiclade HIV-1 DNA vaccine boosted with heterologous HIV-1 recombinant modified vaccinia virus Ankara. J Infect Dis 2008;198(10):1482–90.

25. Excler JL et al., A strategy for accelerating the development of preventative AIDS vaccines. AIDS 2007;21:2259-63

26. Hu SL et al., Protection of macaques against SIV infection by subunit vaccines of SIV envelope glycoprotein gp160. Science 1992;255:456-59

27. Lu S. Heterologous prime boost regimens. Curr Opin in Immunology. 2009: 21:346-51

28. McMichael AJ et al., The immune response during acute HIV-1 infection; clues for vaccine development. Nat Rev Imm 2010;10:11-23

29. Virgin HW and Walker BD. Immunology and the elusive AIDS vaccine. 2010. Nature 464:224-31

30. Wang SW et al., An SHIV DNA/MVA rectal vaccination in macaques provides systemic and mucosal virus-specific responses and protection against AIDS. AIDS Res Hum Retro-viruses 2004;20:846-59

31. Vajdy M et al., Mucosal and systemic anti-HIV responses in rhesus macaques following combinations of intranasal and parenteral immunizations. AIDS Res Hum Retro-viruses 2004;20: 1269-81

32. Gomez-Roman et al., Vaccine-elicted antibodies mediate antibody-dependent cellular cytotoxicity correlated with significantly reduced acute viraemia in rhesus macaques challenged with SIVmac251. J Immunol 2005; 174:2185-9

33. Belec L et al., Cervicovaginal Secretory Antibodies to Human Immunodeficiency Virus Type 1 (HIV-1) that Block Viral Transcytosis through Tight Epithelial Barriers in Highly Exposed HIV-1–Seronegative African Women. J Inf Dis 2001;184:1412-22

34. Kaul R et al., HIV-1 specific mucosal IgA in a cohort of HIV-1 resistant Kenyan sex workers. AIDS 1999; 13:23-9

35. Veazey RS et al., Prevention of virus transmission to macaque monkeys by a vaginally applied monoclonal antibody to HIV-1 gp120. Nat Med 2003; 9:343-6

36. Haynes BF and Shattock RJ. Critical issues in mucosal immunity for HIV-1 vaccine development. J Allergy Clin Immunol 2008;122:3-9

37. Test facility study number 516596;report number 30962: 8 week toxicity and tolerance study of CN54gp140 in rabbits by intramuscular, intranasal or intravaginal administration.

38. Lewis D et al., Phase 1 safety and immunogenicity randomised controlled trial of a vaginal gp140 vaccine. (Abstract: P11-06). AIDS Vaccine 2009.

39. Human GLA – Muvovac2 Investigators Brochure

40. Test Facility Study No. 516926, Report No. 31061: 4 Week Intramuscular Toxicity and Tolerance Study of Glucopyranosyl Lipid Adjuvant (GLA) in Rats.

41. Cranage M et al., Intravaginal administration of HIV-1ZM96 gp140 augments systemic and mucosal antibody responses following systemic priming with adjuvanted protein. (Abstract: P14-06). AIDS Vaccine 2009.

42 Hurwitz JL et al., First Phase clinical trial of an HIV-1 subtype D gp140 envelope protein vaccine: immune activity induced in all study participants. AIDS 2008; 22;149-58).

43 Pialoux G et al., Phase I study of a candidate vaccine based on recombinant HIV-1 gp160 (MN/LAI) administered by the mucosal route to seronegative volunteers; the ANRS VAC14 study. Vaccine 2007;26:2657-666.

44. Travers SA et al., Timing and reconstruction of the most recent common ancestor of the subtype C clade of human immunodeficiency virus type 1. J Virol 2004;78: 10501-06.

45. Mills KHG et al.,. Protective levels of diphtheria-neutralizing antibody induced in healthy volunteers by unilateral priming-boosting intranasal immunization associated with restricted ipsilateral mucosal secretory immunoglobulin A. Infect Immun 2003;71: 726–732.

46 Huo Z, Induction of protective serum meningococcal bactericidal and diphtheria-neutralizing antibodies and mucosal immunoglobulin A in volunteers by nasal insufflations of the Neisseria meningitides serogroup C polysaccharide-CRM197 conjugate vaccine mixed with chitosan. Infect Immun 2005; 73: 8256–8265.

47. Wassen L, et al. Local intravaginal vaccination of the female genital tract. Scand J Immunol 1996 ;44: 408–414.

48. Goepfert, et al. Durable HIV-1 antibody and T-cell responses elicited by an adjuvanted multi-protein recombinant vaccine in human volunteers. 2007. Vaccine ;25: 510-18.

49. Barnett et al. Protection of macaques against vaginal SHIV challenge by systemic or mucosal and systemic vaccinations with HIV-envelope. AIDS 2008, 22; 339-348

APPENDICES

APPENDIX 1: PARTICIPANT INFORMATION SHEET..67
APPENDIX 1A: PARTICIPANT INFORMATION SHEET- BOOSTING SUB-STUDY 73
APPENDIX 2: CONSENT FORM..79
APPENDIX 2A: CONSENT FORM FOR BOOSTING SUBSTUDY 80
APPENDIX 3a: GP LETTER (pre-trial) 81
APPENDIX 3b: GP LETTER...86
APPENDIX 3c: GP LETTER BOOSTING PHASE...87
APPENDIX 4: TOXICITY TABLE 88
APPENDIX 5: DIARY CARD 91

APPENDIX 1: PARTICIPANT INFORMATION SHEET

(To be presented on local headed paper)

PARTICIPANT INFORMATION SHEET

Date and version: 16/01/2012 v1.5

Acronym of study: MUCOVAC2

1. Study title

A Phase I clinical trial to assess the safety and immunogenicity of three HIV CN54gp140 immunisations administered through the intramuscular, intranasal and intravaginal routes in healthy female volunteers

2. Invitation paragraph

You are being invited to take part in a research study. Before you decide it is important for you to understand why the research is being done and what it will involve. Please take time to read the following information carefully and discuss it with others if you wish. Ask us if there is anything that is not clear or if you would like more information. Take time to decide whether or not you wish to take part. Thank you for reading this.

3. What is the purpose of the study?

There is an urgent need to develop methods to prevent HIV infection, given the continuing world-wide epidemic and an estimated 14,000 new infections a day.
Vaccines are products that are given to people in advance to help protect them from developing illnesses caused by particular infections. If successful, they work by stimulating and preparing the body's immune system (the cells and tissues that fight infection) so that if a person is exposed to a specific infection in the future, it can be recognised and cleared before the person becomes unwell.
To date, several potential HIV vaccines have been developed, and three have been assessed in large populations at risk of HIV infection to see if they are able to prevent any infections. Results with the first two were disappointing but the third showed a small benefit. Although the benefit was too small to lead to a successful vaccine, the positive result has given hope, and led to renewed efforts to strengthen potential HIV vaccines.

The aim of this particular study is to assess the safety of a new potential HIV vaccine. We'll also assess how well the vaccine stimulates the body's immune system by testing your blood in the laboratory. However, this study is **not** designed to test whether the vaccine actually provides real life protection against HIV. Even if you participate in this study and your immune system is stimulated by the vaccine, this **does not** mean that you will have been protected against HIV and it will still be possible for you to acquire HIV in the future.

Unlike some other vaccines, the potential vaccine we are testing **does not** contain any active (live) or denatured (killed) whole HIV or any other vector (carrier) viruses. Instead, it consists of a protein (a part known as gp140) that is found on the surface of HIV. Our potential vaccine is similar to some HIV vaccines that have been tested previously, including one used in the trial that showed a small benefit, but the way it will be given is different.
At present, it isn't clear which route of giving an HIV vaccine is best at being able to stimulate the immune system and prevent the virus from being acquired through sex. The vaccine we are testing has previously been shown to be safe when given to a small number

of women intravaginally. Our study will look at giving the same potential HIV vaccine in 4 different combinations using three different routes (into the muscle, up the nose or in the vagina) and three different concentrations.

4. Who can take part?

Anyone who meets the following criteria can take part:

- healthy women age between 18 and 45 years on the day of screening (initial visit)
- available for 6 months from screening (initial visit)
- able to read and speak English well enough to be able to understand the procedures required and the risks and benefits
- at low risk of becoming HIV positive during the study, and willing to have a HIV test
- willing to undergo a vaginal (internal) examination and a screen for sexually transmitted and vaginal infections
- normal cervical smear within the last 12 months
- if heterosexually active, using an effective method of contraception with their partner (combined oral contraceptive pill; injectable contraceptive; coil; consistent record with condoms if using these; physiological or anatomical sterility in self or partner) from 14 days prior to the first vaccination until 4 months after the last
- willing to undergo urine pregnancy tests prior to each immunisation
- agree, should they be allocated to immunisations in the vagina, not to have a bath or shower for 4 hours after each dose, and not to have sex for 24 hours after each dose, and to use condoms (without spermicide) for one week after each dose
- agree, should they be allocated to immunisations in the vagina or up to nose, not to use any medications or other agents applied via the same route from 24 hours prior to dosing through to the safety assessment 4 weeks later
- agree to refrain from donating blood for three months after the end of their participation in the trial, or longer if necessary
- registered with a GP for at least the past three months and a medical report is received from the GP

It will not be possible to take part if:

- you are pregnant or breast-feeding
- you have a clinical problem that the doctor is concerned will put you at unnecessary risk by participating in this trial
- you have been using drugs that suppress your immune system, such as steroids, or inhaled drugs in preceding 3 months
- you have received certain types of vaccine within 60 days of enrolment
- you have received blood products or immunoglobulin within 4 months of screening
- you are participating in another trial of a medicinal product, or completed one less than 30 days prior to enrolment
- you are not clearly negative in the screening tests for HIV, hepatitis B, hepatitis C or syphilis

5. Do I have to take part?

It is up to you to decide whether or not to take part. If you do decide to take part you will be given this information sheet to keep and be asked to sign a consent form. If you decide to take part you are still free to withdraw at any time and without giving a reason. A decision to withdraw at any time, or a decision not to take part, will not affect the standard of care you receive.

6. What will happen to me if I take part?

You will be in the trial for approximately six months and will attend for at least 10 clinic visits:

- o a screening visit which will take 1-2 hours and will include a physical examination plus an internal examination of the vagina (like taking a smear test) and blood tests
- o three visits at which you will receive vaccines; these will take at least 2 hours as we will ask you to wait to complete a safety check 60 minutes after the immunisation
- o a safety visit within one week of each immunisation
- o three further follow-up visits 12, 16 and 20 weeks after the first immunisation

The safety and follow-up visits should take 15-30 minutes.

Because we don't know which method of giving the vaccine will have the best result we need to make comparisons. Once you have agreed to enter the trial, you will be allocated to one of four treatments with equal chances of each treatment being the one you will receive. This is known as **randomisation**. Allocating treatment this way means that the groups of people getting each treatment should be similar. If there are any differences between how the groups do, it must be due to the treatment. Because the potential vaccine is being given by different routes, participants will know which group they are in, except for those receiving the immunisation in the muscle only. These participants could be given a low dose of vaccine (20 mcg) or a standard dose (100 mcg).

7. What do I have to do?

You will be randomly allocated to receive one of 4 different vaccine regimes:

Group 1: This group will receive **three 0.4mL intramuscular injections of the standard dose (100 mcg)** of vaccine in the upper arm of your choice.

Group 2: This group will receive **three 0.4mL intramuscular injections of the lower dose (20 mcg)** of vaccine in the upper arm of your choice.

Group 3: This group will receive **three intranasal immunisations** where you will be asked to lie or sit with your head tilted back so that gravity pulls the vaccine into each nostril. You will be asked to breathe through your mouth while the vaccine is dripped in at a rate of approximately one drop per nostril every 5 sec. You should remain with your head tilted back for 5 minutes after completion of dosing, and must not blow your nose until 1 hour after dosing. (0.2 mL per nostril)

Group 4: This group will receive **one 0.4mL intramuscular injection of the standard dose (100 mcg)** followed by **two 3mL intravaginal immunisations**. The intravaginal immunisations are delivered in a gel which will be applied using a plastic applicator. The applicator is simply inserted well into the vagina and the plunger pushed in to expel the vaccine. You may self-administer it if you wish.

We will collect three types of samples to measure the immune responses in the laboratory:

- blood samples some of which will be a large volume (up to 3 table-spoons)
- a sample of cervical and vaginal secretions using Weck-Cel® sponges
- a sample of vaginal secretions which will be collected in a device known as the Soft cup, an alternative to tampons which you insert yourself and leave in for at least 1

hour while vaginal secretions collect in the cup (unless you are using a coil in which case vaginal Weck-Cel sampling will be used).

The samples will be processed and sent to various laboratories some of which may be outside the United Kingdom. If once the samples have been analysed there is any remaining they will be destroyed.

8. What are the side-effects of any treatment received when taking part?

You may experience side effects after receiving some of the vaccines in this trial. We expect these may be similar to the side effects that occur after prescribed immunisations. They could include:

- As after any immunisation: fever, chills, general muscle aches (like flu), feeling unusually tired, headache and nausea
- As after immunisations in the muscle: discomfort (itching or irritation in the overlying skin or an ache in the muscle itself), redness and swelling at injection site, fluid filled blisters
- As after immunisations up the nose: discomfort in the nose or throat, runny nose and congestion, nosebleeds
- As after immunisations in the vagina: discomfort (itching or burning in the vagina), discomfort during sex, unusual vaginal discharge, bleeding that is not your usual period

The potential vaccines being tested have only been administered to a small number of women intravaginally. However, products that have been made in a similar way have been given to thousands of individuals without causing any unexpectedly serious side effects.

9. What are the possible disadvantages and risks of taking part?

Because this potential vaccine has only been in a small number of women to date, we do not know very much about the safety. This means that the side effects listed above may be worse than we are expecting, and there could be other side effects that are rarely seen with prescribed immunisations.

It is possible that if the treatment is given to a pregnant woman it will harm the unborn child. This is why we need women to use an effective form of contraception and to have pregnancy tests before each immunisation. Any woman who finds that she has become pregnant while taking part in the study should immediately tell her research doctor.

It is possible that you will have an indeterminate HIV result because of the response you make to the potential vaccine. Standard tests used in UK laboratories can distinguish between HIV infection and an indeterminate HIV test, but you will not be able to donate blood until the indeterminate result has cleared. Insurance companies and occupational health departments may need certification from us to confirm your participation in this trial.

Even if you develop results in HIV tests that suggest that your immune system has responded to the potential vaccine, it will not be possible to tell what effect this will have on your ability to acquire HIV in the future. Although there is a small chance that it could be protective, it is more likely that it will offer you no protection at all, or it could even have an adverse effect on the course and severity on your disease progression if you were to subsequently catch HIV after your participation in the study.

Blood sampling can sometimes cause bruising and soreness of the arms or, very rarely, a blockage of a vein or a small nerve injury which can cause numbness and pain. Normally these problems disappear with time. Some volunteers may faint while the blood is being drawn.

Vaginal examinations and sampling of secretions and cells can feel uncomfortable for some women as can the smear test. Very rarely these procedures might cause slight bleeding known as spotting.

If you insert the vaginal applicator too far it may cause a very small bruise or scratch to the cervix which you probably won't notice, but which may be seen during internal examination.

10. What are the possible benefits of taking part?
You will receive no direct benefit from taking part in the trial but the results may help in the search for an effective HIV vaccine. The combination of different routes is unique and so this trial is at the forefront of vaccine discovery. Many people feel that it is rewarding to make this very personal contribution to science. You will be recompensed for your time and travel, up to a total of £1000.

11. What if new information becomes available?
Sometimes during the course of a research project, new information becomes available that is relevant to the vaccine being studied. If this happens, your research doctor will tell you about it and discuss with you whether you want to continue in the study. If you decide to continue in the study you will be asked to sign an updated consent form.

12. What happens when the research study stops?
If the study is stopped early you will be reimbursed for the travel costs and your time that you have given to the study and the reasons for stopping the trial will be fully explained to you.

13a. What if something goes wrong?
If you feel you have been harmed by taking part in this research project you should discuss this with the clinic team. They will advise you on the likelihood of the vaccine or the trial itself causing the problem. The Sponsor has made arrangements to cover no-fault compensation for harm due to the potential vaccine or trial that could not have been anticipated.

If you are harmed due to someone's negligence then each of the clinical centres taking part has made arrangements to cover compensation due to clinical negligence. You should discuss this with the clinic team, but if this is not possible with

London - SGUL - Head of Joint Research Office should be contacted on 020 8672 9944
Available Monday to Friday between 9am and 5pm.

York - PALS (patient advice and liaison service) York Hospital. York 01904 72 6262.
Available Mon to Fri between 8.30 am and 4.30 pm, or on email pals@york.nhs.uk.]

13b. What if I have a complaint?
If you wish to complain or have any concerns about any aspect of the way you have been approached or treated during the course of this study, you should write to:

York	London
Patient Experience Office<br>York Hospitals NHS Foundation Trust<br>York Hospital<br>Wigginton Road<br>York YO31 8HE	Head of Joint Research Office<br>St George's University of London<br>Cranmer Terrace<br>London<br>SW17 0RE

14. Will my taking part in this study be kept confidential?

If you consent to take part in the research your name and address will be removed from the data before it is sent to the Medical Research Council Clinical Trials Unit where the results will be analysed. Your name will not be disclosed outside the NHS Trust/GP surgery. To ensure the study is properly conducted your clinical notes may be looked at by persons authorised by the study sponsor or the NHS Trust to carry out study monitoring or auditing and by inspectors from regulatory authorities.

If you agree to take part in the study your GP will be contacted to confirm that you are in good health before you can be enrolled; this is a precondition of the trial. If you do participate they will be notified.

15. What will happen to the results of the research study?

We hope that the enrolment will take no more than 6 months. If we manage this, the last visit will take place 12 months after the start. There will be analyses to do, but we would expect to have these finished within 6 months of the last visit. Clinic staff will ask for your contact details so that we can let you know the results either at a seminar, or by email or on the phone.

We hope to publish the results in medical journals, and present them at international conferences. You will not be named in any of these.

16. Who is organising and funding the research?

The research is funded by The Wellcome Trust under the Grand Challenges in Global Health Initiative and the UK HIV Vaccine Consortium.

The trial is sponsored by St George's University of London

The hospital you are attending will receive funds to cover the cost of the laboratory tests and the management of the study including the time of the research nurse and doctor.

17. Who has reviewed the study?

This study has been approved by the South East London 1 Research Ethics Committee which reviewed the study.

18. Contact for Further Information

For further information, please contact: *(you should give the participant a contact point(s) for further information. This can be a doctor and/or nurse involved in the study locally)*

Trial Physician ______________________________

Trial Nurse ______________________________

Thank you for taking part in this study

You will be given a copy of the information sheet and a signed consent form to keep.

APPENDIX 1A: PARTICIPANT INFORMATION SHEET- BOOSTING SUB-STUDY

(To be presented on local headed paper)

PARTICIPANT INFORMATION SHEET

Date and version: 11/06/2012 v2

Acronym of study: MUCOVAC2

1. Study title

A Phase I clinical trial to assess the safety and immunogenicity of three HIV CN54gp140 immunisations administered through the intramuscular, intranasal and intravaginal routes in healthy female volunteers

2. Invitation paragraph

You are being invited to take part in an extension or sub-study to the research study (MUCOVAC2) that you have already agreed to do. We are asking you if you would be willing to be involved in this sub-study as recent animal data has suggested that the immune responses to the vaccine can be improved with having booster doses.

Before you decide it is important for you to understand why the research is being done and what it will involve. Please take time to read the following information carefully and discuss it with others if you wish. Ask us if there is anything that is not clear or if you would like more information. Take time to decide whether or not you wish to take part. Thank you for reading this.

3. What is the purpose of the study?

There is an urgent need to develop methods to prevent HIV infection, given the continuing world-wide epidemic and an estimated 14,000 new infections a day.
Vaccines are products that are given to people in advance to help protect them from developing illnesses caused by particular infections. If successful, they work by stimulating and preparing the body's immune system (the cells and tissues that fight infection) so that if a person is exposed to a specific infection in the future, it can be recognised and cleared before the person becomes unwell.
To date, several potential HIV vaccines have been developed, and three have been assessed in large populations at risk of HIV infection to see if they are able to prevent any infections. Results with the first two were disappointing but the third showed a small benefit. Although the benefit was too small to lead to a successful vaccine, the positive result has given hope, and led to renewed efforts to strengthen potential HIV vaccines.

The aim of this particular study is to assess the safety of a new potential HIV vaccine. We'll also assess how well the vaccine stimulates the body's immune system by testing your blood in the laboratory. However, this study is **not** designed to test whether the vaccine actually provides real life protection against HIV. Even if you participate in this study and your immune system is stimulated by the vaccine, this **does not** mean that you will have been protected against HIV and it will still be possible for you to acquire HIV in the future.

Unlike some other vaccines, the potential vaccine we are testing **does not** contain any active (live) or denatured (killed) whole HIV or any other vector (carrier) viruses. Instead, it consists of a protein (a part known as gp140) that is found on the surface of HIV. Our potential vaccine is similar to some HIV vaccines that have been tested previously, including one used in the trial that showed a small benefit, but the way it will be given is different.
At present, it isn't clear which route of giving an HIV vaccine is best at being able to stimulate the immune system and prevent the virus from being acquired through sex. The vaccine we are testing has previously been shown to be safe when given to a small number of women intravaginally. It has also been given to you and a number of other women by a several different routes, either as an injection in the muscle, in the nose and in the vagina. So far there have been no serious reactions related to the vaccine but the main part of the study is still continuing.

4. Who can take part in this substudy?

Anyone who meets the following criteria can take part:

- Women who have previously received three vaccinations in the main part of the study (either in the nose or as an injection in the muscle) and who are happy to have two further boosters
- Women who are healthy on the day of the booster
- available for a further 3 months from the booster dose
- able to read and speak English well enough to be able to understand the procedures required and the risks and benefits
- at low risk of becoming HIV positive during the study, and willing to have a HIV test
- willing to undergo a vaginal (internal) examination and a screen for sexually transmitted and vaginal infections
- if heterosexually active, using an effective method of contraception with their partner (combined oral contraceptive pill; injectable contraceptive; coil; consistent record with condoms if using these; physiological or anatomical sterility in self or partner) from 14 days prior to the first vaccination until 4 months after the last
- willing to undergo urine pregnancy tests prior to each immunisation
- agree to refrain from donating blood for three months after the end of their participation in the trial, or longer if necessary

It will not be possible to take part if:

- you are pregnant or breast-feeding
- you have a clinical problem that the doctor is concerned will put you at unnecessary risk by participating in this trial
- you have been using drugs that suppress your immune system, such as steroids, or inhaled drugs in preceding 3 months
- you have received certain types of vaccine within 60 days of enrolment
- you have received blood products or immunoglobulin within 4 months of screening
- you are participating in another trial of a medicinal product, or completed one less than 30 days prior to enrolment
- you are not clearly negative in the screening tests for HIV, hepatitis B, hepatitis C or syphilis

5. Do I have to take part?

It is up to you to decide whether or not to take part. If you do decide to take part you will be given this information sheet to keep and be asked to sign a consent form. If you decide to take part you are still free to withdraw at any time and without giving a reason. A decision to withdraw at any time, or a decision not to take part, will not affect the standard of care you receive.

6. What will happen to me if I take part?

You will be in this booster sub-trial for approximately three months and will attend for at least 5 clinic visits:

- you may require a repeat screening visit which will take 1-2 hours and will include a physical examination plus an internal examination of the vagina (like taking a smear test) and blood tests
- two visits at which you will receive vaccines; these will take at least 2 hours as we will ask you to wait to complete a safety check 60 minutes after the immunisation
- a safety visit within one week of each immunisation
- one to two further follow-up visits 4 and 8 weeks after the first immunisation

The safety and follow-up visits should take 15-30 minutes.

7. What do I have to do?

You will receive **two 0.4mL intramuscular injections of the standard dose (100 mcg)** of vaccine in the upper arm of your choice.

We will collect three types of samples to measure the immune responses in the laboratory:

- blood samples some of which will be a large volume (up to 3 table-spoons)
- a sample of cervical and vaginal secretions using Weck-Cel® sponges
- a sample of vaginal secretions which will be collected in a device known as the Soft cup, an alternative to tampons which you insert yourself and leave in for at least 1 hour while vaginal secretions collect in the cup (unless you are using a coil in which case vaginal Weck-Cel sampling will be used).

The samples will be processed and sent to various laboratories some of which may be outside the United Kingdom. If once the samples have been analysed there is any remaining they will be destroyed.

8. What are the side-effects of any treatment received when taking part?

You may experience side effects after receiving some of the vaccines in this trial. We expect these may be similar to the side effects that occur after prescribed immunisations. They could include:

- As after any immunisation: fever, chills, general muscle aches (like flu), feeling unusually tired, headache and nausea
- As after immunisations in the muscle: discomfort (itching or irritation in the overlying skin or an ache in the muscle itself), redness and swelling at injection site, fluid filled blisters

The potential vaccines being tested have only been administered to a small number of women intravaginally. There are also a number of women in the current trial (MUCOVAC2) who have been given the vaccine in their arm, nose or vagina, although not all women have completed all visits. However, products that have been made in a similar way have been given to thousands of individuals without causing any unexpectedly serious side effects.

9. What are the possible disadvantages and risks of taking part?

Because this potential vaccine has only been in a small number of women to date, we do not know very much about the safety. This means that the side effects listed above may be worse than we are expecting, and there could be other side effects that are rarely seen with prescribed immunisations.

It is possible that if the treatment is given to a pregnant woman it will harm the unborn child. This is why we need women to use an effective form of contraception and to have pregnancy tests before each immunisation. Any woman who finds that she has become pregnant while taking part in the study should immediately tell her research doctor.

It is possible that you will have an indeterminate HIV result because of the response you make to the potential vaccine. Standard tests used in UK laboratories can distinguish between HIV infection and an indeterminate HIV test, but you will not be able to donate blood until the indeterminate result has cleared. Insurance companies and occupational health departments may need certification from us to confirm your participation in this trial.

Even if you develop results in HIV tests that suggest that your immune system has responded to the potential vaccine, it will not be possible to tell what effect this will have on your ability to acquire HIV in the future. Although there is a small chance that it could be protective, it is more likely that it will offer you no protection at all, or it could even have an adverse effect on the course and severity on your disease progression if you were to subsequently catch HIV after your participation in the study.

Blood sampling can sometimes cause bruising and soreness of the arms or, very rarely, a blockage of a vein or a small nerve injury which can cause numbness and pain. Normally these problems disappear with time. Some volunteers may faint while the blood is being drawn.

Vaginal examinations and sampling of secretions and cells can feel uncomfortable for some women as can the smear test. Very rarely these procedures might cause slight bleeding known as spotting.

If you insert the vaginal applicator too far it may cause a very small bruise or scratch to the cervix which you probably won't notice, but which may be seen during internal examination.

10. What are the possible benefits of taking part?

You will receive no direct benefit from taking part in the trial but the results may help in the search for an effective HIV vaccine. The combination of different routes is unique and so this trial is at the forefront of vaccine discovery. Many people feel that it is rewarding to make this very personal contribution to science. You will be recompensed for your time and travel, up to a total of £100 per visit.

11. What if new information becomes available?

Sometimes during the course of a research project, new information becomes available that is relevant to the vaccine being studied. If this happens, your research doctor will tell you about it and discuss with you whether you want to continue in the study. If you decide to continue in the study you will be asked to sign an updated consent form.

12. What happens when the research study stops?

If the study is stopped early you will be reimbursed for the travel costs and your time that you have given to the study and the reasons for stopping the trial will be fully explained to you.

13a. What if something goes wrong?

If you feel you have been harmed by taking part in this research project you should discuss this with the clinic team. They will advise you on the likelihood of the vaccine or the trial itself causing the problem. The Sponsor has made arrangements to cover no-fault compensation for harm due to the potential vaccine or trial that could not have been anticipated.

If you are harmed due to someone's negligence then each of the clinical centres taking part has made arrangements to cover compensation due to clinical negligence. You should discuss this with the clinic team, but if this is not possible with

London - SGUL - Head of Joint Research Office should be contacted on 020 8672 9944
Available Monday to Friday between 9am and 5pm.

York - PALS (patient advice and liaison service) York Hospital. York 01904 72 6262.
Available Mon to Fri between 8.30 am and 4.30 pm, or on email pals@york.nhs.uk.

13b. What if I have a complaint?

If you wish to complain or have any concerns about any aspect of the way you have been approached or treated during the course of this study, you should write to:

York	London
Patient Experience Office<br>York Hospitals NHS Foundation Trust<br>York Hospital<br>Wigginton Road<br>York YO31 8HE	Head of Joint Research Office<br>St George's University of London<br>Cranmer Terrace<br>London<br>SW17 0RE

14. Will my taking part in this study be kept confidential?

If you consent to take part in the research your name and address will be removed from the data before it is sent to the Medical Research Council Clinical Trials Unit where the results will be analysed. Your name will not be disclosed outside the NHS Trust/GP surgery. To ensure the study is properly conducted your clinical notes may be looked at by persons authorised by the study sponsor or the NHS Trust to carry out study monitoring or auditing and by inspectors from regulatory authorities.

If you agree to take part in the study your GP will be contacted to confirm that you are in good health before you can be enrolled; this is a precondition of the trial. If you do participate they will be notified.

15. What will happen to the results of the research study?

We hope that once all visits have been completed for all volunteers (expected early in 2013) we will complete our analyses and reports within about 6 months of the last visit. Clinic staff will ask for your contact details so that we can let you know the results either at a seminar, or by email or on the phone.

We hope to publish the results in medical journals, and present them at international conferences. You will not be named in any of these.

16. Who is organising and funding the research?

The research is funded by The Wellcome Trust under the Grand Challenges in Global Health Initiative and the UK HIV Vaccine Consortium.

The trial is sponsored by St George's University of London

The hospital you are attending will receive funds to cover the cost of the laboratory tests and the management of the study including the time of the research nurse and doctor.

17. Who has reviewed the study?

This study has been approved by the South East London 1 Research Ethics Committee which reviewed the study.

18. Contact for Further Information

For further information, please contact: *(you should give the participant a contact point(s) for further information. This can be a doctor and/or nurse involved in the study locally)*

Trial Physician ______________________________

Trial Nurse ______________________________

Thank you for taking part in this study

You will be given a copy of the information sheet and a signed consent form to keep.

APPENDIX 2: CONSENT FORM

(To be presented on local headed paper)

Consent Form

Trial Number: xxx

Date and version: 16/01/2012 v1.5

Acronym and title of study: MUCOVAC2, A Phase I clinical trial to assess the safety and immunogenicity of three HIV CN54gp140 immunisations administered through the intramuscular, intranasal and intravaginal routes in healthy female volunteers

Please initial box to agree

1. I confirm that I have read and understand the information sheet dated 16/01/2012 (version 1.5) for the above study and have had the opportunity to ask questions. ☐

2. I understand that my participation is voluntary and that I am free to withdraw at any time, without giving any reason, without my medical care or legal rights being affected. ☐

3. I understand that sections of any of my medical notes may be looked at by responsible individuals involved in the running of the trial or from regulatory authorities where it is relevant to my taking part in research, and that I may be followed up through usual NHS mechanisms (e.g. Office for National Statistics). I give permission for these individuals to have access to my records. ☐

4. I agree to my GP being contacted and being asked to provide a report ☐

5. I agree to be entered onto a database (TOPS) as a measure to prevent over volunteering ☐

6. I give permission for my stored samples to be made available for research where the samples would be stored appropriately and some may be sent outside the UK ☐

7. I agree to take part in the above study. ☐

____________________	____________	________________
Name of participant	Date	Signature
____________________	____________	________________
Investigator or person designated by Investigator	Date	Signature

3 copies: 1 for participant, 1 for investigator/designee, 1 to be kept with hospital notes

APPENDIX 2A: CONSENT FORM FOR BOOSTING SUBSTUDY

(To be presented on local headed paper)

Consent Form

Trial Number: xxx

Date and version: 11/06/2012 v2

Acronym and title of study: MUCOVAC2, A Phase I clinical trial to assess the safety and immunogenicity of three HIV CN54gp140 immunisations administered through the intramuscular, intranasal and intravaginal routes in healthy female volunteers

Please initial box to agree

1. I confirm that I have read and understand the information sheet dated 11/06/2012 (version 2) for the above study and have had the opportunity to ask questions. ☐

2. I understand that my participation is voluntary and that I am free to withdraw at any time, without giving any reason, without my medical care or legal rights being affected. ☐

3. I understand that sections of any of my medical notes may be looked at by responsible individuals involved in the running of the trial or from regulatory authorities where it is relevant to my taking part in research, and that I may be followed up through usual NHS mechanisms (e.g. Office for National Statistics). I give permission for these individuals to have access to my records. ☐

4. I agree to be entered onto a database (TOPS) as a measure to prevent over volunteering ☐

5 I give permission for my stored samples to be made available for research where the samples would be stored appropriately and some may be sent outside the UK ☐

6. I agree to take part in the above study. ☐

____________________	____________________	____________________
Name of participant	Date	Signature
____________________	____________________	____________________
Investigator or person designated by Investigator	Date	Signature

3 copies: 1 for participant, 1 for investigator/designee, 1 to be kept with hospital notes

APPENDIX 3a: GP LETTER (pre-trial)

(to be printed on local headed paper)

Version 1.5 dated 16/01/2012

Doctor Address

Date

Dear Dr ____________

Re: MUCOVAC 2- A Phase I clinical trial to assess the safety and immunogenicity of three HIV CN54gp140 immunisations administered through the intramuscular, intranasal and intravaginal routes in healthy female volunteers (EudraCT number: 2010-019103-27)

We are writing to you about <<participant name>>, (<<date of birth: dd/mmm/yyyy>>), who we understand is your patient, and who has consented to be entered into the MUCOVAC2 trial. This is a Phase I, randomised, two-centre, exploratory trial of immunisations with a candidate HIV vaccine (CN54gp140), administered in four different regimens.

Please find enclosed a participant information sheet and the eligibility criteria for this trial.

In order for us to proceed with enrolling your patient into this study, we are seeking confirmation from you, her GP, that a) she has not recently participated in any other clinical trials as far as you are aware, b) she has no significant ongoing illness and c) there is no reason that you feel your patient should not be entered into the study. We therefore enclose a very short questionnaire for you to complete in order to confirm these details. We also enclose a copy of the signed consent form, which indicates that the participant has provided consent for us to contact you to request this information.

I would be grateful if you could complete this form as soon as feasible and return it in the enclosed prepaid envelope (to the above address). Upon receipt of the completed form we will send you an honorarium of £40 in the form of a cheque.

You will be kept up to date with your patient's progress but if you have any concerns or questions in the meantime, please do not hesitate to contact me.

Yours sincerely,

(Delete as appropriate)

Dr Catherine Cosgrove
Chief Investigator

Prof Charles JN Lacey/Dr Georgina Morris
Principal Investigator

MUCOVAC 2: Eligibility Criteria

Participant inclusion criteria

1. women aged between 18 and 45 years on the day of screening
2. available for follow-up for the duration of the study (6 months from screening)
3. willing and able to give written informed consent
4. at low risk of HIV and willing to remain so for the duration of the study defined as:
   - no history of injecting drug use in the previous ten years
   - no gonorrhoea or syphilis in the last six months
   - no high risk partner (e.g. injecting drug use, HIV positive partner) either currently or within the past six months
   - no unprotected anal intercourse in the last six months, outside a relationship with a regular partner known to be HIV negative
   - no unprotected vaginal intercourse in the last six months outside a relationship with a regular known/presumed HIV negative partner
5. willing to undergo a HIV test
6. willing to undergo a vaginal examination and genital infection screen
7. Normal cervical smear within 12 months of screening
8. if heterosexually active female, using an effective method of contraception with partner (combined oral contraceptive pill; injectable or implanted contraceptive; any IUCD/IUS; consistent record with condoms if using these; physiological or anatomical sterility in self or partner) from 14 days prior to the first vaccination until 4 months after the last, and willing to undergo urine pregnancy tests prior to each vaccination
9. agree, should they be allocated to IVAG immunisations, to abstain from having a bath or shower for 4 hrs after each dose, and from sex for 24 hrs after each dose, and to use condoms (without spermicide) for one week after each dose
10. agree, should they be allocated to IVAG or IN immunisations to abstain from medications or other agents that are applied via the same route from 24 hrs prior to dosing through to the safety assessment 4 weeks later
11. agree to abstain from donating blood for three months after the end of their participation in the trial, or longer if necessary
12. registered with a GP for at least the past three months
13. satisfactory response received from GP before randomisation

Participant exclusion criteria

1. pregnant or breast-feeding
2. clinically relevant abnormality on history or examination including
   - history of grand-mal epilepsy
   - cranial nerve palsies
   - severe eczema
   - severe epistaxis
   - liver disease with inadequate hepatic function
   - haematological, metabolic, gastrointestinal or cardio-pulmonary disorders
   - uncontrolled infection; autoimmune disease, immunodeficiency or use of immunosuppressives in preceding 3 months
   - using inhaled cortico-steroids and IN medications
3. known or suspected history of clinically relevant cervico-vaginal disease, malignancy or abnormality, which in the opinion of the investigator might interfere with IVAG

dose administration

4. known or suspected history of clinically relevant nasal surgery, injury or condition likely to require regular intranasal medication, which in the opinion of the investigator might interfere with IN dose administration
5. known hypersensitivity to any component of the vaccine formulations used in this trial, or a seafood allergy or have severe or multiple allergies to drugs or pharmaceutical agents
6. history of severe local or general reaction to vaccination defined as
    a. **local**: extensive, indurated redness and swelling involving most of the antero-lateral thigh or the major circumference of the arm, not resolving within 72 hours
    b. **general**: fever >= 39.5°C within 48 hours; anaphylaxis; bronchospasm; laryngeal oedema; collapse; convulsions or encephalopathy within 72 hours
7. receipt of live attenuated vaccine within 60 days or other vaccine within 14 days of enrolment
8. receipt of an experimental vaccine containing HIV envelope proteins at any time in the past
9. receipt of blood products or immunoglobin within 4 months of screening
10. participation in another trial of a medicinal product, completed less than 30 days prior to enrolment
11. HIV 1/2 positive or indeterminate on screening
12. positive for hepatitis B surface antigen, hepatitis C antibody or serology indicating active syphilis requiring treatment
13. Grade 1 or above routine laboratory parameters on screening

(Hyperbilirubinemia to be considered an exclusion criterion only when confirmed to be conjugated bilirubinaemia)

14. unable to read and speak English to a fluency level adequate for the full comprehension of procedures required in participation and consent.
15. unlikely to comply with protocol

Confirmation of eligibility for participation in ‘MUCOVAC2’ clinical trial

Version 1.4 dated 27/09/2011

PATIENT IDENTIFICATION

Surname: ______________________

First Name: ______________________

Date of Birth: [DAY | MONTH | YEAR]

PATIENT HISTORY

Is this patient currently registered with your practice?	Yes ☐	No ☐
Has this patient been registered with you for at least 3 months?	Yes ☐	No ☐
If ‘no’ please indicate the date of registration with your practice:	DAY / MONTH / YEAR	

Please answer the following to the best of your knowledge.
<u>If you answer ‘yes’ to any of the following questions, please provide details in the ‘comments’ section below</u>

Has the patient ever been involved in another clinical research study? (please give details of dates of involvement/nature of study product, if known)	Yes ☐	No ☐

Do they have any history of the following conditions?

• grand-mal epilepsy	Yes ☐	No ☐
• cranial nerve palsies	Yes ☐	No ☐
• severe eczema	Yes ☐	No ☐
• severe epistaxis	Yes ☐	No ☐
• liver disease (with decreased hepatic function)	Yes ☐	No ☐
• haematological, metabolic, gastrointestinal or cardio-pulmonary problems	Yes ☐	No ☐
• Uncontrolled infection, autoimmune disease or immunodeficiency	Yes ☐	No ☐

Has the patient ever received treatment for cervical intraepithelial neoplasia (CIN) or a gynaecological malignancy	Yes ☐	No ☐
Do they have any history of nasal surgery or injury or a condition likely to require the regular use of intranasal medications?	Yes ☐	No ☐
Has the patient been prescribed any medication in the past 12 months? *(please give details of medication, dose and dates prescribed/used)*	Yes ☐	No ☐
In particular, have they received blood products, immunoglobulin, immunosuppressants, oral or inhaled corticosteroids within the past 4 months?	Yes ☐	No ☐
Has the patient received any immunisations within the past 60 days? *(please provide dates, name and type of vaccine)*	Yes ☐	No ☐
Any known drug or other allergies?	Yes ☐	No ☐
Any history of any severe adverse reactions to any vaccine, drug or pharmaceutical product?	Yes ☐	No ☐
When was her last cervical cytology smear?	___/____/____	
Was this	Normal ☐	Abnormal ☐ (detail below)

COMMENTS/DETAILS: *Please add any relevant comments/details below. Please continue over-page or on another sheet if necessary.*

I hereby declare that the information provided is accurate and up to date, to the best of my knowledge.

Signature	Name (Printed)	Date (DAY / MONTH / YEAR)

APPENDIX 3b: GP LETTER

(To be presented on local headed paper) Date and version: 16/01/2012 v 1.5

Doctor Address

Date

Trial Number: xxx

Acronym and title of study: MUCOVAC2, A Phase I clinical trial to assess the safety and immunogenicity of three HIV CN54gp140 immunisations administered through the intramuscular, intranasal and intravaginal routes in healthy female volunteers

Dear Dr ____________

Your patient, ________________ (date of birth dd/mmm/yyyy), has been entered to the above trial.

Your patient has been allocated to receive:

group 1	☐	3 20µg Intramuscular
group 2	☐	3 100µg Intramuscular
group 3	☐	3 100µg Intranasal
group 4	☐	1 100µg Intramuscular, 2 500µg intravaginal

You will be kept up to date with your patient's progress but if you have any concerns or questions regarding this study please contact the responsible doctor:

Dr ______________________________ at ___________________________(Hospital)

Tel: _______________________________

Kind regards,

Name
<u>Position</u>

APPENDIX 3c: GP LETTER BOOSTING PHASE

(To be presented on local headed paper) Date and version: 11/06/2012 v2

Doctor Address

Date

Trial Number: xxx

Acronym and title of study: MUCOVAC2, A Phase I clinical trial to assess the safety and immunogenicity of three HIV CN54gp140 immunisations administered through the intramuscular, intranasal and intravaginal routes in healthy female volunteers

Dear Dr ____________

Your patient, ________________ (date of birth dd/mmm/yyyy), was entered into and completed the main part of the trial. Evidence has since come to light to suggest that giving 2 extra vaccinations will increase the immune responses.

As your patient was allocated and received 3 100µg Intramuscular or 100µg Intranasal satisfactorily she has been offered and consented to 2 further 100µg Intramuscular to be given 4 weeks apart starting 12 to 24 weeks after her initial priming vaccinations.

You will be kept up to date with your patient's progress but if you have any concerns or questions regarding this study please contact the responsible doctor:

Dr ______________________________ at ___________________________(Hospital)

Tel: ______________________________

Kind regards,

Name
Position

APPENDIX 4: TOXICITY TABLE

Based on systems in use at the MRC CTU, IAVI and NIH Division of AIDS

Abbreviations:

ULN	Upper Limit of Normal	LLN	Lower Limit of Normal
R_x	Therapy	Req	Required
Mod	Moderate	IV	Intravenous
ADL	Activities of Daily Living	Dec	Decreased

For other events not specified in the tables below the severity will be determined according to the CTCAE table version 4.02 http://www.acrin.org/Portals/0/Administration/Regulatory/CTCAE_4.02_2009-09-15_QuickReference_5x7.pdf

LABORATORY PARAMETERS

PARAMETER	GRADE 1<br>MILD	GRADE 2<br>MODERATE	GRADE 3<br>SEVERE	GRADE 4<br>EXTREME
**HAEMATOLOGY**				
Hb	10.0-10.9 g/dL	9.0-9.9 g/dL	7.0-8.9 g/dL	<7.0 g/dL
White Blood Count	13.0 – 14.9 $x10^9$/l<br>or 2 – 2.5 $x10^9$/l	15.0 – 19.9 $x10^9$/l<br>or 1.5 – <2.0 $x10^9$/l	20.0 – 29.9 $x10^9$/l<br>or 1 – <1.5 $x10^9$/l	≥30.0<br>or <1.0 $x10^9$/l
Absolute Neutrophils	1.3-1.0 $x10^9$/l	<1.0-≥0.75 $x10^9$/l	<0.75-≥0.5 $x10^9$/l	<0.5 $x10^9$/l
Percent neutrophils	>80%	90%	≥95%	---
Lymphocytes	0.7- 0.899 $x10^9$/l	0.5-0.699 $x10^9$/l	0.35-0.499 $x10^9$/l	<0.35 $x10^9$/l
Platelets	100 –124.999 x 10^9/l	50 – 99.999 x 10^9/l	25 – 49.999 $x10^9$/l	<25.0 $x10^9$/l
CD4 Count	300-400/mm^3	<300mm^3	<200/mm^3	<100/mm^3
**BIOCHEMISTRY**				
Potassium<br>Hyperkalemia<br>Hypokalemia	 5.6 – 6.0 meq/L<br>3.0 – 3.4 meq/L	 6.1-6.5 meq/L<br>2.5 – 2.9 meq/L	 6.6-7.0 meq/L<br>2.0– 2.4 meq/L	 >7.0 meq/L<br><2.0 meq/L
Bilirubin<br>Hyperbilirubinemia	 >1.25 – 2.0 x ULN	 >2.0 – 2.5 x ULN	 >2.5 – 5 x ULN	 >5 x ULN
Glucose<br>Hypoglycaemia<br>Hyperglycaemia nonfasting; no prior diabetes	2.3-2.4 mmol/l<br>7.0-10.0 mmol/l	2.1-2.2 mmol/l<br>10.1-15.0 mmol/l	1.5-2.0 mmol/l<br>15.1-25.0 mmol/l	<1.5 mmol/l<br>>25.0 mmol/l
Transaminases<br>AST (SGOT)<br>ALT (SGPT)<br>GGT<br>Alk Phos	 1.25 – 2.5 x ULN<br>1.25 – 2.5 x ULN<br>1.25 – 2.5 x ULN<br>1.25 – 2.5 x ULN	 >2.5 – 5.0 x ULN<br>>2.5 – 5.0 x ULN<br>>2.5 – 5.0 x ULN<br>>2.5 – 5.0 x ULN	 >5.0 – 10.0 x ULN<br>>5.0 – 10.0 x ULN<br>>5.0 – 10.0 x ULN<br>>5.0 – 10.0 x ULN	 > 10.0 x ULN<br>> 10.0 x ULN<br>> 10.0 x ULN<br>> 10.0 x ULN
Amylase	>1.0 – 1.5 x ULN	>1.5 – 2.0 x ULN	>2.0 – 5.0 x ULN	>5.0 x ULN
Creatinine	130-180µmol/l	181-360µmol/l	361-720µmol/l	>720µmol/l
**URINALYSIS**				
Proteinuria:<br>24 hour urine	200 mg - 1 g loss/day OR <0.3% OR <3 g/l	1 – 2 g loss/day OR 0.3 – 1.0% OR 3 - 10 g/l	2 – 3.5 g loss/day OR<br>>1.0% OR > 10 g/l	Nephrotic syndrome OR >3.5 g loss/day
Haematuria	Microscopic only ≤10 RBC/HPF	>10 RBC/HPF	Gross, with or without clots OR RBC casts	Obstructive OR transfusion req

SOLICITED VACCINE REACTIONS

**GENERAL**				
Fever<br>Oral>12 hours	37.7 - 38.9°C<br>(100.0 – 101.5°F)	39.0 – 39.7°C<br>(101.6 – 102.9°F)	39.8 – 40.5°C<br>(103 - 105°F)	>40.5°C (105°F)<br>OR max temp of >105°F
Chills/rigors	Mild hot/cold flush requires blanket or occasional aspirin/paracetamol	Limiting daily activity >6 hours, or need regular aspirin/paracetamol	Uncontrollable shaking, treatment from doctor needed	Hospitalisation
Malaise/abnormal tiredness	Normal activity reduced – not bad enough to go to bed	Fatigue such that ½ day in bed for 1 or 2 days	Fatigue such that in bed all day or ½ day for more than 2 days	Hospitalisation
General (all over) muscle aches and pains	No limitation of activity	Muscle tenderness, aches/pains limiting activity e.g. difficulty climbing stairs	Severe limitation e.g. can't climb stairs	Hospitalisation
Headache	No treatment or responds to paracetamol like treatment	Regular paracetamol like treatment needed	Regular strong painkillers needed	Hospitalisation
Nausea	Intake maintained	Intake reduced less than 3 days	Minimal intake 3 days or more	Hospitalisation
**CUTANEOUS**				
Discomfort/pain in injected muscle (including ache) or overlying skin	Mild itch or ache that responds to paracetamol like treatment, if needed	Pain requiring regular paracetamol like treatment	Pain requiring regular strong painkillers	Hospitalisation
Immediate reactions (within 6 hours of injection)	Symptoms of irritation locally (usually itching at the injection site)<br>OR<br>Erythema +/- swelling at the injection site		Laryngeal oedema insufficient to require intubation; diarrhoea insufficient to require IV fluids, or asthma insufficient to require hospitalisation<br>OR<br>Urticaria, angio-oedema<br>OR<br>Generalised pruritus	Anaphylactic shock
Erythema at injection site	Erythema up to and including 50% of baseline arm circumference<br>OR<br>Symptoms of irritation that are easily tolerated and do not require repeated medication<br>OR<br>Both	Erythema greater than 50% of the arm circumference at baseline<br>With or without<br>Symptoms of irritation that do not require repeated medication<br>OR<br>Symptoms of irritation that require repeated medication AND erythema up to and including 50%	Erythema greater than 50% of the arm circumference at baseline AND symptoms of irritation requiring repeated medication	Hospitalisation
Blistering or ulceration at injection site	Fluid filled vesicles or superficial disruption of epithelium covering an area < 1cm	Fluid filled vesicles or superficial disruption of epithelium, area 1 - 2cm<br>OR<br>Blood filled vesicles<br>OR<br>Full thickness disruption of epithelium healed within 2 weeks	Full thickness disruption of epithelium not healed within 2 weeks	**Necrosis**

	Soft swelling – local Swelling <25% of arm	Soft swelling – local Swelling 25-50%of arm	Soft swelling – local Swelling >50% of arm<br>Or<br>Induration/hardened swelling<br>(when considered by the clinician to be associated with a process arising in the muscle)	
**NASAL**				
Discomfort/pain in nose or throat	Mild itch or discomfort that responds to paracetamol like treatment, if needed	Pain requiring regular paracetamol like treatment	Pain requiring regular strong painkillers	Hospitalisation
Congestion/blockage	Mild congestion responds to occasional anti-histamine	Regular anti-histamine needed to control congestion	Complete blockage of one or more nostril despite medication for >3days	
Discharge	Mild runny nose, treatment not needed	Moderate runny nose for which regular treatment is needed	Persistent bloody discharge	
Bleeding	Mild epistaxis, packing not indicated	Moderate epistaxis, medical intervention indicated	Transfusion/ radiologic/ endoscopic/operative intervention	
**VAGINAL**				
Discomfort (itching/burning)/pain	Mild itch or burning sensation that responds to paracetamol like treatment, if needed	Pain requiring regular paracetamol like treatment	Pain requiring regular strong painkillers	Hospitalisation
Pain during sexual intercourse	Pain causing no or minimal interference with sexual function	Pain causing more than minimal interference with sexual function		
Abnormal/unusual discharge	Mild discharge, regular sanitary protection not required	Moderate discharge requiring regular sanitary protection or medical intervention	Persistent bloody discharge	
Non-menstrual bleeding	Light ≤ 7 days	Light >7days or heavy ≤ 4days	Heavy > 4 days or profuse enough to require bed rest or transfusion ≤2units	Hospitalisation or transfusion >2units
Epithelial disruption (sores or ulcers)	≤ 1 swab-tip*	> 1 ≤ 4 swab-tips*	> 4 swab-tips*	Hospitalisation
Erythema (redness)	Local/diffuse mild redness<br>Or<br>Local marked redness	Diffuse marked redness with symptoms that are easily tolerated	Diffuse marked redness with Grade 2 or above discomfort	Hospitalisation
Oedema		Oedema without sloughing of the epithelial cells	Oedema with sloughing of the epithelial cells	

***swab-tip is 5 x 10mm**

APPENDIX 5: DIARY CARD

See overleaf

Please put in grade, including 0 if no symptoms and measure temperature in °C and maximum width of skin lesion in cm

	12 hrs	Day 1	Day 2	Day 3	Day 4	Day 5	Day 6	Day 7
**Date**								
GENERAL SYMPTOMS								
**Temperature °C**								
**Chills/shaking**								
**Malaise/abnormal tiredness**								
**General (all over) muscle aches**								
**Headache**								
**Nausea/feeling sick**								
**SKIN SYMPTOMS**								
**Discomfort/pain at injection site**								
**Redness/discolouration (cm)**								
**Fluid/blood filled blisters (cm)**								
**Soft swelling (cm)**								
**Hard swelling in skin surface at or close to site (cm)**								
**NASAL SYMPTOMS**								
**Discomfort/pain in nose or throat**								
**Congestion/blockage**								
**Discharge including bloodstained**								
**Bleeding**								
**VAGINAL SYMPTOMS**								
**Discomfort/pain in vagina**								
**Pain during sexual intercourse**								
**Abnormal/unusual discharge including bloodstained**								
**Bleeding not related to period**								

Medication – list all medications and total daily dose

	Dose	Dose	Dose	Dose	Dose	Dose	Dose	Dose
**1**								
**2**								
**3**								
**4**								

COMMENTS

Guide for grading reactions

	Grade 1 (mild)	Grade 2 (moderate)	Grade 3 (severe)	Grade 4 (extreme)
GENERAL				
Chills/shaking	*Mild hot/cold flush requires blanket or occasional aspirin/paracetamol*	*Limiting daily activity more than 6 hours, or need regular aspirin/paracetamol*	*Uncontrollable shaking, treatment from doctor needed*	*Requires a stay in hospital*
Malaise/abnormal tiredness	*Normal activity reduced – not bad enough to go to bed*	*Fatigue such that ½ day in bed for 1 or 2 days*	*Fatigue such that in bed all day or ½ day for more than 2 days*	*Requires a stay in hospital*
General (all over) muscle aches and pains	*No limitation of activity*	*Muscle tenderness, aches/pains limiting activity e.g. difficulty climbing stairs*	*Severe limitation e.g. can't climb stairs*	*Requires a stay in hospital*
Headache	*No treatment or responds to paracetamol like treatment*	*Regular paracetamol like treatment needed*	*Regular strong painkillers needed*	*Requires a stay in hospital*
Nausea	*Able to eat & drink normally*	*Eating & drinking reduced less than 3 days*	*Eating & drinking very little for 3 days or more*	*Requires a stay in hospital*
SKIN				
Discomfort/pain in injected muscle (including ache) or overlying skin	*Mild itch or ache that responds to paracetamol like treatment, if needed*	*Pain requiring regular paracetamol like treatment*	*Pain requiring regular strong painkillers*	*Requires a stay in hospital*
NASAL				
Discomfort/pain in nose or throat	*Mild itch or discomfort that responds to paracetamol like treatment, if needed*	*Pain requiring regular paracetamol like treatment*	*Pain requiring regular strong painkillers*	*Requires a stay in hospital*
Congestion/blockage	*Mild congestion responds to occasional anti-histamine*	*Regular anti-histamine needed to control congestion*	*Complete blockage of one or more nostril despite medication for more than 3 days*	
Discharge	*Mild runny nose, treatment not needed*	*Moderate congestion for which regular treatment needed*	*Persistent bloody discharge*	
Bleeding	*Mild nose bleed stops by itself*	*Moderate nosebleed needing visit to doctor or nurse*	*Sever nose bleed requiring specialist treatment at hospital*	
VAGINAL				
Discomfort (itching/burning)/pain	*Mild itch or burning sensation that responds to paracetamol like treatment, if needed*	*Pain requiring regular paracetamol like treatment*	*Pain requiring regular strong painkillers*	*Requires a stay in hospital*
Pain during sexual intercourse	*Pain causing no or minimal interference with sexual function*	*Pain causing more than minimal interference with sexual function*		
Abnormal/unusual discharge	*Mild discharge, regular sanitary protection not required*	*Moderate discharge requiring regular sanitary protection or medical intervention*	*Persistent bloody discharge*	
Non-menstrual bleeding	*Light lasting 7 days or less*	*Light lasting more than 7days or heavy for 4days or less*	*Heavy for more than 4 days or profuse enough to require bed rest or transfusion of 1 or 2 units*	*Requires a stay in hospital or transfusion of more than 2 units*

Diary

MUCOVAC2

**Trial number**	
**Vaccination number**	**1 2 3 4 5**<br>Please circle as appropriate
**Date of vaccination**	

**Contact details:**

- **Complete at approximately the same time every day**
- **Take temperature with the thermometer provide and record in ^{o}C**
- **Record the maximum grade for reactions using the table as a guide**
- **Add any comments regarding other symptoms, please include dates**
- **Record any medication taken including over the counter medicines and anything taken to relieve local reactions**
- **Please contact a member of the trials staff as soon as possible if you experience any symptoms of grade 3 or 4 or have any concerns**
- **When the diary card is complete, please return it to a member of trials staff at your next visit**

Mucovac2 CRF v1.2_11Jun2012
