# Supplementary material for: Comparative Immunogenicity of HIV-1 gp140 Vaccine Delivered by Parenteral, and Mucosal Routes in Female Volunteers; MUCOVAC2, A Randomized Two Centre Study
Source: PLoS One. 2016 May 9;11(5):e0152038. doi: 10.1371/journal.pone.0152038 (PMC4861263; doi:10.1371/journal.pone.0152038)
Supplement: S1 Table — (DOCX) [file pone.0152038.s006.docx]

**S1 Table: Cervico-vaginal specific IgG responses at Week 12 and corresponding serum responses.**

|  | **Baseline**  **(µg/ml)** | **Week 5**  **(µg/ml)** | **Week 12**  **(µg/ml)** |
| --- | --- | --- | --- |
| Softcup Total IgG | 321.6  (9.12–3989.0) | 318.8  (57.29–16417.0) | 405.0  (115.6–3037.0) |
| Softcup Total IgA | 109.6  (6.81–654.3) | 98.95  (12.64–1178.0) | 119.7  (7.52–2666.0) |
| Weck-Cel Cervical os Total IgG | 49.96  (2.73–454.8) | 35.89  (4.01–239.7) | 33.61  (5.51–310.0) |
| Weck-Cel Cervical os  Total IgA | 22.10  (0.010–206.3) | 28.89  (0.010–141.9) | 37.13  (0.010–131.0) |
| Weck-Cel Vaginal  Total IgG | 8.41  (0.01–86.79) | 9.36  (1.84–227.9) | 10.62  (0.01–70.33) |
| Weck-Cel Vaginal  Total IgA | 1.60  ( 0.01–28.44) | 1.257  (0.01–80.01) | 2.04  (0.01–52.56) |

**^Note:^** ^n/a= not available, specific activity % calculated as (µg CN54specificIg/µg Total Ig) x100^
