# Supplementary material for: Comparative Immunogenicity of HIV-1 gp140 Vaccine Delivered by Parenteral, and Mucosal Routes in Female Volunteers; MUCOVAC2, A Randomized Two Centre Study
Source: PLoS One. 2016 May 9;11(5):e0152038. doi: 10.1371/journal.pone.0152038 (PMC4861263; doi:10.1371/journal.pone.0152038)
Supplement: S2 Table — (DOCX) [file pone.0152038.s007.docx]

**S2 Table:**  **Total IgG and IgA levels (combined data from all groups) detected in cervico-vaginal secretions collected by Softcup or Weck-Cel sampling.**

| **ID** | **Group** | **Serum IgG** | **Softcup** | | **Softcup Specific activity (%)** | **Weck Cel  (Cervical OS)** | | **Weck Cel  (Vaginal Vault)** | |
| --- | --- | --- | --- | --- | --- | --- | --- | --- | --- |
|  |  | **(µg/ml)** | **Response** | **(µg/ml)** |  | **Response** | **(µg/ml)** | **Response** | **(µg/ml)** |
| 1 | IM20 | 11.24 | positive | 0.153 | 2.5% | positive | 0.013 | negative |  |
| 2 | IM20 | 22.26 | positive | 0.116 | 4.0% | positive | 0.015 | positive | 0.014 |
| 3 | IM20 | 23.47 | n/a* | n/a | n/a | positive | 0.039 | positive | 0.020 |
| 4 | IM20 | 12.07 | positive | 0.214 | 4.6% | negative | n/a | negative | n/a |
| 5 | IM100 | 10.10 | positive | 0.113 | 8.7% | negative | n/a | negative | n/a |
| 6 | IM100 | 1.23 | positive | 0.099 | 3.6% | negative | n/a | negative | n/a |
| 7 | IM100 | 7.23 | positive | 0.078 | 1.5% | negative | n/a | negative | n/a |
| 8 | IM100 | 6.48 | negative | n/a | n/a | positive | 0.017 | negative | n/a |

**^Note:^** ^Mean values µg/ml (range)^
