# Supplementary material for: Comparative Immunogenicity of HIV-1 gp140 Vaccine Delivered by Parenteral, and Mucosal Routes in Female Volunteers; MUCOVAC2, A Randomized Two Centre Study
Source: PLoS One. 2016 May 9;11(5):e0152038. doi: 10.1371/journal.pone.0152038 (PMC4861263; doi:10.1371/journal.pone.0152038)
Supplement: S1 Text — (DOCX) [file pone.0152038.s008.docx]

**S1: Text Supporting Methods:**

**Processing of mucosal samples:**

Genital tract secretions were collected using the INSTEAD Softcup™ (Evofem Inc) or Weck-Cel® surgical spears (Medtronic). The Softcup was inserted into the upper vagina, left in place for at least one hour, removed and stored at -80C prior to processing. Softcups are not recommended when using an intrauterine contraceptive device (IUCD), and for those participants with an IUCD *in situ*, two Weck-Cel® surgical spears (Medtronic) were inserted into the vagina for 2 minutes at the same time-points. Prior to analysis, Softcups containing secretions in 50ml tubes were removed from -80^0^C, allowed to thaw on ice, and centrifuged at 400g for 15 minutes at 4^0^C. An equal volume of extraction buffer [250 mM NaCl, 1× protease inhibitor cocktail set 1 (Calbiochem) in phosphate buffered saline (1XDPBS)] was added to the secretion samples (after removal of the cup), mixed thoroughly and aliquoted then analysed directly by ELISA, with additional aliquots frozen at -80^0^C. Similarly Spin–X tubes containing Weck-Cel® spears were thawed on ice, centrifuged at 13,000rpm in a micro-centrifuge at 4^0^C. 300µl extraction buffer was added to the top chamber of the Spin–X tube containing the spears and centrifuged again for an additional 15 minutes at 13,000rpm and 4^0^C. The spears were removed and eluates in the bottom chamber were analysed directly by ELISA with remaining eluates aliquoted and frozen at -80^0^C.

**Anti-CN54gp140 specific antibody ELISA**

Serum and mucosal binding antibodies against recombinant CN54gp140 were measured using a standardized ELISA with minor modifications. 96 well ELISA plates were coated with 50µl per well of capture antigen CN54gp140 (1µg/ml) (Polymun). Human standards (either IgG or human IgA [from colostrum]) were captured by coating wells with a combination of α-Human κ and α-Human λ (1:1 ratio) capture antibodies. After incubation at 37^0^C for 1 hour, plates were washed with PBST then blocked for 1 hour at 37^0^C with 200µl/well of assay buffer then washed - as above. Standards were prepared by adding the required concentration of either human IgG or IgA. Serum samples were screened at 1:100 dilution, Softcup samples at 1:10 and Weck-Cel® samples at 1:2. Samples, standards and negative controls (normal human sera) were added to triplicate wells. Detection antibodies were added following incubation and washing, either goat α-Human IgG-HRP or goat α-Human IgA-HRP detection antibodies. After incubation and washing plates were developed by the addition of TMB substrate (KPL) followed by addition of 50µl of Stop Solution (KPL). Absorbencies were read immediately at 450nm using a VersaMax plate-reader (Molecular Devices). A response detected for both IgG and IgA was defined as OD A450nm value >0.2; samples below this value were deemed negative or response not detected. Samples were further diluted following screening assays if positive with a series of dilutions in order to extrapolate a concentration expressed as µg/ml of specific IgG or IgA using the ELISA software SoftMax Pro v 5.4. Specific activity in mucosal samples was calculated as: Specific activity µg/ml = [ELISA units per ml (specific titre)]/[total Ig µg/ml of same isotype] x 100.
